# Supplementary material for: Temperament and sexual behaviour in the Furrowed Wood Turtle Rhinoclemmys areolata
Source: PLoS One. 2020 Dec 30;15(12):e0244561. doi: 10.1371/journal.pone.0244561 (PMC7773281; doi:10.1371/journal.pone.0244561)
Supplement: S1 Table — The first column shows the names of each individual, bolder individuals have a grey backgrounds while those considered shier have white. (DOCX) [file pone.0244561.s001.docx]

**S1 Table**

|  | **Kicking during manipulation**  **(K)** | | **Neck stretched during manipulation**  **(NeSM)** | | **Hiding inside shell during manipulation**  **(HSM)** | | **Neck retracted during manipulation**  **(NeRM)** | | **Straightening time during depredation**  **(STD)** | |
| --- | --- | --- | --- | --- | --- | --- | --- | --- | --- | --- |
|  | **Fast kicking**  **(FK)** | **Slow kicking**  **(SK)** | **Yes neck stretched manipulation**  **(YNeSM)** | **No neck stretched manipulation**  **(NoNeSM)** | **Yes hide shell manipulation**  **(YHSM)** | **No hide shell manipulation**  **(NoHSM)** | **Yes neck retracted manipulation**  **(YNeRM)** | **No neck retracted manipulation**  **(NoNeRM)** | **High straightening time depredation**  **(HSTD)** | **Low straightening time depredation**  **(LSTD)** |
| Alfredo | 0 | 1 | 1 | 0 | 0 | 1 | 0 | 1 | 1 | 0 |
| Benedicto | 0 | 1 | 1 | 0 | 0 | 1 | 0 | 1 | 0 | 1 |
| Carlos | 0 | 1 | 1 | 0 | 0 | 1 | 0 | 1 | 1 | 0 |
| John | 1 | 0 | 1 | 0 | 0 | 1 | 0 | 1 | 0 | 1 |
| Erik | 1 | 0 | 0 | 1 | 1 | 0 | 1 | 0 | 0 | 1 |
| Nestor | 0 | 1 | 1 | 0 | 0 | 1 | 0 | 1 | 0 | 1 |
| Garry | 1 | 0 | 1 | 0 | 1 | 0 | 1 | 0 | 0 | 1 |
| Hector | 0 | 1 | 1 | 0 | 0 | 1 | 1 | 0 | 0 | 1 |
| Oliver | 0 | 1 | 0 | 1 | 0 | 1 | 1 | 0 | 0 | 1 |
| Ian | 0 | 1 | 1 | 0 | 0 | 1 | 0 | 1 | 0 | 1 |
| Kevin | 1 | 0 | 0 | 1 | 0 | 1 | 1 | 0 | 1 | 0 |
| Lorenzo | 0 | 1 | 0 | 1 | 0 | 1 | 1 | 0 | 0 | 1 |
| Marc | 0 | 1 | 1 | 0 | 0 | 1 | 0 | 1 | 0 | 1 |
| Patricio | 1 | 0 | 0 | 1 | 0 | 1 | 1 | 0 | 1 | 0 |
| Denis | 0 | 1 | 1 | 0 | 0 | 1 | 1 | 0 | 1 | 0 |
| Francesco | 0 | 1 | 1 | 0 | 0 | 1 | 1 | 0 | 1 | 0 |

…continue

|  | **Hiding inside shell during depredation (HSD)** | | **Walking time in new environment**  **(WTNE)** | | **Hiding in new environment**  **(HNE)** | | **Quite time in new environment**  **(QTNE)** | | **Crossing new environment (CrNE)** | |
| --- | --- | --- | --- | --- | --- | --- | --- | --- | --- | --- |
|  | **Yes hide shell depredation**  **(YHSD)** | **No hide shell depredation**  **(NoHSD)** | **High walking time new environment**  **(HWTNE)** | **Low walking time new environment**  **(LWTNE)** | **Yes hide new environment**  **(YHNE)** | **No hide new environment**  **(NoHNE)** | **High quite time new environment**  **(HQTNE)** | **Low quite time new environment**  **(LQTNE)** | **Yes cross area**  **(YCrNE)** | **No cross area**  **(NoCrNE)** |
| Alfredo | 1 | 0 | 0 | 1 | 0 | 1 | 1 | 0 | 1 | 0 |
| Benedicto | 0 | 1 | 1 | 0 | 0 | 1 | 0 | 1 | 1 | 0 |
| Carlos | 1 | 0 | 0 | 1 | 0 | 1 | 0 | 1 | 1 | 0 |
| John | 0 | 1 | 1 | 0 | 0 | 1 | 0 | 1 | 1 | 0 |
| Erik | 0 | 1 | 1 | 0 | 0 | 1 | 0 | 1 | 1 | 0 |
| Nestor | 1 | 0 | 1 | 0 | 0 | 1 | 0 | 1 | 1 | 0 |
| Garry | 1 | 0 | 1 | 0 | 0 | 1 | 0 | 1 | 0 | 1 |
| Hector | 1 | 0 | 1 | 0 | 0 | 1 | 0 | 1 | 0 | 1 |
| Oliver | 1 | 0 | 1 | 0 | 0 | 1 | 0 | 1 | 1 | 0 |
| Ian | 0 | 1 | 0 | 1 | 1 | 0 | 1 | 0 | 0 | 1 |
| Kevin | 1 | 0 | 0 | 1 | 1 | 0 | 1 | 0 | 0 | 1 |
| Lorenzo | 0 | 1 | 0 | 1 | 1 | 0 | 1 | 0 | 0 | 1 |
| Marc | 1 | 0 | 0 | 1 | 0 | 1 | 1 | 0 | 0 | 1 |
| Patricio | 0 | 1 | 0 | 1 | 1 | 0 | 1 | 0 | 0 | 1 |
| Denis | 1 | 0 | 0 | 1 | 0 | 1 | 1 | 0 | 1 | 0 |
| Francesco | 1 | 0 | 0 | 1 | 0 | 1 | 0 | 1 | 0 | 1 |

…continue

|  | **Exploration stuffed toy (ExST)** | | **Exploring time stuffed toy**  **(ExTST)** | | **Stretched Legs Manipulation**  **(SLM)** | | **Move immediately new environment**  **(YMINE)** | | **Ignore Stuffed toy**  **(IgnST)** | |
| --- | --- | --- | --- | --- | --- | --- | --- | --- | --- | --- |
|  | **Yes exploring stuffed toy**  **(YExST)** | **No exploring stuffed toy**  **(NoExST)** | **High time exploring stuffed toy**  **(HExTST)** | **Low time exploring stuffed toy**  **(LExTST)** | **Yes Stretched Legs Manipulation**  **(YSLM)** | **No Stretched Legs Manipulation**  **(NoSLM)** | **Yes Move immediately new environment**  **(YMINE)** | **No Move immediately new environment**  **(NoMINE)** | **Yes Ignore Stuffed toy**  **(YIgnST)** | **No Ignore Stuffed toy**  **(NoIgnST)** |
| Alfredo | 1 | 0 | 0 | 1 | 0 | 1 | 0 | 1 | 0 | 1 |
| Benedicto | 1 | 0 | 0 | 1 | 0 | 1 | 1 | 0 | 0 | 1 |
| Carlos | 0 | 1 | 1 | 0 | 0 | 1 | 1 | 0 | 1 | 0 |
| John | 1 | 0 | 1 | 0 | 0 | 1 | 1 | 0 | 1 | 0 |
| Erik | 1 | 0 | 1 | 0 | 1 | 0 | 1 | 0 | 0 | 1 |
| Nestor | 0 | 1 | 1 | 0 | 0 | 1 | 0 | 1 | 1 | 0 |
| Garry | 1 | 0 | 0 | 1 | 0 | 1 | 1 | 0 | 0 | 1 |
| Hector | 1 | 0 | 1 | 0 | 0 | 1 | 1 | 0 | 0 | 1 |
| Oliver | 1 | 0 | 1 | 0 | 0 | 1 | 1 | 0 | 1 | 0 |
| Ian | 0 | 1 | 1 | 0 | 0 | 1 | 1 | 0 | 0 | 1 |
| Kevin | 1 | 0 | 0 | 1 | 1 | 0 | 1 | 0 | 0 | 1 |
| Lorenzo | 1 | 0 | 1 | 0 | 0 | 1 | 0 | 1 | 0 | 1 |
| Marc | 0 | 1 | 1 | 0 | 0 | 1 | 0 | 1 | 1 | 0 |
| Patricio | 1 | 0 | 0 | 1 | 0 | 1 | 0 | 1 | 0 | 1 |
| Denis | 0 | 1 | 1 | 0 | 0 | 1 | 0 | 1 | 1 | 0 |
| Francesco | 0 | 1 | 1 | 0 | 0 | 1 | 1 | 0 | 1 | 0 |

…continue

|  | **Attack Stuffed toy**  **(AtkP)** | | **Push Stuffed toy**  **(PuP)** | |
| --- | --- | --- | --- | --- |
|  | **Yes Attack Stuffed toy**  **(YAtkST)** | **No Attack Stuffed toy**  **(NoAtkST)** | **Yes Push Stuffed toy**  **(YPuST)** | **No Push Stuffed toy**  **(NoPuST)** |
| Alfredo | 1 | 0 | 1 | 0 |
| Benedicto | 0 | 1 | 0 | 1 |
| Carlos | 0 | 1 | 1 | 0 |
| John | 1 | 0 | 1 | 0 |
| Erik | 0 | 1 | 1 | 0 |
| Nestor | 0 | 1 | 0 | 1 |
| Garry | 0 | 1 | 1 | 0 |
| Hector | 0 | 1 | 1 | 0 |
| Oliver | 0 | 1 | 0 | 1 |
| Ian | 0 | 1 | 1 | 0 |
| Kevin | 0 | 1 | 0 | 1 |
| Lorenzo | 0 | 1 | 1 | 0 |
| Marc | 0 | 1 | 0 | 1 |
| Patricio | 0 | 1 | 1 | 0 |
| Denis | 0 | 1 | 0 | 1 |
| Francesco | 0 | 1 | 1 | 0 |
