# Supplementary material for: Temperament and sexual behaviour in the Furrowed Wood Turtle Rhinoclemmys areolata
Source: PLoS One. 2020 Dec 30;15(12):e0244561. doi: 10.1371/journal.pone.0244561 (PMC7773281; doi:10.1371/journal.pone.0244561)
Supplement: S2 Table — Individuals considered bolder have grey backgrounds while those considered shier have white. (DOCX) [file pone.0244561.s002.docx]

**S2 Table**

| **Turtle Name** | **Straight Carapace Length (SCL)** | **Straight Plastron length (SPL)** |
| --- | --- | --- |
| Alfredo | 144,31 | 136,05 |
| Benedicto | 160,61 | 154,54 |
| Carlos | 148,74 | 128,04 |
| John | 139,14 | 121,54 |
| Erik | 153,7 | 140,33 |
| Nestor | 135,56 | 129,24 |
| Garry | 144,43 | 131,07 |
| Hector | 148,8 | 130,07 |
| Oliver | 152,21 | 143 |
| Ian | 135,78 | 125,19 |
| Kevin | 181,68 | 162,54 |
| Lorenzo | 137,37 | 117,6 |
| Marc | 142,22 | 135,15 |
| Patricio | 146,8 | 127,08 |
| Denis | 142,5 | 128,33 |
| Francesco | 137,43 | 124 |
