# Supplementary material for: Temperament and sexual behaviour in the Furrowed Wood Turtle Rhinoclemmys areolata
Source: PLoS One. 2020 Dec 30;15(12):e0244561. doi: 10.1371/journal.pone.0244561 (PMC7773281; doi:10.1371/journal.pone.0244561)
Supplement: S3 Table — The first column shows the names of each individual. Bolder individuals have grey backgrounds while shier individuals have white. (DOCX) [file pone.0244561.s003.docx]

**S3 Table**

|  | **Frontal approach with stretched legs and stretched neck**  **(FASLSN)** | | | **Frontal approach with stretched legs and retracted neck**  **(FASLRN)** | | | **Cross snouts**  **(CrS)** | | |
| --- | --- | --- | --- | --- | --- | --- | --- | --- | --- |
| **Name** | **High Frequency (HFASLSN)** | **Low Frequency (LFASLSN)** | **No Approach (NoFASLSN)** | **High Frequency (HFASLRN)** | **Low Frequency (LFASLRN)** | **No Approach (NoFASLRN)** | **High Frequency (HCrS)** | **Low Frequency (LCrS)** | **No Cross (NoCS)** |
| Alfredo | 0 | 1 | 0 | 0 | 0 | 1 | 0 | 1 | 0 |
| Benedicto | 0 | 0 | 1 | 0 | 1 | 0 | 0 | 0 | 1 |
| Carlos | 0 | 1 | 0 | 0 | 0 | 1 | 0 | 0 | 1 |
| Erik | 0 | 1 | 0 | 0 | 1 | 0 | 0 | 1 | 0 |
| Garry | 0 | 1 | 0 | 0 | 1 | 0 | 0 | 1 | 0 |
| Hector | 1 | 0 | 0 | 0 | 1 | 0 | 0 | 1 | 0 |
| John | 1 | 0 | 0 | 0 | 0 | 1 | 0 | 1 | 0 |
| Nestor | 0 | 1 | 0 | 0 | 0 | 1 | 0 | 1 | 0 |
| Oliver | 0 | 1 | 0 | 0 | 1 | 0 | 0 | 0 | 1 |
| Denis | 0 | 1 | 0 | 1 | 0 | 0 | 0 | 0 | 1 |
| Francesco | 0 | 1 | 0 | 0 | 0 | 1 | 0 | 1 | 0 |
| Ian | 1 | 0 | 0 | 1 | 0 | 0 | 0 | 1 | 0 |
| Kevin | 0 | 0 | 1 | 0 | 1 | 0 | 0 | 0 | 1 |
| Lorenzo | 0 | 1 | 0 | 0 | 0 | 1 | 1 | 0 | 0 |
| Marc | 0 | 1 | 0 | 0 | 0 | 1 | 0 | 1 | 0 |
| Patricio | 0 | 1 | 0 | 0 | 1 | 0 | 0 | 1 | 0 |

…continue

|  | **Push ground with front leg**  **(PGL)** | | | **Block the female**  **(Block)** | | **Pushing the female**  **(Push)** | | **Walk backwards**  **(WB)** | | |
| --- | --- | --- | --- | --- | --- | --- | --- | --- | --- | --- |
| **Name** | **High Frequency (HPGL)** | **Low Frequency (LPGL)** | **No Push (NoPGL)** | **Yes Blocking (YBlock)** | **No Blocking (NoBlock)** | **Yes Pushing (YPush)** | **No Pushing (NoPush)** | **High Frequency (HWB)** | **Low Frequency (LWB)** | **No Walk (NoWB)** |
| Alfredo | 0 | 1 | 0 | 0 | 1 | 0 | 1 | 0 | 1 | 0 |
| Benedicto | 0 | 1 | 0 | 0 | 1 | 0 | 1 | 0 | 1 | 0 |
| Carlos | 0 | 1 | 0 | 0 | 1 | 0 | 1 | 0 | 1 | 0 |
| Erik | 0 | 0 | 1 | 0 | 1 | 0 | 1 | 1 | 0 | 0 |
| Garry | 0 | 1 | 0 | 0 | 1 | 0 | 1 | 0 | 1 | 0 |
| Hector | 1 | 0 | 0 | 1 | 0 | 1 | 0 | 0 | 1 | 0 |
| John | 0 | 1 | 0 | 0 | 1 | 0 | 1 | 0 | 1 | 0 |
| Nestor | 0 | 0 | 1 | 0 | 1 | 0 | 1 | 0 | 0 | 1 |
| Oliver | 0 | 0 | 1 | 0 | 1 | 0 | 1 | 0 | 1 | 0 |
| Denis | 0 | 0 | 1 | 1 | 0 | 0 | 1 | 0 | 1 | 0 |
| Francesco | 0 | 1 | 0 | 1 | 0 | 1 | 0 | 0 | 1 | 0 |
| Ian | 1 | 0 | 0 | 0 | 1 | 0 | 1 | 1 | 0 | 0 |
| Kevin | 0 | 0 | 1 | 0 | 1 | 1 | 0 | 0 | 1 | 0 |
| Lorenzo | 0 | 0 | 1 | 0 | 1 | 0 | 1 | 0 | 1 | 0 |
| Marc | 0 | 1 | 0 | 0 | 1 | 1 | 0 | 0 | 0 | 1 |
| Patricio | 0 | 0 | 1 | 0 | 1 | 0 | 1 | 0 | 1 | 0 |

…continue

|  | **Excited male is approaching (EMA)** | | **Quite male explores surroundings**  **(QMExp)** | | **Cloaca sniffing**  **(CS)** | | **Remote tracking**  **(RT)** | | **Put leg on female**  **(PutLF)** | |
| --- | --- | --- | --- | --- | --- | --- | --- | --- | --- | --- |
| **Name** | **Yes excited male (YEMA)** | **No excited male (NoEMA)** | **Yes Explore (YQMExp)** | **No Explore (NoQMExp)** | **High Frequency (HCS)** | **Low Frequency (LCS)** | **Yes Remote (YRT)** | **No Remote (NoRT)** | **Yes Put leg on female**  **(YPutLF)** | **No Put leg on female**  **(NoPutLF)** |
| Alfredo | 0 | 1 | 1 | 0 | 1 | 0 | 0 | 1 | 1 | 0 |
| Benedicto | 0 | 1 | 0 | 1 | 0 | 1 | 0 | 1 | 1 | 0 |
| Carlos | 0 | 1 | 1 | 0 | 0 | 1 | 0 | 1 | 0 | 1 |
| Erik | 0 | 1 | 0 | 1 | 1 | 0 | 1 | 0 | 0 | 1 |
| Garry | 1 | 0 | 0 | 1 | 0 | 1 | 0 | 1 | 1 | 0 |
| Hector | 0 | 1 | 0 | 1 | 1 | 0 | 0 | 1 | 0 | 1 |
| John | 0 | 1 | 1 | 0 | 1 | 0 | 1 | 0 | 0 | 1 |
| Nestor | 0 | 1 | 0 | 1 | 0 | 1 | 0 | 1 | 0 | 1 |
| Oliver | 1 | 0 | 0 | 1 | 1 | 0 | 0 | 1 | 0 | 1 |
| Denis | 0 | 1 | 0 | 1 | 0 | 1 | 0 | 1 | 1 | 0 |
| Francesco | 1 | 0 | 1 | 0 | 0 | 1 | 0 | 1 | 1 | 0 |
| Ian | 1 | 0 | 0 | 1 | 1 | 0 | 0 | 1 | 0 | 1 |
| Kevin | 0 | 1 | 0 | 1 | 1 | 0 | 0 | 1 | 0 | 1 |
| Lorenzo | 0 | 1 | 0 | 1 | 1 | 0 | 0 | 1 | 0 | 0 |
| Marc | 0 | 1 | 0 | 1 | 1 | 0 | 0 | 1 | 1 | 0 |
| Patricio | 0 | 1 | 0 | 1 | 1 | 0 | 1 | 0 | 0 | 1 |

…continue

|  | **Frontal sniffing**  **(FrSn)** | | | **Lost interest**  **(LstInt)** | | | **Lateral Sniffing**  **(LatSn)** | | | **Tracking Time**  **(TrT)** | |
| --- | --- | --- | --- | --- | --- | --- | --- | --- | --- | --- | --- |
| **Name** | **High Frequency (HFrSn)** | **Low Frequency**  **(LFrSn)** | **No Frontal Sniffing**  **(NoFrSn)** | **High Frequency**  **(HLstInt)** | **Low Frequency**  **(LLstInt)** | **No Lost Interest**  **(NoLstInt)** | **High Frequency**  **HLatSn** | **Low Frequency**  **LLatSn** | **No Lateral Sniffing**  **NoLatSn** | **High Time**  **HTrT** | **Low Time**  **LTrT** |
| Alfredo | 0 | 0 | 1 | 0 | 0 | 1 | 0 | 1 | 0 | 0 | 1 |
| Benedicto | 0 | 0 | 1 | 0 | 0 | 1 | 0 | 1 | 0 | 0 | 1 |
| Carlos | 0 | 0 | 1 | 0 | 0 | 1 | 0 | 1 | 0 | 1 | 0 |
| Erik | 0 | 0 | 1 | 0 | 1 | 0 | 0 | 0 | 1 | 1 | 0 |
| Garry | 1 | 0 | 0 | 0 | 1 | 0 | 0 | 1 | 0 | 1 | 0 |
| Hector | 0 | 0 | 1 | 0 | 1 | 0 | 1 | 0 | 0 | 1 | 0 |
| John | 0 | 0 | 1 | 0 | 0 | 1 | 1 | 0 | 0 | 1 | 0 |
| Nestor | 1 | 0 | 0 | 0 | 0 | 1 | 0 | 1 | 0 | 0 | 1 |
| Oliver | 0 | 0 | 1 | 0 | 1 | 0 | 0 | 1 | 0 | 0 | 1 |
| Denis | 0 | 1 | 0 | 0 | 0 | 1 | 0 | 0 | 1 | 1 | 0 |
| Francesco | 0 | 0 | 1 | 0 | 1 | 0 | 1 | 0 | 0 | 0 | 1 |
| Ian | 1 | 0 | 0 | 1 | 0 | 0 | 0 | 1 | 0 | 0 | 1 |
| Kevin | 0 | 1 | 0 | 0 | 0 | 1 | 1 | 0 | 0 | 0 | 1 |
| Lorenzo | 0 | 1 | 0 | 0 | 0 | 1 | 0 | 1 | 0 | 1 | 0 |
| Marc | 0 | 1 | 0 | 0 | 0 | 1 | 0 | 1 | 0 | 1 | 0 |
| Patricio | 0 | 1 | 0 | 0 | 1 | 0 | 0 | 1 | 0 | 0 | 1 |
