# Supplementary material for: Temperament and sexual behaviour in the Furrowed Wood Turtle Rhinoclemmys areolata
Source: PLoS One. 2020 Dec 30;15(12):e0244561. doi: 10.1371/journal.pone.0244561 (PMC7773281; doi:10.1371/journal.pone.0244561)
Supplement: S4 Table — The first column shows the names of each individual, bolder individuals have grey backgrounds while shier have white. (DOCX) [file pone.0244561.s004.docx]

**S4 Table**

|  | **Coitus** | | **Neck Stimulation** | | | **Mount attempt** | | | **Mount** | | |
| --- | --- | --- | --- | --- | --- | --- | --- | --- | --- | --- | --- |
| **Name** | **YCo** | **NoCo** | **NoNS** | **LNS** | **HNS** | **NoMA** | **LMA** | **HMA** | **NoM** | **LM** | **HM** |
| Alfredo | 0 | 1 | 1 | 0 | 0 | 0 | 0 | 1 | 1 | 0 | 0 |
| Benedicto | 1 | 0 | 1 | 0 | 0 | 0 | 0 | 1 | 0 | 1 | 0 |
| Carlos | 0 | 1 | 0 | 0 | 1 | 0 | 0 | 1 | 0 | 0 | 1 |
| Erik | 0 | 1 | 0 | 1 | 0 | 1 | 0 | 0 | 0 | 1 | 0 |
| Garry | 0 | 1 | 0 | 1 | 0 | 0 | 0 | 1 | 0 | 1 | 0 |
| Hector | 0 | 1 | 1 | 0 | 0 | 0 | 1 | 0 | 1 | 0 | 0 |
| John | 0 | 1 | 1 | 0 | 0 | 1 | 0 | 0 | 1 | 0 | 0 |
| Nestor | 0 | 1 | 0 | 0 | 1 | 0 | 1 | 0 | 0 | 0 | 1 |
| Oliver | 0 | 1 | 1 | 0 | 0 | 0 | 1 | 0 | 1 | 0 | 0 |
| Denis | 0 | 1 | 1 | 0 | 0 | 1 | 0 | 0 | 1 | 0 | 0 |
| Francesco | 0 | 1 | 0 | 1 | 0 | 0 | 1 | 0 | 0 | 1 | 0 |
| Ian | 0 | 1 | 1 | 0 | 0 | 0 | 1 | 0 | 1 | 0 | 0 |
| Kevin | 0 | 1 | 1 | 0 | 0 | 0 | 0 | 1 | 1 | 0 | 0 |
| Lorenzo | 0 | 1 | 1 | 0 | 0 | 1 | 0 | 0 | 1 | 0 | 0 |
| Marc | 0 | 1 | 1 | 0 | 0 | 1 | 0 | 0 | 1 | 0 | 0 |
| Patricio | 0 | 1 | 0 | 1 | 0 | 0 | 0 | 1 | 0 | 0 | 1 |
