# Supplementary material for: Temperament and sexual behaviour in the Furrowed Wood Turtle Rhinoclemmys areolata
Source: PLoS One. 2020 Dec 30;15(12):e0244561. doi: 10.1371/journal.pone.0244561 (PMC7773281; doi:10.1371/journal.pone.0244561)
Supplement: S5 Table — The first column shows the names of each individual, bolder individuals have red backgrounds while shier have blue. Columns 2,3,4 and 5 indicate pairs by temperament (BB = boldVSbold, BS = boldVSshy, SS = shyVSshy, SB = shyVSbold). (DOCX) [file pone.0244561.s005.docx]

**S5 Table**

| **Name** | **BB** | **BS** | **SS** | **SB** | **Approaching to cloaca (AC)** | **Stretching neck towards female (SN)** | **Cloaca sniffing (CS)** | **Lateral sniffing (LS)** | **Tracking (T)** | **Quiet alongside female (Q)** | **Bite (Bi)** | **Blocking female (Bl)** | **Lean leg to female (LL)** | **Walk backwards (WB)** | **Push female (PF)** | **Push ground with leg (PGL)** |
| --- | --- | --- | --- | --- | --- | --- | --- | --- | --- | --- | --- | --- | --- | --- | --- | --- |
| Alfredo | 1 | 0 | 0 | 0 | 0 | 0 | 1 | 1 | 1 | 0 | 1 | 0 | 0 | 0 | 1 | 0 |
| Benedicto | 0 | 1 | 0 | 0 | 0 | 0 | 0 | 0 | 0 | 0 | 0 | 0 | 0 | 1 | 0 | 0 |
| Carlos | 0 | 1 | 0 | 0 | 1 | 1 | 1 | 1 | 1 | 1 | 0 | 0 | 1 | 1 | 0 | 0 |
| Erik | 1 | 0 | 0 | 0 | 0 | 1 | 1 | 0 | 1 | 1 | 0 | 0 | 1 | 0 | 1 | 0 |
| Garry | 1 | 0 | 0 | 0 | 0 | 0 | 1 | 0 | 1 | 1 | 1 | 0 | 0 | 1 | 0 | 0 |
| Hector | 0 | 1 | 0 | 0 | 0 | 1 | 1 | 1 | 1 | 1 | 0 | 0 | 1 | 1 | 1 | 1 |
| John | 0 | 1 | 0 | 0 | 1 | 1 | 1 | 1 | 1 | 1 | 1 | 0 | 1 | 0 | 0 | 0 |
| Nestor | 0 | 0 | 0 | 0 | 0 | 1 | 0 | 0 | 0 | 0 | 0 | 0 | 0 | 1 | 0 | 0 |
| Oliver | 1 | 0 | 0 | 0 | 0 | 1 | 1 | 1 | 1 | 0 | 1 | 0 | 1 | 1 | 0 | 0 |
| Denis | 0 | 0 | 1 | 0 | 0 | 1 | 0 | 1 | 0 | 0 | 0 | 1 | 0 | 0 | 0 | 0 |
| Francesco | 0 | 0 | 0 | 1 | 1 | 1 | 1 | 0 | 1 | 1 | 0 | 0 | 0 | 1 | 0 | 0 |
| Ian | 0 | 0 | 0 | 1 | 0 | 1 | 1 | 1 | 1 | 0 | 1 | 0 | 0 | 1 | 0 | 0 |
| Kevin | 0 | 0 | 1 | 0 | 0 | 1 | 1 | 0 | 1 | 1 | 0 | 1 | 1 | 0 | 0 | 0 |
| Lorenzo | 0 | 0 | 0 | 1 | 0 | 0 | 0 | 0 | 0 | 0 | 0 | 0 | 0 | 0 | 0 | 1 |
| Marc | 0 | 0 | 0 | 1 | 0 | 1 | 1 | 1 | 1 | 1 | 0 | 0 | 0 | 0 | 0 | 0 |
| Patricio | 0 | 0 | 0 | 1 | 0 | 1 | 1 | 1 | 1 | 0 | 0 | 0 | 0 | 1 | 1 | 0 |
