# Supplementary material for: Temperament and sexual behaviour in the Furrowed Wood Turtle Rhinoclemmys areolata
Source: PLoS One. 2020 Dec 30;15(12):e0244561. doi: 10.1371/journal.pone.0244561 (PMC7773281; doi:10.1371/journal.pone.0244561)
Supplement: S7 Table — (DOCX) [file pone.0244561.s007.docx]

**S7 Table**

| **Variable** | **Bolder** | | **Shier** | |  |  |
| --- | --- | --- | --- | --- | --- | --- |
|  | **mean** | **SE** | **mean** | **SE** | **U** | **p-value** |
| *Straight Carapace Length (SCL)* | 147,5 | 2,54890499 | 146,254286 | 6,07915715 | 20 | 0,24427761 |
| *Straight Plastron Length (SPL)* | 134,875556 | 3,28659783 | 131,412857 | 5,55408975 | 18 | 0,20400909 |
