# Supplementary material for: Temperament and sexual behaviour in the Furrowed Wood Turtle Rhinoclemmys areolata
Source: PLoS One. 2020 Dec 30;15(12):e0244561. doi: 10.1371/journal.pone.0244561 (PMC7773281; doi:10.1371/journal.pone.0244561)
Supplement: S8 Table — (DOCX) [file pone.0244561.s008.docx]

**S8 Table**

| **Variable** | **Bolder** | | **Shier** | |  |  |
| --- | --- | --- | --- | --- | --- | --- |
|  | **mean** | **SE** | **mean** | **SE** | **U** | **p** |
| Crossing a new environment (CrNE) | 0,777777778 | 0,146986184 | 0,142857143 | 0,142857143 | 12 | 0,0174* |
| Exploration of stuffed toy (ExST) | 0,777777778 | 0,146986184 | 0,428571429 | 0,202030509 | 21 | 0,1859 |
| Exploring time stuffed toy (ExTST) | 0,333333333 | 0,166666667 | 0,285714286 | 0,184427778 | 30 | 0,8953 |
| Hiding in a new environment (HNE) | 1 | 0 | 0,428571429 | 0,202030509 | 14 | 0,0137* |
| Hiding inside shell during manipulation (HSM) | 0,777777778 | 0,146986184 | 1 | 0 | 25 | 0,2306 |
| Hiding inside shell during predation (HSP) | 0,333333333 | 0,166666667 | 0,285714286 | 0,184427778 | 30 | 0,8953 |
| Kicking during manipulation (KM) | 0,666666667 | 0,166666667 | 0,714285714 | 0,184427778 | 30 | 0,8953 |
| Neck retracted during manipulation (NeRM) | 0,555555556 | 0,175682092 | 0,285714286 | 0,184427778 | 23 | 0,3253 |
| Neck stretched during manipulation (NeSM) | 0,777777778 | 0,146986184 | 0,571428571 | 0,202030509 | 25 | 0,4298 |
| Quiet time in new environment (QTNE) | 0,888888889 | 0,111111111 | 0,142857143 | 0,142857143 | 8 | 0,0047* |
| Straightening time during predation (STP) | 0,888888889 | 0,111111111 | 0,428571429 | 0,202030509 | 17 | 0,0654 |
| Walking time in a new environment (WTNE) | 0,777777778 | 0,146986184 | 0 | 0 | 7 | 0,0032* |
