# Supplementary material for: Temperament and sexual behaviour in the Furrowed Wood Turtle Rhinoclemmys areolata
Source: PLoS One. 2020 Dec 30;15(12):e0244561. doi: 10.1371/journal.pone.0244561 (PMC7773281; doi:10.1371/journal.pone.0244561)
Supplement: Attachment — Submitted filename: DCPONE-D-20-14068_reviewer.pdf [file pone.0244561.s011.pdf]

PLOS ONE

Temperament and sexual behaviour in the Furrowed Wood Turtle Rhinoclemmys areolata

--Manuscript Draft--

**Manuscript Number:**	PONE-D-20-14068
**Article Type:**	Research Article
**Full Title:**	Temperament and sexual behaviour in the Furrowed Wood Turtle Rhinoclemmys areolata
**Short Title:**	~~Personality~~ a[illegible]sex in a terrestrial turtle
**Corresponding Author:**	Yann Henaut, Ph.D.<br>El Colegio de la Frontera Sur<br>Chetumal, Quintana Roo MEXICO
**Keywords:**	~~personality;~~ agonistic behavior; reproduction; terrestrial turtle
**Abstract:**	Summary<br><br>Temperament variability in animals have consequences for evolution and ecology. One of the primary effects of consistent behavioral differences points to reproduction. In chelonians some works have focused on the study of temperament using different methods for its measurement. In our research we first wanted to i) establish a methodology to determine boldness degree, in the turtle Rhinoclemmys areolata . Secondly, we wanted to ii) determine the role boldness plays during reproduction, with emphasis on courtship and copula, considering a) the interactions between male and female, and b) the competition between males. We used 16 sexually mature individuals of each sex. We first observed the males in four different situations and recorded 17 behavioral traits. We selected 12 traits that allowed us to determine the bolder and the shier individuals. We found that five behavioral traits were specific for bolder individuals and five others for the shier. In a second step, we observed a male in presence of a female and recorded courtship behaviors and breeding attempts. Bolder individuals did not display courtship behaviors and just attempt to copulate. Shier individuals displayed courtship behaviors and rare copula attempts. Finally, we observed two males in presence of one female and noticed that bolder individuals then displayed courtship behaviors while the shier just ignore the female. Our results first allowed us to determine which methodology is the best to determine temperament in turtles. Secondly, the temperament seems to be an important factor to modulate interaction between males and females. ~~Bolder individuals have an advantage during competition and display courtship behaviours only if other males are present.~~ Shier males displayed courtship behaviors and try to copulate only when no competitors were present. Those two different temperament-dependant strategies are discussed in terms of ecology, evolution and management.
**Order of Authors:**	Francesca Maura Cassola
	Yann Henaut, Ph.D.
	José Rogelio Cedeño-Vázquez
	Fausto Roberto Méndez-de la Cruz
	Benjamín Morales-Vela
**Additional Information:**	
**Question**	**Response**
**Financial Disclosure**<br><br>Enter a financial disclosure statement that describes the sources of funding for the work included in this submission. Review	CFM and CVJR. CONACYT-OEA-AMEXCID scholarship program for the funds needed to cover expenses for data collection and specimen maintenance.

Powered by Editorial Manager® and ProduXion Manager® from Aries Systems Corporation

the submission guidelines for detailed requirements. View published research articles from *PLOS ONE* for specific examples.<br><br>This statement is required for submission and **will appear in the published article** if the submission is accepted. Please make sure it is accurate.<br><br>**Unfunded studies**<br>Enter: *The author(s) received no specific funding for this work.*<br><br>**Funded studies**<br>Enter a statement with the following details:<br>• Initials of the authors who received each award<br>• Grant numbers awarded to each author<br>• The full name of each funder<br>• URL of each funder website<br>• Did the sponsors or funders play any role in the study design, data collection and analysis, decision to publish, or preparation of the manuscript?<br>• **NO** - Include this sentence at the end of your statement: *The funders had no role in study design, data collection and analysis, decision to publish, or preparation of the manuscript.*<br>• **YES** - Specify the role(s) played.<br><br>* typeset	
**Competing Interests**<br><br>Use the instructions below to enter a competing interest statement for this submission. On behalf of all authors, disclose any competing interests that could be perceived to bias this work—acknowledging all financial support and any other relevant financial or non-financial competing interests.<br><br>This statement **will appear in the published article** if the submission is accepted. Please make sure it is accurate. View published research articles from *PLOS ONE* for specific examples.	The authors have declared that no competing interests exist.

**NO authors have competing interests**<br><br>Enter: *The authors have declared that no competing interests exist.*<br><br>**Authors with competing interests**<br><br>Enter competing interest details beginning with this statement:<br><br>*I have read the journal's policy and the authors of this manuscript have the following competing interests: [insert competing interests here]*<br><br>* typeset	
**Ethics Statement**<br><br>Enter an ethics statement for this submission. This statement is required if the study involved:<br><br>• Human participants<br>• Human specimens or tissue<br>• Vertebrate animals or cephalopods<br>• Vertebrate embryos or tissues<br>• Field research<br><br>Write "N/A" if the submission does not require an ethics statement.<br><br>General guidance is provided below. Consult the submission guidelines for detailed instructions. **Make sure that all information entered here is included in the Methods section of the manuscript.**	TThe investigation was carried out in compliance with the institutional ethical standards and norms in force. All the individuals collected and on loan were destined exclusively for specific research purpose, respecting all known needs for animal welfare. Experimental protocol was approved by Ethical Committee from the “El Colegio de la Frontera Sur”, Mexico.

Powered by Editorial Manager® and ProduXion Manager® from Aries Systems Corporation

**Format for specific study types**<br><br>**Human Subject Research (involving human participants and/or tissue)**<br>• Give the name of the institutional review board or ethics committee that approved the study<br>• Include the approval number and/or a statement indicating approval of this research<br>• Indicate the form of consent obtained (written/oral) or the reason that consent was not obtained (e.g. the data were analyzed anonymously)<br><br>**Animal Research (involving vertebrate animals, embryos or tissues)**<br>• Provide the name of the Institutional Animal Care and Use Committee (IACUC) or other relevant ethics board that reviewed the study protocol, and indicate whether they approved this research or granted a formal waiver of ethical approval<br>• Include an approval number if one was obtained<br>• If the study involved *non-human primates*, add *additional details* about animal welfare and steps taken to ameliorate suffering<br>• If anesthesia, euthanasia, or any kind of animal sacrifice is part of the study, include briefly which substances and/or methods were applied<br><br>**Field Research**<br><br>Include the following details if this study involves the collection of plant, animal, or other materials from a natural setting:<br>• Field permit number<br>• Name of the institution or relevant body that granted permission	
**Data Availability**<br><br>Authors are required to make all data underlying the findings described fully available, without restriction, and from the time of publication. PLOS allows rare exceptions to address legal and ethical concerns. See the PLOS Data Policy and FAQ for detailed information.	Yes - all data are fully available without restriction

Powered by Editorial Manager® and ProduXion Manager® from Aries Systems Corporation

A Data Availability Statement describing where the data can be found is required at submission. Your answers to this question constitute the Data Availability Statement and **will be published in the article**, if accepted.<br><br>**Important:** Stating 'data available on request from the author' is not sufficient. If your data are only available upon request, select 'No' for the first question and explain your exceptional situation in the text box.<br><br>Do the authors confirm that all data underlying the findings described in their manuscript are fully available without restriction?	
**Describe where the data may be found in full sentences. If you are copying our sample text, replace any instances of XXX with the appropriate details.**<br><br>• If the data are **held or will be held in a public repository**, include URLs, accession numbers or DOIs. If this information will only be available after acceptance, indicate this by ticking the box below. For example: *All XXX files are available from the XXX database (accession number(s) XXX, XXX.)*.<br>• If the data are all contained **within the manuscript and/or Supporting Information files**, enter the following: *All relevant data are within the manuscript and its Supporting Information files.*<br>• If neither of these applies but you are able to provide **details of access elsewhere**, with or without limitations, please do so. For example:<br><br>*Data cannot be shared publicly because of [XXX]. Data are available from the XXX Institutional Data Access / Ethics Committee (contact via XXX) for researchers who meet the criteria for access to confidential data.*<br><br>*The data underlying the results presented in the study are available from (include the name of the third party*	All relevant data are within the manuscript and its Supporting Information files.

Powered by Editorial Manager® and ProduXion Manager® from Aries Systems Corporation

and contact information or URL).<br>• This text is appropriate if the data are owned by a third party and authors do not have permission to share the data.<br><br>* typeset	
Additional data availability information:	

Manuscript Click here to access/download;Manuscript;Manuscript.docx

Temperament and sexual behaviour in the Furrowed Wood Turtle *Rhinoclemmys areolata*

Short title: ~~Personality~~ and sex in a terrestrial turtle

Cassola FM [1], Henaut Y [1*], Cedeño-Vázquez JR [1], Méndez-de la Cruz FR[2], Morales-Vela B[1]

[1]El Colegio de la Frontera Sur, Unidad Chetumal, Chetumal, Quintana Roo, México

[2]Laboratorio de Herpetología, Departamento de Zoología, Instituto de Biología, Universidad Nacional Autónoma de México. México.

*Corresponding Author

e-mail: yhenaut@ecosur.mx

Summary

Temperament variability in animals have consequences for evolution and ecology. One of the primary effects of consistent behavioral differences points to reproduction. In chelonians some works have focused on the study of temperament using different methods for its measurement. In our research we first wanted to i) establish a methodology to determine boldness degree, in the turtle *Rhinoclemmys areolata*. Secondly, we wanted to ii) determine the role boldness plays during reproduction, with emphasis on courtship and copula, considering a) the interactions between male and female, and b) the competition between males. We used 16 sexually mature individuals of each sex. We first observed the males in four different situations and recorded 17 behavioral traits. We selected 12 traits that allowed us to determine the bolder and the shier individuals. We found that five behavioral traits were specific for bolder individuals and five others for the shier. In a second step, we observed a male in presence of a female and recorded courtship behaviors and breeding attempts. Bolder individuals did not display courtship behaviors and just attempt to copulate. Shier individuals displayed courtship behaviors and rare copula attempts. Finally, we observed two males in presence of one female and noticed that bolder individuals then displayed courtship behaviors while the shier just ignore the female. Our results first allowed us to determine which methodology is the best to determine temperament in turtles. Secondly, the temperament seems to be an important factor to modulate interaction between males and females. ~~Bolder individuals have an advantage during competition and display courtship behaviours only if other males are present. Shier males displayed courtship behaviors and try to copulate only when no competitors were present.~~ Those two different temperament-dependant strategies are discussed in terms of ecology, evolution and management.

Key-words: ~~personality,~~ agonistic behavior, reproduction, terrestrial turtle temperament

INTRODUCTION

Inter-individual behavioral differences in an animal species are consistent or largely maintained over time and in diverse situations [1] and sometimes between one population and the other [2]. These differences are what some scientists call “temperament”, “behavioral syndrome” or “personality” [3-4] and they have been attributed to the combined influences of genetic, social and environmental factors that permanently affect the behavior of an individual [5].

Animal behavioral differences can have important implications for fitness [6-7] and have been shown to influence ecological processes such as dispersion, invasion, response to climate change and risk of extinction [8-10]. There is a high probability that temperament has a great influence on the way in which animals respond to new environments (for example, new exhibits or retention areas), to familiar and unfamiliar conspecifics, to strangers of other species, and to changes in their environment, affecting social compatibility, stability and success of a group [11]. Animal behavior and temperament can influence breed[illegible]n capacity by directly connecting to a successful match (success of coupling) or the number and viability of offspring produced (reproductive success) by the specimens [12-13]. Since temperament affects the way in which individuals react to difficult situations, it can be predicted how a male can compete, select and be compatible with a sexual partner. Therefore, variations in individual behavior are a source of information in the field of sexual selection and reproductive success [6].

Individuals may systematically differ in one or more temperament traits, such as boldness, aggressiveness, reactivity, sociability, exploration or activity levels [1]. A feature that has been extensively studied in the field of temperament is boldness defined as the inclination of individuals to explore their environment and take risks in a new situation [14]. Boldness and shyness are considered as a continuum [14-16] at one extreme we find shier specimens and on the opposite side the bolder ones.

This spectrum seems to have an adaptive explanation in terms of costs and benefits, related to the type of response to a risk. In general, bolder individuals tend to sustain high reproductive success but at a potential cost for their survival, while shy individuals engage in opposite behaviors. This implies that temperament would allow to regulate survival with other needs of the species [15].

There are few studies addressing the relationship between success of couples and behavioral compatibility. Knowing these individual differences provides essential information for captivity management and animal welfare [15,11]. For example, it allows us to understand why some specimens have reproductive problems in captivity and why they fail to satisfy their reproductive potential, even if they are physically and physiologically in health [17]. A few years ago, in fact, specimens suitable for mating were chosen based on their hypothetical genetic compatibility [18-19], their state of health [20] and / or stages of life history [21-22]. However, couples who represent a good genetic combination do not necessarily produce offspring, and behavioral incompatibility is often cited for this failure [23-26]. One way in which animal temperament can influence mating probability is through the effects associated with behavioral compatibility the way individuals interact with each other [12]. When animals are paired for reproduction, some temperaments may be compatible, while others may be in conflict. For some species, couples with similar temperaments may be more suitable while, in other cases, ~~couples with opposite temperaments may have the highest probability of reproductive success~~ [13]. ~~If a couple is incompatible from a behavioral point of view and is not well matched, the potential genetic benefits of offspring production are completely lost for the population [27].~~
Recently, boldness has been studied in aquatic turtles ~~and the study shows that the~~ recognition between males and the reduction of competitive interactions are related to the boldness of males [28]. They showed that the response to chemical stimuli of known or

unknown males of the Mediterranian Turtle *Mauremys leprosa*, depends on the level of boldness, in particular the shiest males are those who avoid encountering other males, of the same or different species, as it could help stabilize social systems and reduce the frequency and intensity of aggressive encounters between males. Different methods are used to evaluate temperament in turtles. ~~Boldness~~ level is measured using the straightening response, which is the time it takes for an individual to straighten up after being turned upside down on his carapace [29]. A similar method has been used to study anti-predator responses in the European pond turtle *Emys orbicularis* [30]. Another method consists to insert specimens in a new environment: here, the latency to move from an initial position in an arena was used to evaluate exploration in the red-eared slider turtle *Trachemys scripta* [31] and in the Eastern hermann *Eurotestudo boettgeri* [32]. The presentation of a threatening stimuli was also used to measure the ~~boldness~~ and the effect of new objects on investigative behaviors to measure exploration in Agassiz´s desert tortoise (*Gopherus agassizii*) [4]. The boldness in the Eastern box turtle (*Terrapene carolina*) was evaluated using the time to emerge from the shell and the time to move after a short period of confinement [33]. Therefore, there are different methods applicable to turtles but none of the aforementioned studies tried to apply more than one of them to a single species. For this reason, ~~attempt to create~~ a unique methodology for the study of boldness in turtles is still lacking.

Unfortunately, there is a lack of work addressing specifically how temperament may be related to the reproductive behavior of turtles, particularly if it influences male competition behavior, courtship and coupling. Thus, the aims of this study were to (i) determinate temperament of *Rhinoclemmys areolata* and provide a method to determine the temperament in these species that can also be used for others terrestrial turtle species (ii)

understand if its temperament could be a factor that may modulate the interaction between male and female, and (iii) between males during the competition for a female.

MATERIAL AND METHODS

Object of study

For the experiment, we used 16 males and 16 females of the furrowed wood turtle *Rhinoclemmys areolata*, all sexually mature. This species occurs in Yucatán Peninsula, Cozumel Island, Northern Guatemala, Belize, North-western Honduras, where it inhabits savannah, thorny scrub, broad-leaved forests, swampy and gouache areas [34]. It is used as pet, food and in ~~the~~ traditional medicine [35]. It is listed as Near-threatened by the IUCN Red List [36]. It is also known as “Chak pool” or “Mojina” [35]. It is characterized by 1 to 2 red, orange or yellow stripes on the top of the head, and the plastron is yellow with a dark spot of variable size (Fig 1). It has a low level of sexual dimorphism, males having a longer and thinner tail than females, and also having a slightly more concave plastron but this is a characteristic that is not always detectable (Fig 2). It is a terrestrial species, whose only connection with the aquatic environment is during courtship and copulation; however, these derive from two anecdotic observations events where the specimens were kept in pools without the presence of a large terrestrial environment [37-38]. Courtship and copulation could be induced by spraying them with water [34].

Fig 1. Details of *Rhinoclemmys areolata* morphology.

Fig 2. Detail of female (left) and male (right). It can be seen the difference between the tails (red arrow), females have a shorter and thicker tail, while males have a longer and thinner one.

Turtles' management and setup

Specimens of *Rhinoclemmys areolata* were kept within a 16 x 5 m enclosure, located in the facilities of El Colegio de la Frontera del Sur (ECOSUR) Unidad Chetumal (18 ° 32'37.4"N, 88 ° 15 ' 48.0"W). The area was fenced and divided into two parts of approximately the same size (5 x 8 m) containing male and female specimens, respectively. This division avoid any reproductive activity in the absence of the observer. It also avoids female from recollecting semen from males and be less reactive to males [39].

The area inside the fence was as close as possible to the natural habitat of *R. areolata*, and it was possible by maintaining the presence of trees, foliage, natural soil and adding water sites (Fig 3).

Turtles were fed with fruit, vegetables and meat on alternate days. Inside the maintenance area, they were free to move, to find refuge and bathe in small pools. They were subject to natural temperatures and rainfall for as long as they were inside the area in order to keep the best environmental conditions for their welfare.

All simulations were performed in a mobile enclosed area (2 x 2 m) (Fig 4). Each simulation was recorded through two cameras (YiLiteCam and Motorola Camera). The first video camera was positioned above the observation area while the second was moved manually by the observer in case details of the events were needed.

Each simulation took place in open air, on sunny days, during hours of maximum activity of turtles (8.30am-11.30am and 05.30pm - 06.30pm), when the temperatures are not very high. Animals were separated after each observation session to avoid any additional intersexual interactions in absence of the observer. The observation area was moved in an adjacent area after every simulation to reduce waiting time between experiments and recovery time of environmental conditions, then a new group of turtles was placed to repeat simulation.

Fig 3. Captive living area of *Rhinoclemmys areolata* at the ECOSUR campus.

Fig 4. Observation area from the side and from the top views.

Experiments

Determination of temperament of *Rhinoclemmys areolata*.

It was necessary to identify stable behavioral traits over time and in different situations. For this reason, 16 males were observed individually and in isolation in four different situations:

- Reaction to manipulation: a male is taken from the confinement and handled briefly for 1 minute.
- Reaction to risk of predation: a male is taken from the confinement, handled briefly and gently released in an overturned position, that is, on his carapace.
- Reaction to a new environment: a male is taken from the confinement and released in experimental area unknown to the specimen. (30min)
- Reaction to the introduction of a new object: a male is taken from the confinement, released in experimental area and, after 5 minutes, an unknown object is suddenly released (stuffed dog-like animal). (30min)

To organize males in order to describe their boldness-shyness level we: (i) extrapolated behavioral events of each simulation from video recordings, (ii) converted them into variables, (iii) defined occurrence (=1) and absence (=0) of each event obtaining binomial values; (iv) defined frequency and (v) time in seconds when possible, obtaining quantitative values; (vi) for the latter, it was measured the moment of abrupt change of slope within data range, this way it was possible to classify male as shy or bold (S1 Table).

Each event was considered as a variable and inserted into an Excel sheet. A boldness index was given to each male, dividing the sum of "occurrences" of variables that could describe

a bold temperament by the total number of variables (Table 2). This analysis allowed us to classify specimens into groups, according to their degree of boldness.
Afterwards, variables were analysed using a correlation matrix and the obtained values were inserted into a multidimensional scaling (MDS) that allowed us to elaborate a map showing relative positions between different events and relative distances.
Finally, variables were analysed through a Mann-Whitney test to determine which ones were significant in determining temperament.

Before starting interaction experiments, we tested the influence of turtles' length (that is considered linked with the age of each individual) on temperament. We did this test to ensure results are not the consequence of the size or age of the turtles. The two groups obtained were analysed in relation to straight carapace length (SCL) and straight plastron length (SPL) through Mann-Whitney test (S2 Table).

Temperament as a modulating factor during the interaction between male and female.

16 groups of turtles consisting of one male and one female were created randomly. Each couple was released, sequentially, within the experimental area, each individual inside a metal box. They were soaked with water using an artificial sprayer to promote reproductive behavior and later freed to move around for 30 minutes.
For this experiment, the same specimens were not used more than once.
Each group remained sequentially in the experimental arena and was carefully observed.
As for the first simulations, we (i) extrapolated behavioral events from each simulation, (ii) converted them into variables, (iii) defined occurrence (=1) and absence (=0) of each event obtaining binomial values; (iv) defined frequency and (v) time in seconds when possible,

obtaining quantitative values; (vi) for the latter, it was measured the moment of abrupt change of slope within data range (S3 and S4 Table).

Each event was considered as a variable and inserted into an Excel sheet. Variables were analysed using a correlation matrix and the obtained values were inserted into a multidimensional scaling (MDS) that allowed us to elaborate a map showing relative positions between different events and relative distances.

Temperament as a modulating factor during the competitive interaction between males in the presence of a female.

Eight experimental groups were randomly created, each consisting of two males and one female. For this experiment, the same specimens will not be used more than once.

The specimens were placed equidistant within the experimental area, soaked with water using an artificial sprayer to promote reproductive behavior and freed to move around for 30 minutes.

Each group remained sequentially in the experimental area and was carefully observed.

As for the first simulations, we (i) extrapolated behavioral events from each simulation, (ii) converted them into variables, (iii) defined occurrence (=1) and absence (=0) of each event obtaining binomial values; (iv) defined frequency and (v) time in seconds when possible, obtaining quantitative values; (vi) for the latter, it was measured the moment of abrupt change of slope within data range (S5 and S6 Table).

Each event was considered as a variable and inserted into an Excel sheet. Variables were analysed using a correlation matrix and the obtained values were inserted into a multidimensional scaling (MDS) that allowed us to elaborate a map showing relative positions between different events and relative distances.

Ethical statement:

The investigation was carried out in compliance with the institutional ethical standards and norms in force. All the individuals collected and on loan were destined exclusively for specific research purpose, respecting all known needs for animal welfare. Experimental protocol was approved by Ethical Committee from the “El Colegio de la Frontera Sur”, Mexico.

RESULTS

Determination of temperament of *Rhinoclemmys areolata*

We observed 17 behavioral traits (S1 Table) but several of them were displayed by all the turtles and were not useful to distinguish temperaments; therefore, we selected 12 behavioral traits that were used as variables (Table 1). For each male, the presence or absence of each of them was defined (Table 2). For this study, we used “bolder” and “shier” terms instead of “bold” and “shy” since boldness is a continuous spectrum and it is not possible to create a sharp and fixed fractures for group definition.

In order to create two groups for the following experiments, we considered as shier all ~~organisms~~ that had an index less than or equal to 0.5, while bolder were those that had an index greater than 0.5. This allowed us to divide specimens into 9 bolder and 7 shier males (Table 2).

Straight carapace length and straight plastron length ~~didn't~~ show a significant relationship with temperament (Carapace: Mann-Whitney U test: U=20, P=0.25; Plastron: Mann-Whitney U test: U=19, P=0.21) (S7 Table).

Rearranging the variables according to presence, frequency and times (Table 1), and through the multidimensional scaling, we obtained a total of 10 variables: five correlated with the shiest individuals and other five associated with the boldest individuals (Fig 5).

These variables were ~~for~~ the shier: (i) hiding in a new environment, (ii) not crossing a new environment, (iii) high quiet time in a new environment, (iv) high straightening time during predation, and (v) low walking time in a new environment[illegible] for the boldest: (i) not hiding in a new environment, (ii) cross a new environment, (iii) low quiet time in a new environment, (iv) low straightening time during predation, and (v) high walking time in a new environment (Fig 5). Four of those variables were also statistically significant for the discrimination of the two groups (Fig 6, S8 Table).

Table 1. Variables selected for simulations, subdivision used in correlation analysis and multidimensional scaling with relative abbreviation.

Variable	Subdivision for analysis	Abbreviation
Kicking during manipulation (KM)	Fast kicking	FK
	Slow kicking	SK
Neck stretched during manipulation (NeSM)	Yes neck stretched manipulation	YNeSM
	No neck stretched manipulation	NoNeSM
Hiding inside shell during manipulation (HSM)	Yes hide shell manipulation	YHSM
	No hide shell manipulation	NoHSM
Neck retracted during manipulation (NeRM)	Yes neck retracted manipulation	YNeRM
	No neck retracted manipulation	NoNeRM
Straightening time during predation (STP)	High straightening time predation	HSTPr
	Low straightening time predation	LSTPr
Hiding inside shell during predation (HSP)	Yes hide shell predation	YHSPr
	No hide shell predation	NoHSPr
Walking time in a new environment (WTNE)	High walking time new environment	HWTNE
	Low walking time new environment	LWTNE
Hiding in a new environment (HNE)	Yes hide new environment	YHNE
	No hide new environment	NoHNE
Quiet time in new environment (QTNE)	High quiet time new environment	HQTNE
	Low quiet time new environment	LQTNE
Crossing a new environment (CrNE)	Yes cross area	YCrNE
	No cross area	NoCrNE
Exploration peluche (ExP)	Yes exploring peluche	YExP
	No exploring peluche	NoExP
Exploring time peluche (ExTP)	High time exploring peluche	HExTP
	Low time exploring peluche	LExTP

Table 2. Presence (=1) or absence (=0) of variables selected for simulation and boldness index values. In grey background color the specimens considered bolder, in white those considered shier.

Turtle Name	Sum variables	Boldness Index	KM	NeSM	HSM	NeRM	STD	HSPr	WTNE	HNE	QTNE	CrNE	ExP	ExTP
**Alfredo**	9	0,75	1	1	1	1	1	0	0	1	0	1	1	1
**Benedicto**	12	1	1	1	1	1	1	1	1	1	1	1	1	1
**Carlos**	8	0,67	1	1	1	1	1	0	0	1	1	1	0	0
**Hector**	8	0,67	1	1	1	0	1	0	1	1	1	0	1	0
**Nestor**	8	0,67	1	1	1	1	0	0	1	1	1	1	0	0
**Erik**	7	0,58	0	0	0	0	1	1	1	1	1	1	1	0
**Garry**	7	0,58	0	1	0	0	1	0	1	1	1	0	1	1
**John**	10	0,83	0	1	1	1	1	1	1	1	1	1	1	0
**Oliver**	8	0,67	1	0	1	0	1	0	1	1	1	1	1	0
**Francesco**	5	0,41	1	1	1	0	0	0	0	1	1	0	0	0
**Kevin**	3	0,25	0	0	1	0	0	0	0	0	0	0	1	1
**Marc**	5	0,41	1	1	1	1	0	0	0	1	0	0	0	0
**Patricio**	3	0,25	0	0	1	0	0	0	0	0	0	0	1	1
**Denis**	6	0,5	1	1	1	0	1	0	0	1	0	1	0	0
**Ian**	6	0,5	1	1	1	1	1	1	0	0	0	0	0	0
**Lorenzo**	5	0,41	1	0	1	0	1	1	0	0	0	0	1	0

Temperament as a modulating factor during the interaction between male and female

As results for the relation between temperaments and male and female interaction, we observed behaviors linked with courtship and others with mating.

Regarding courtship, we determined 16 behaviors (S3 Table), but we used 11 of them for the analysis, excluding behaviors showed by most of the specimens and the ones that did not show differences between the groups.

We defined if they occurred or not and how often to obtain their frequency (low or high) (Table 3). We observed that the following traits are linked with the shier individuals (Figure 7): (i) pushing the female, (ii) blocking the female, (iii) frontal approach with stretched legs and retracted neck (iv) pushing the ground with front leg, (v) approaching when excited. On the other hand, some bolder turtles did not show courtship behavior while in others they were not very frequent. Bolder individuals are linked with: (i) do not block the female, (ii) do not push it, (iii) do not approach excitedly, (iv) do not walk backwards, (v) low frequency of frontal approach with stretched legs and extended neck (Fig 7).

Four behaviors (Table 4, S4 Table) were observed for mating. None of those behaviors is displayed by shiest individuals, while the boldest are more linked with intent of mounts, and mount at high frequency (Fig 8, S1 Video).

Table 3. Courtship behaviors obtained from simulations male vs female, subdivision used in correlation analysis and must-dimensional scale with relative abbreviation.

Variable	Subdivision
Frontal approach with stretched legs and stretched neck	High Frequency (HFASLSN)<br>Low Frequency (LFASLSN)<br>No Approach (NoFASLSN)
Frontal approach with stretched legs and retracted neck	High Frequency (HFASLRN)<br>Low Frequency (LFASLRN)<br>No Approach (NoFASLRN)
Cross snouts	High Frequency (HCrS)<br>Low Frequency (LCrS)<br>No Cross (NoCrS)
Push ground with front leg	High Frequency (HPGL)<br>Low Frequency (LPGL)<br>No Push (NoPGL)
Block the female	Yes Blocking (YBlock)<br>No Blocking (NoBlock)
Pushing the female	Yes Pushing (YPush)<br>No Pushing (NoPush)
Walk backwards	High Frequency (HWB)<br>Low Frequency (LWB)<br>No Walk (NoWB)
Excited male is approaching	Yes excited male (YEMA)<br>No excited male (NoEMA)
Quiet male explores surroundings	Yes Explore (YQMExp)<br>No Explore (NoQMExp)
Cloaca sniffing	High Frequency (HCS)<br>Low Frequency (LCS)
Remote tracking	Yes Remote (YRT)<br>No Remote (NoRT)

Table 4. Mating behaviors obtained from simulations male vs female, subdivision used in correlation analysis and must-dimensional scale with relative abbreviation.

Variable	Subdivision
Neck stimulation	High Frequency (HNS)
	Low Frequency LNS)
	No Stimulation (NoNS)
Mount attempt	High Frequency (HMA)
	Low Frequency (LMA)
	No Mount attempt (NoMA)
Coitus	Yes Coitus (YCo)
	No Coitus (NoCo)
Mount	High Frequency (HM)
	Low Frequency (LM)
	No Mount (NoM)

S1 Video. Shy courtship and Bold mount.

Temperament as a modulating factor during the competitive interaction between males in the presence of a female.

We obtained 12 behaviors related to courtship (Table 5, S5 Table). We considered temperament of the two males together with a female and observed that courting variables are concentrated around the boldest regardless the temperament of their opponent (Fig 9).

On the contrary, the shiest did not display particular behaviors except for blocking the female during the tracking (Fig 9) (S2 Video).

Table 3. Courtship behaviors obtained from simulations male vs male vs female used in correlation analysis and must-dimensional scale with relative abbreviation.

Variable	Abbreviation
Approaching to cloaca	AC
Stretching neck towards female	SN
Cloaca sniffing	CS
Lateral sniffing	LS
Tracking	T
Quiet alongside female	Q
Bite	Bi
Blocking female	Bl
Lean leg to female	LL
Walk backwards	WB
Push female	PF
Push ground with leg	PGL

Fig 5. Distribution of the variables (behaviors) of the specimens. The red circle shows variables linked with the boldest specimens, the blue circle identifies behaviors linked to the shiest ones.

Fig 6. Mean values and standard errors of the four significant binomial variables. High time walking new environment, Bolder: mean±SE = 0,78±0,1, Shier: mean±SE= 0±0, Mann-Whitney U test: U=7, p=0.00*3; Not hiding new environment, Bolder: mean±SE = 1±0, Shier: mean±SE= 0,43±0,2, Mann-Whitney U test: U=13,5, p=0.013*; Low time quiet new

environment, Bolder: mean±SE = 0,89±0,1, Shier: mean±SE= 0,14±0,14, Mann-Whitney U test: U=37, p=0.004*; Cross new environment, Bolder: mean±SE = 0,78±0,14, Shier: mean±SE= 0,14±0,1, Mann-Whitney U test: U=37, p=0.017*.

Fig 7. Distribution of courtship variables (behaviors) of the male specimens. The red curve includes variables linked with boldest specimens, the blue curve congregates behaviors linked with the shiest individuals.

Fig 8. Distribution of mating variables (behaviors) of the male specimens. The red curve encompasses variables linked with boldest individuals, the blue curve includes behaviors linked with the shiest ones.

Fig. 9. Distribution of courtship variables (behaviors) of the specimens. The red circle delimits variables highly linked with boldest males in competition with each other, the yellow circle encompasses those highly linked with a bold male in competition with a shy one, the green ellipse includes behaviors linked with the boldest not considering who is its competitor.

S2 Video. Shy and bold male in presence of a female. Shy is walking around the arena while the bold courtships the female.

DISCUSSION

A variety of methods were used but separately to determine temperament in aquatic and terrestrial turtles [4,29-33]. This is the first paper that tests all of them. In this study, we tested reaction to: manipulation, predation risk, a new environment and the introduction of a novel object with the purpose of looking for consistency, to determine the most accurate behaviors and in consequence the best method to define the level of boldness in turtles. Behaviors observed in a new environment seem to be the best to determine the boldness in turtles: hiding and limited walk for the shiest, being quiet (shiest) or not (boldest), walking

a lot and crossing the new environment for the boldest. These results coincide with two of previous works [31-32].

The test with the risk of predation also shows that the time to straighten is linked with the boldness as observed in previous studies [29-30].

~~Also, our~~ work focused only on males, and the determination of temperament in females or juveniles may be different even if the exploration of a new environment may be equivalent whatever the sex and age of turtles that generally share the same strategy of life whatever their conditions. Therefore, to assure that behaviors linked with the reaction to a new environment can be a better method, we suggest further studies to test it also in females and juveniles.

An interesting element that emerges in this study is the modulation of males´ behavior.

When a bold male is alone with a female, he often skips courtship behaviors to devote himself to stimulating the female for mounting and mating; when the same male is found in the presence of another conspecific male, a potential sexual competitor, the first engages in courtship behaviors towards the female. A similar result has been observed in the common water strider *Aquarius remiges* (Gerridae, Insecta), where the most active and aggressive males spend most of their time looking for a female as well as having the highest copula rates [40], and in the common lizard *Zoocora vivipara* (Lacertidae, Reptilia), where females prefer the more active males when they find themselves alone and without the presence of predators in their surroundings, whereas when shier specimens find themselves alone in the presence of a female, they spend time and energy in courtship behaviour [41]. In our simulations, mating attempts of shier males were very rare; when the shier is in presence of a competitor and a female, it ignores the female. In summary, boldest individuals court the female only in presence of a potential competitor and directly mate with her without courtship if no competitor is present. Shier males courtship the female only if no

competitor is present and is not very effective at mating. This kind of behavior towards a competitor was observed in *Mauremys leprosa* where responses to chemicals cues depended on boldness; in particular, shy turtles avoided chemical cues from familiar and unfamiliar bolder males [29]. However, it is possible for ~~the~~ shier that more time is necessary to display a mating behavior, anyway our work shows apparent differences in the sexual behavior according to males' temperaments. Therefore, we can argue that the degree of boldness can influence males' reproductive behavior, in decision making and their reaction according to the situation. In terms of costs and benefits, boldness can be considered a factor that allows specimens to evaluate how to act during courtship and mating considering the presence of conspecifics of both sexes. On the other hand, it avoids competition and the possibility of losing energy when a bolder male is competing. Male competition be very costly because of fights, injuries, territory care and predatory danger during courtship and refraining those costs could also be an alternative strategy to avoid damages and predators, as shown in the Iberian wall lizard *Podarcis hispanica,* which uses chemical cues during competitive matches to recognize competitors and reduce intensity and costs of the fights [42]. The success of different strategies dependant of the temperament is under environmental constraint such as the presence of predators as observed for other animals [1]. For example, the broadhead skink *Eumeces* (*Plestiodon*) *laticeps* shows a sensitivity in predation risk during reproductive opportunities: when females were present, isolated and mate-guarding males initiate courtship, but isolated males permitted closer approach by a predator than did mate-guarding males. Moreover, there is a high level of latency by isolated males when a predator is close to the female so they were able to balance risk of predation with courtship behavior [43].

Researchers have already discussed that behavior plays a key role in animal survival and evolution, behavioral traits can help species to respond to rapid changes in the environment [44], and pass under the clutches of natural selection [45] as much as morphological and

physiological traits. Differences in sexual behavior related to boldness or other behavioral traits, can be an honest signal of mate quality [46]. Therefore, identifying those behavioral traits that can affect population viability is important to provide helpful information to integrate conservation plans [47].

The information obtained in this study represents an important tool for the conservation of reptiles and in particular for land turtles. Worldwide, turtles represent one of the vertebrate groups with the highest risk of extinction [48]. In recent years, many researchers have ensured the importance of not only increasing the behavioral information of the species, but also implementing these data in their conservation and monitoring strategies [11,49].

To our best knowledge, this is the first work that connects behavioral trait to reproductive behaviors in land turtles. What we have achieved can be used as a first step for compatibility plans for turtles, in order to combine temperament traits that might compose a successful breeding couple [11].

Temperament studies in couple compatibility have significant implications in *ex situ* conservation programs, since specific traits (such as aggressiveness and fear) have been related to reproductive success in some species raised in zoos [12]. In addition, certain temperaments may be more likely to thrive in captive environments, artificial selection for or against specific temperament traits may accelerate domestication processes, making animals raised for conservation less suitable for release in wildlife [24].

[illegible] a previous study it can be seen that compatible pairs (male-female) of the Cockatiel *Nymphicus hollandicus* showed higher levels of coordination in parental care and raised more chicks [50];[illegible] Black Rhino *Diceros bocornis* smaller enclosures determined the breeding of dominant males, while domination decreased in males housed in larger enclosures [23,51]. In addition, dominance levels were related to reproductive success, with the conclusion that the number of births per year was higher in pairs of rhinos composed of a submissive male and a dominant or aggressive female. This information gives an idea of

the optimal conditions for the breeding of captive rhinos, including large enclosures, which facilitate submissive behavior in males, which in turn affects the compatibility of couples; in the giant panda *Ailuropoda melanoleuca*, behavioral studies to further improve reproductive performance and found that specific combinations of temperament traits showed better reproductive performance than others [12]. Males who were more aggressive than their partners were more likely to mate and produce puppies than when the female had a higher level of aggression than the male or even couples with less fearful males, regardless of the fear of the female, obtained better reproductive results. The application of these results to captive breeding management strategies should result in higher reproductive rates and the production of more candidates for the giant panda reintroduction program.

Our research ~~focused on boldness degree of males~~ showed that bolder males need less time to mount and mate, ~~therefore they could have~~ a higher fitness than shier ones. ~~By another way in term of evolution, being shiest may have some advantages and, if not for reproduction, they may be for antipredatory behaviour, making shiest individuals, that are more prudent in term of exploration, and as we observed in the new environment, generally less predated than boldest individuals as discussed for other species [52].~~

Another application of our findings is the possibility to keep the whole range of boldness. Considering behavioral variation, it is important to preserve boldness since it allows conservationists to reintroduce animals with all the behavioural traits, giving them proper tools to survive in novel environments [45]. Considering this, in order to not to lose the behavioral pool during captive reproduction, we must be aware of the needs of different specimens. Therefore, our study ~~has allowed to understand~~ that shy turtles likely need more time for mating and need to be alone with the female, otherwise they lose interest. For the boldest, the necessary times are lower and they are potentially able to reproduce even with the presence of conspecifics.

Unfortunately, we do not know with which female temperament they are more compatible and therefore with which their copula level can increase. Therefore, we recommend to continue studying this species and its reproductive behaviour not only to increase knowledge which is very limited, but also to better understand relationship between temperament and reproduction in the view of turtle survival.

Acknowledgments

We would like to thank El Colegio de la Frontera del Sur and the CONACYT-OEA-AMEXCID scholarship program for the funds needed to cover expenses for data collection and specimen maintenance; the citizens of Chetumal who gave and loaned specimens of *R. areolata* for this research; José Alberto García Angulo for his help in the design and construction of the turtle maintenance and observation areas; Dr. Gabriele Gheza, Martina Anna Belli and Antonin Mareš for their help in grammatical and linguistic revision.

References

[1] Réale D, Reader S, Sol D, McDougall PT, Dingemanse NJ. Integrating animal temperament within ecology and evolution. Biol Rev. 2007;82: 291-318.

[2] Pervin L, John OP. Handbook of personality: theory and research. 2nd ed. New York: Guilford; 1999.

[3] MacKay JRD, Haskell MJ. Consistent individual behavioral variation: the difference between temperament, personality and behavioral syndromes. Animals. 2015;5: 455-478.

[4] Germano JM, Nafus MG, Perry JA, Hall DB, Swaisgood RR. Predicting translocation outcomes with personality for desert tortoises. Behav. Ecol. 2017;28: 1075-1084

[5] Dingemanse NJ, Araya-Ajoy, YG. Interacting personalities: behavioural ecology meets quantitative genetics. Trends Ecol. Evol. 2015;30: 88-97.

[6] Dingemanse NJ, Réale D. 2005. Natural selection and animal personality. Behavior. 2005;142: 1165-1190.

[7] Smith BR, Blumstein DT. Fitness consequences of personality: a meta-analysis. Behav Ecol. 2008;19: 448-455.

[8] Chapman JW, Klaassen RH, Drake VA, Fossette S, Hays GC, Metcalfe JD, Reynolds AM, Reynolds DR, Alerstam T. Animal orientation strategies for movement in flows. Curr. Biol. 2011;21: R861-R870.

[9] Duckworth RA, Badyaev AV. Coupling of dispersal and aggression facilitates the rapid range expansion of a passerine bird. Proc. Natl. Acad. Sci. U.S.A. 2007;104: 15017-15022.

[10] Dyer JRG, Croft DP, Morrell LJ, Krause J. Shoal composition determines foraging success in the guppy. Behav. Ecol. 2009;20: 165-171.

[11] Tetley CL, O'Hara SJ. Ratings of animal personality as a tool for improving the breeding, management and welfare of zoo mammals. Anim. Welf. 2012;21: 463-476.

[12] Martin-Wintle MS, Shepherdson D, Zhang G, Huang Y, Luo B, Swaisgood RR. Do opposites attract? Effects of personality matching in breeding pairs of captive giant pandas on reproductive success. Biol. Conserv. 2017;207: 27-37.

[13] Kelleher SR, Silla AJ, Byrne PG. Animal personality and behavioral syndromes in amphibians: a review of the evidence, experimental approaches, and implications for conservation. Behav. Ecol. Sociobiol. 2018;72: 79.

[14] Wilson ADM, Godin J-GJ. Boldness and behavioral syndromes in the bluegill sunfish, *Lepomis macrochirus*. Behav. Ecol. 2009;20: 231-237.

[15] Cole EF, Quinn JL. Shy birds play it safe: personality in captivity predicts risk responsiveness during reproduction in the wild. Biol. Lett. 2014;10: 20140178.

[16] White SJ, Pascall DJ, Wilson AJ. Towards a comparative approach to the structure of animal personality variation. Behav. Ecol. 2020;31: 340-351.

[17] Powell D, Lin H, Carlstead K, Kleiman D, Zhang H, Zhang G, et al. Relationships between temperament, husbandry, management, and socio-sexual behavior in captive male and female giant pandas (*Ailuropoda melanoleuca*). Acta Zool. Sin. 2008;54: 169-175.

[18] Schwartz MK. Guidelines on the use of molecular genetics in reintroduction programs. In: The EU LIFE-Nature Projects to guidelines for the reintroduction of threatened species. March 21-22; Caramanico Terme, Italy; 2005. p.51-58.

[29] Miller W, Wright SJ, Zhang Y, Schuster SC, Hayes VM. Optimization methods for selecting founder individuals for captive breeding or reintroduction of endangered species. In Pacific Symp. Biocomput. 2010: 43-53.

[20] Saidenberg ABS, Zuniga E, Melville PA, Salaberry S, Benites NR. Health-screening protocols for vinaceous amazons (*Amazona vinacea*) in a reintroduction project. J. Zoo Wildl. Med. 2015;46: 704-712.

[21] Kleiman DG. Foreword: animal behaviour studies and zoo propagation programs. Zoo Biol. 1994;13: 411-412.

[22] Bremner-Harrison S, Prodohl PA, Elwood RW. Behavioural trait assessment as a release criterion: boldness predicts early death in a reintroduction programme of captive-bred swift fox (*Vulpes velox*). Anim. Conserv. Forum. 2004; 313-320.

[23] Carlstead K, Mellen J, Kleiman DG. Black rhinoceros (*Diceros bicornis*) in US zoos: I. Individual behavior profiles and their relationship to breeding success. Zoo Biol. 1999a;18: 17-34.

[24] McDougall PT, Réale D, Sol D, Reader SM. 2006. Wildlife conservation and animal temperament: causes and consequences of evolutionary change for captive, reintroduced, and wild populations. Anim Conserv. 2006;9: 39-48.

[25] Freeman EW, Guagnano G, Olson D, Keele M, Brown JL. Social factors influence ovarian acyclicity in captive African elephants (*Loxodonta africana*). Zoo Biol. 2009;28: 1-15.

[26] Lees CM, Wilcken J. Sustaining the ark: the challenges faced by zoos in maintaining viable populations. Int. Zoo Yearb. 2009;43: 6-18.

[27] Cable RN. Oh, Behave: When wildlife behavior matters in Conservation. Mich. J. Sustain. 2013: 1

[28] Ibáñez A, Polo-Cavia N, López P, Martín J. Honest sexual signaling in turtles: experimental evidence of a trade-off between immune response and coloration in red-eared sliders *Trachemys scripta elegans*. Naturwissenschaften. 2014;101: 803-811

[29] Ibáñez A, Marzal A, López P, Martín J. Boldness and body size of male Spanish terrapins affect their responses to chemical cues of familiar and unfamiliar males. Behav. Ecol. Sociobiol. 2013;67: 541-548.

[30] Ibáñez A, Martín J, Gazzola A, Pellitteri-Rosa D. Freshwater turtles reveal personality traits in their antipredatory behaviour. Behav. Processes. 2018;157: 142-147.

[31] Carter AW, Paitz RT, McGhee KE, Bowden RM. Turtle hatchlings show behavioral types that are robust to developmental manipulations. Physiol. Behav. 2016;155: 46-55.

[32] Mafli A, Wakamatsu K, Roulin A. Melanin-based coloration predicts aggressiveness and boldness in captive Eastern hermann's tortoises. Anim. Behav. 2011;81: 859-863.

[33] Kashon EAF, Carlson BE. Consistently bolder turtles maintain higher body temperatures in the field but may experience greater predation risk. Behav. Ecol. Sociobiol. 2018;72: 9.

[34] Vogt RC, Platt, SG, Rainwater TR. *Rhinoclemmys areolata* (Duméril and Bibron 1851), Furrowed wood turtle, Black-bellied turtle, Mojina. Chelonian Res. Monogr. 2009;5: 22-27.

[35] Cupul Cicero V, Aguilar Cordero WDJ., Chablé Santos J, Sélem Salas CI. Conocimiento etnozoológico de la herpetofauna de la comunidad maya de Santa Elena, Yucatán, México. Estud. Cult. Maya. 2019;54: 285-314.

[36] van Dijk PP, Lee J, Calderón Mandujano R, Flores-Villela O, Lopez-Luna MA. *Rhinoclemmys areolata*. *The IUCN Red List of Threatened Species. 2007.*

[37] Pérez-Higareda G, Smith HM. Courtship behavior in *Rhinoclemmys Areolata* from western Tabasco, Mexico (Testudines: *Emydidae*). The Gt. Basin Nat. 1988;48: 263-266.

[38] Artner H. Field observations and captive breeding of the furrowed wood turtle *Rhinoclemmys areolata* (Duméril and Bibron, 1851). Emys. 2009;16: 4-19.

[39] Pearse DE, Avise JC. Turtle mating systems: behavior, sperm storage, and genetic paternity. J. Hered. 2001;92: 206-211.

[40] Sih, A, Bell, A, Johnson CJ, Ziemba RE. Behavioral syndromes: an integrative overview. Q. Rev. Biol. 2004;79: 241-277.

[41] Teyssier A, Elvire Bestion E, Richard M, Cotea J. Partners' personality types and mate preferences: predation risk matters. Behav. Ecol. 2014;25: 723-733.

[42] López P, Martín J. Chemical rival recognition decreases aggression levels in male Iberian wall lizards, *Podarcis hispanica*. Behav. Ecol. Sociobiol. 2002;51: 461-465.

[43] Cooper Jr. WE. Tradeoffs between courtship, fighting, and antipredatory behavior by a lizard, *Eumeces laticeps*. Behav. Ecol. Sociobiol. 1999;47: 54-59.

[44] Angeloni L, Crooks KR, Blumstein DT. Breed MD, Moore J, editors. Conservation and behavior: Introduction. 2010. pp. 377–381. Encyclopedia of animal behavior, Volume 1. Oxford: Academic Press.

[45] Caro T, Sherman PW. Endangered species and a threatened discipline: behavioural ecology. Trends Ecol. Evol. 2011;;26: 111–118.

[46] Merrick MJ, Koprowski JL. Should we consider individual behavior differences in applied wildlife conservation studies? Biol. Conserv. 2017;209: 34-44.

[47] Ahtiainen JJ, Alatalo RV, Mappes J, Vertainen L. Decreased sexual signalling reveals reduced viability in small populations of the drumming wolf spider *Hygrolycosa rubrofasciata*. Proc R Soc London B. 2004;271: 1839-1845.

[48] Rhodin AG, Stanford CB, Van Dijk PP, Eisemberg C, Luiselli L, Mittermeier RA, et al. Global conservation status of turtles and tortoises (Order *Testudines*). Chelonian Conserv. Biol. 2018;17: 135-161.

[49] Tadesse SA. Animal behavior in conservation biology. Intl. J. Avian Wildl. Biol. 2018;3: 25-26.

[50] Spoon TR, Millam JR, Owings DH. 2006. The importance of mate behavioural compatibility in parenting and reproductive success by cockatiels, *Nymphicus hollandicus.* Anim. Behav. 2006;71: 315-326.

[51] Carlstead K, Fraser J, Bennett C, Kleiman DG. Black rhinoceros (*Diceros bicornis*) in US zoos: II. Behavior, breeding success, and mortality in relation to housing facilities. Zoo Biol. 1999b;18: 35-52.

[52] Cooper Jr WE. Variation in escape behavior among individuals of the striped plateau lizard *Sceloporus virgatus* may reflect differences in boldness. J. Herpetol. 2009;43: 495-502.

Figure1

Click here to access/download;Figure;Fig 1. Details of Rhinoclemmys areolata

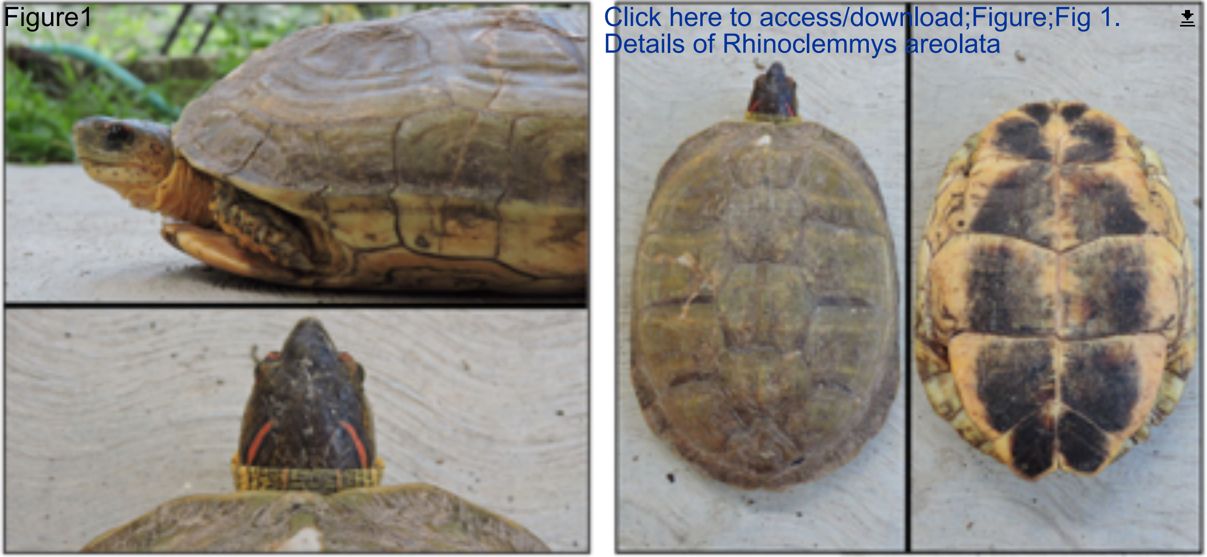

Figure2

Click here to access/download;Figure;Fig 2. Detail of female (left) and male (right).pdf

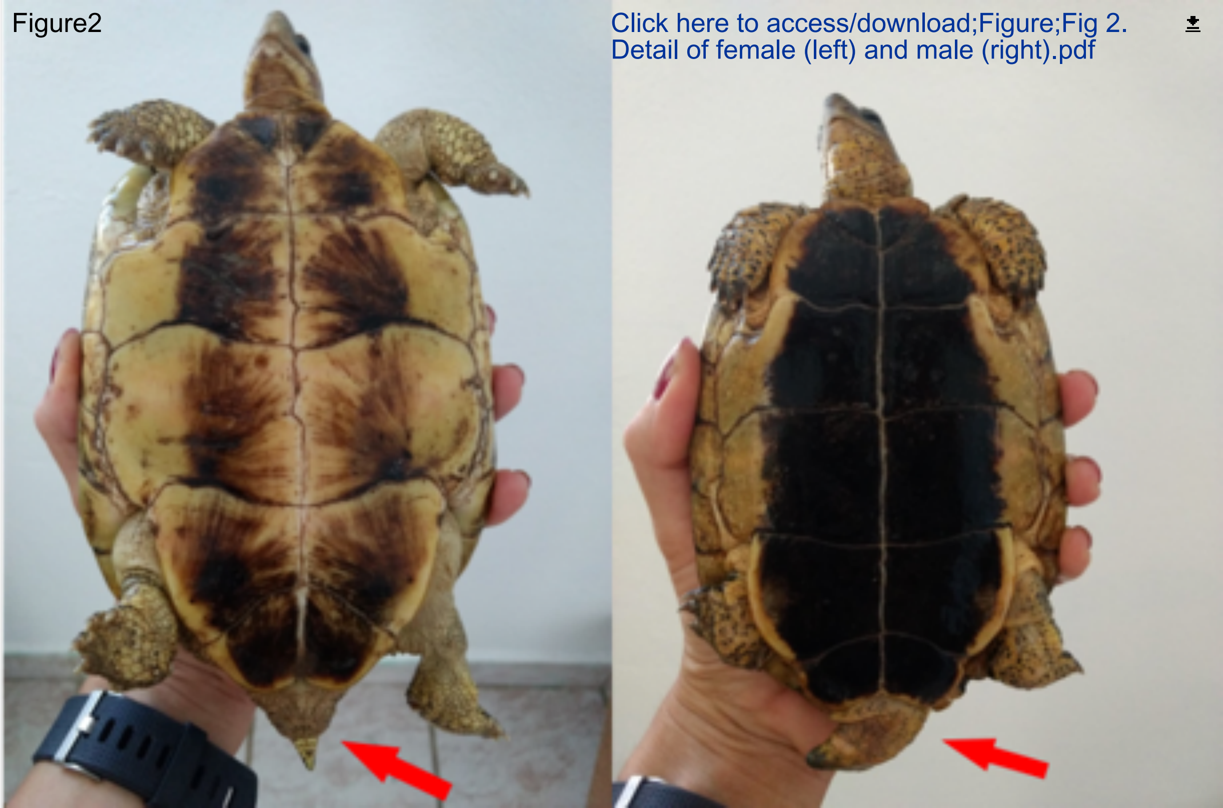

Figure3

Click here to access/download;Figure;Fig 3.pdf

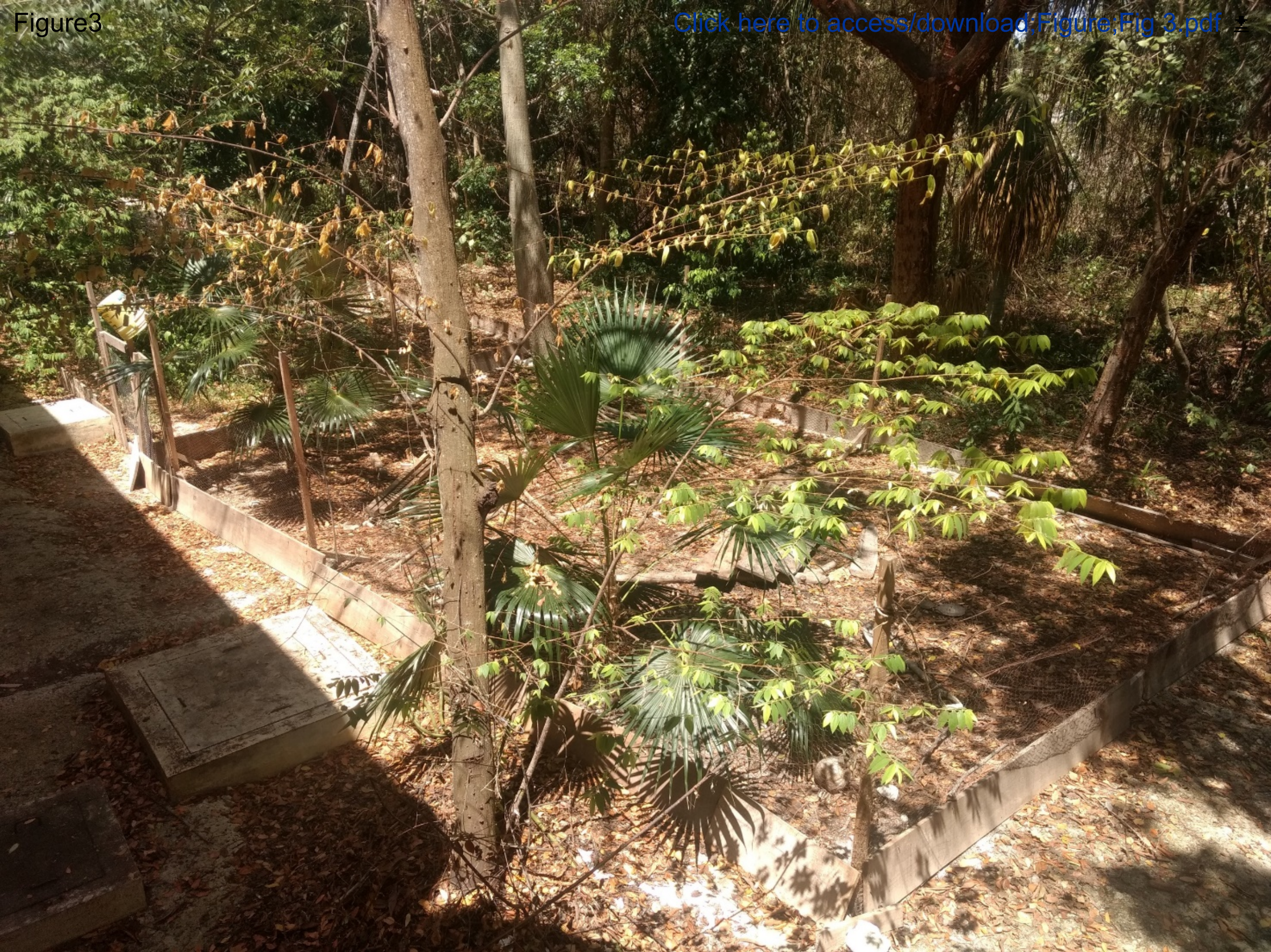

Figure4

Click here to access/download;Figure;Fig 4.pdf

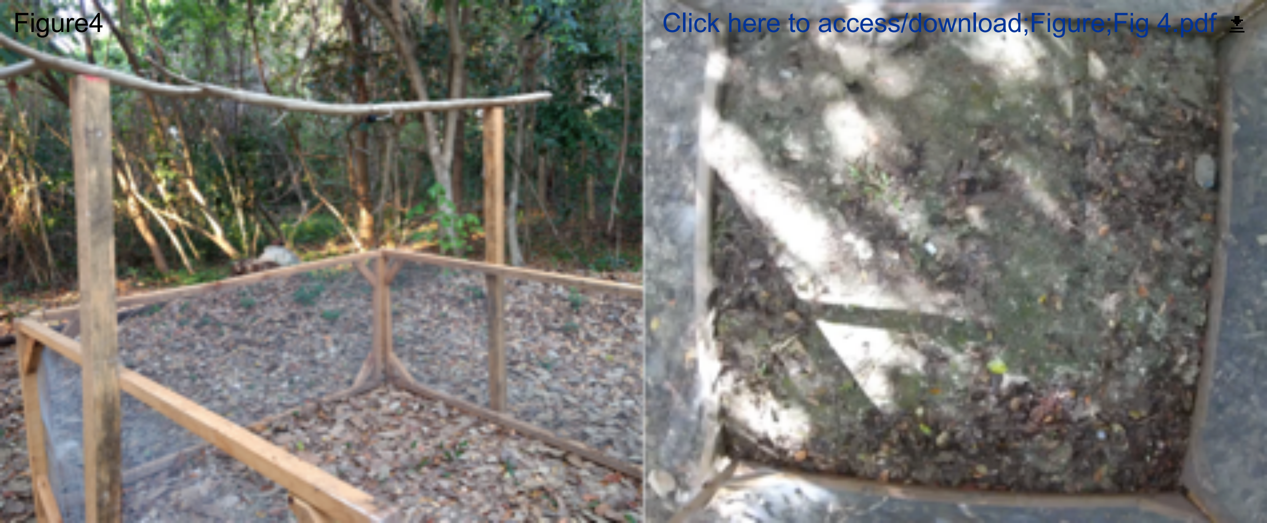

Click here to access/download;Figure;Fig 5.pdf

Scatterplot courtshitp behaviors male VS female

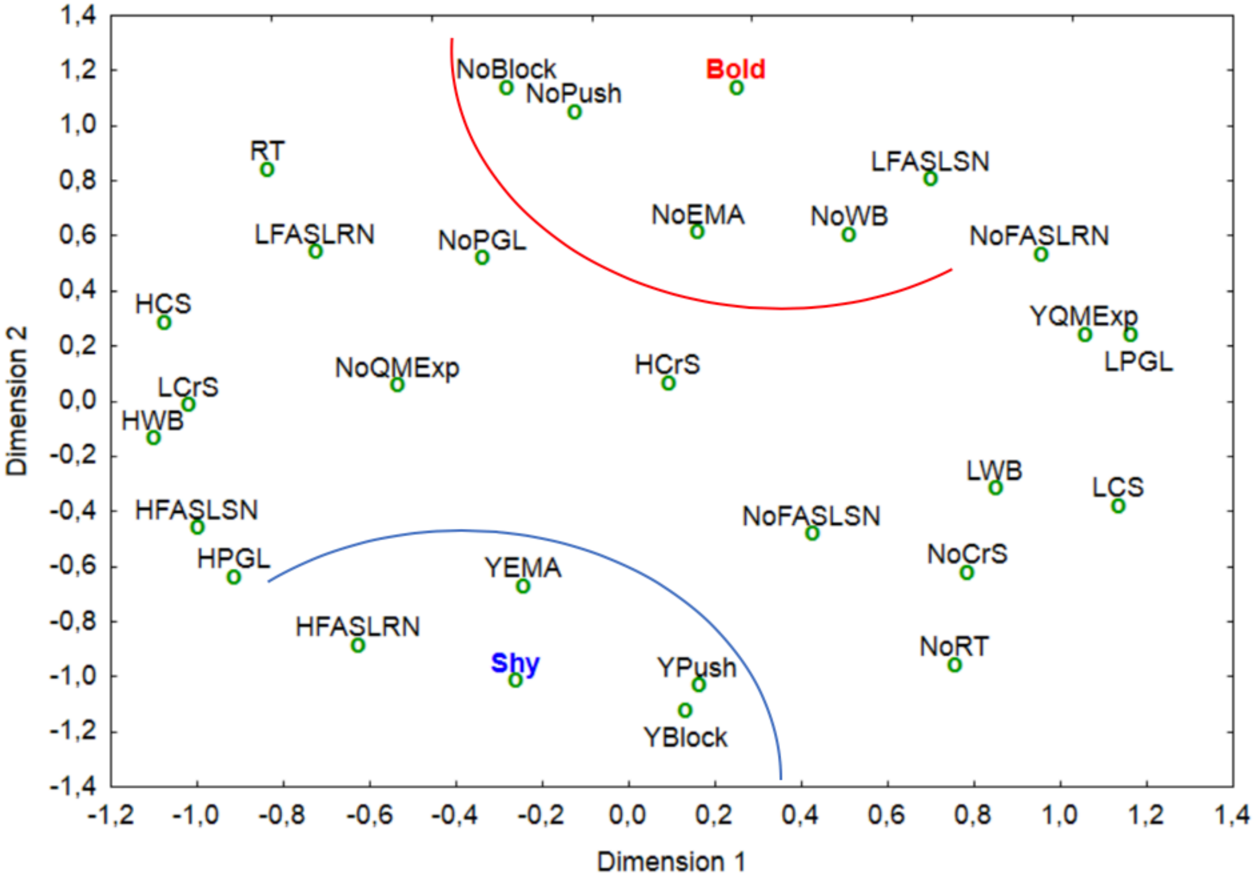

Figure6

Click here to access/download;Figure;Fig 6.pdf

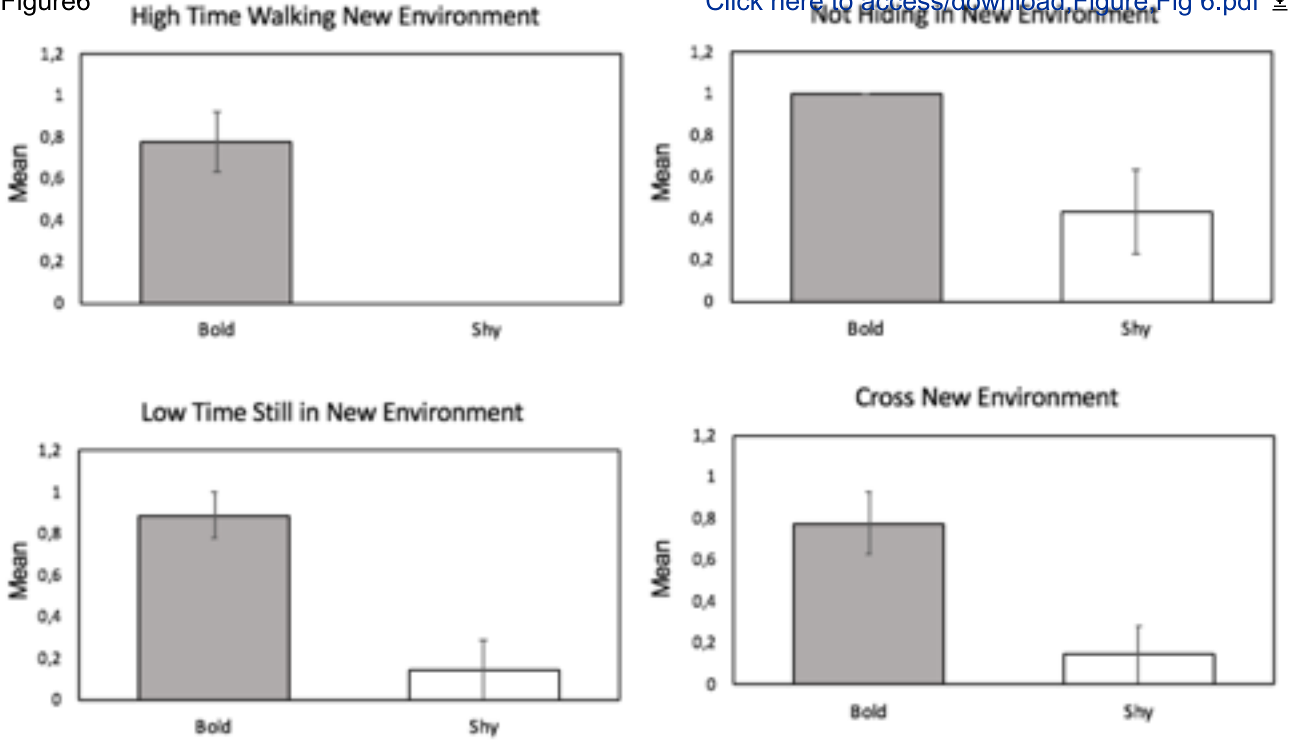

Figure7

Click here to access/download;Figure;Fig 7.pdf

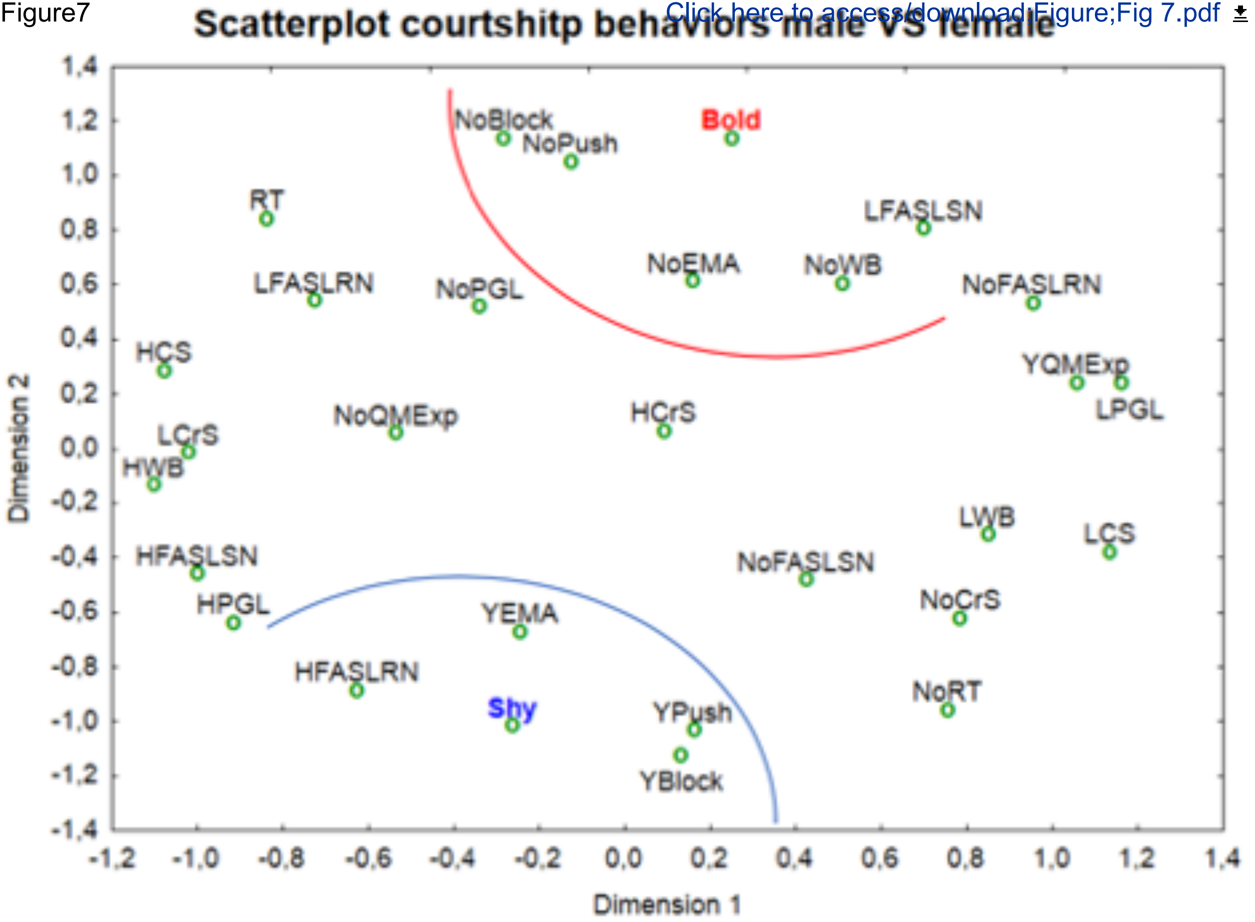

Figure8

Click here to access/download;Figure;Fig 8.pdf

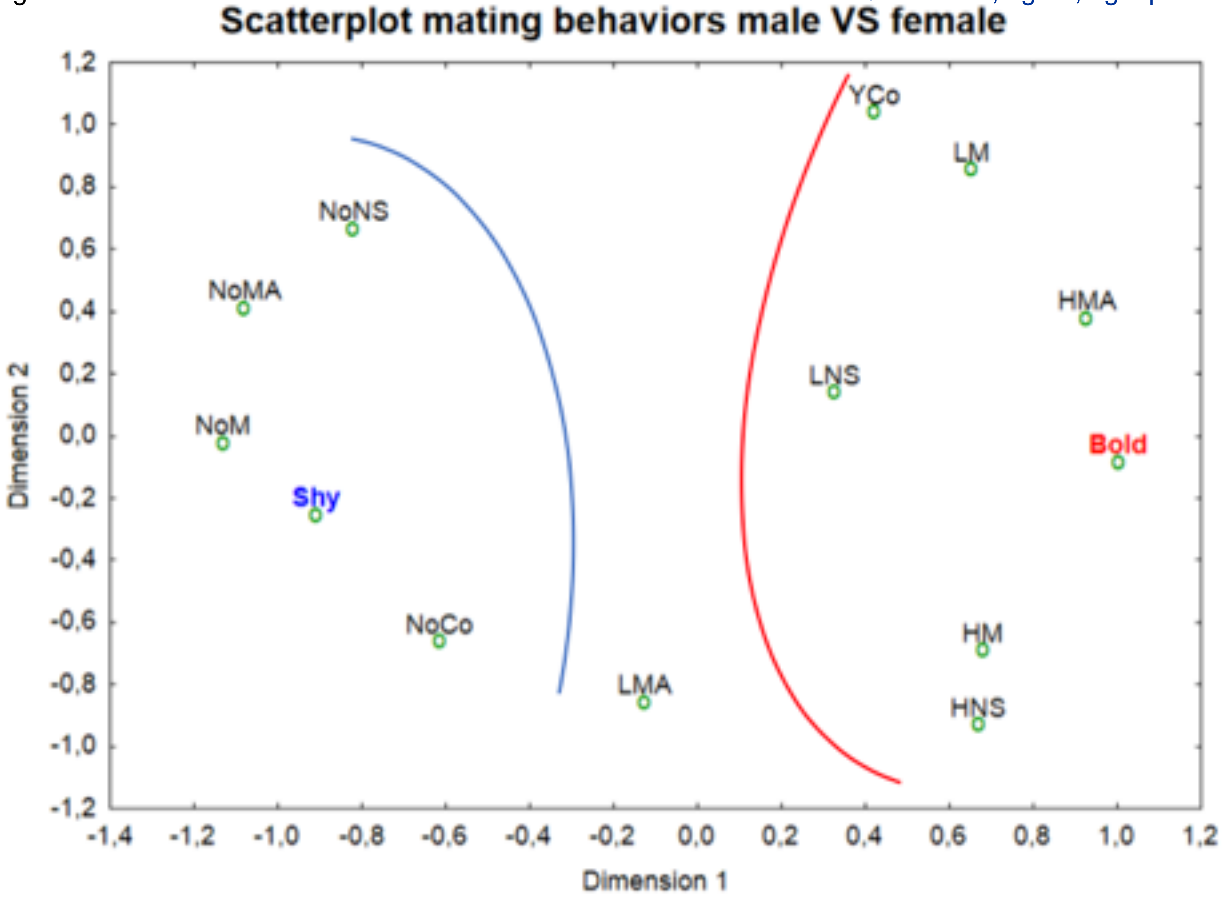

Figure9

Click here to access/download;Figure;Fig. 9.pdf

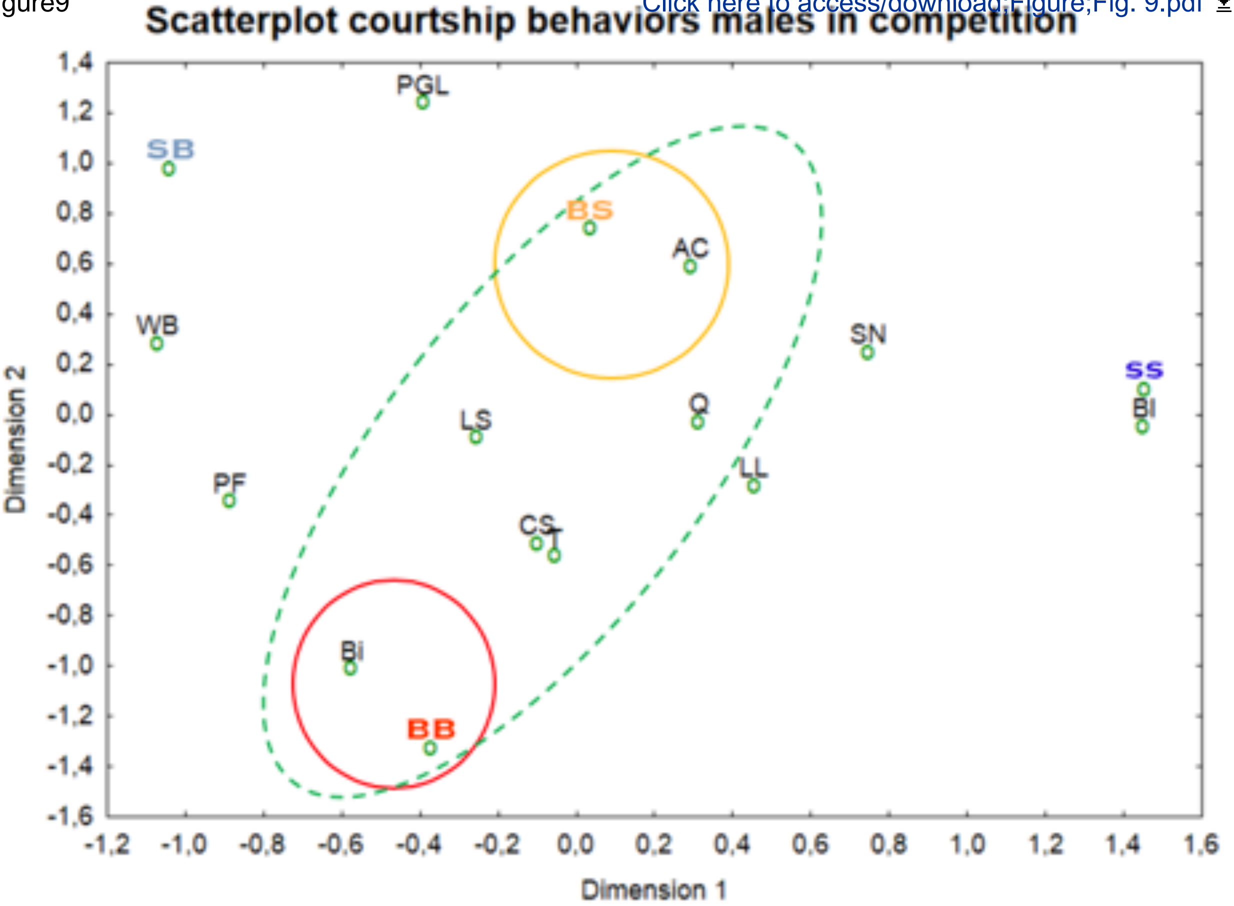

Video1

Click here to access/download
Supporting Information
S1 Video.mp4

Video2

Click here to access/download
Supporting Information
S2 Video.m4v

S1 Table

Click here to access/download
Supporting Information
S1 Table.docx

S2 Table

Click here to access/download
Supporting Information
S2 Table.docx

S3 Table

Click here to access/download
Supporting Information
S3 Table.docx

S4 Table

Click here to access/download
Supporting Information
S4 Table.docx

S5 Table

Click here to access/download
Supporting Information
S5 Table.docx

S6 Table

Click here to access/download
Supporting Information
S6 Table.docx

S7 Table

Click here to access/download
Supporting Information
S7 Table.docx

S8 Table

Click here to access/download
Supporting Information
S8 Table.docx
