# Supplementary material for: Temperament and sexual behaviour in the Furrowed Wood Turtle Rhinoclemmys areolata
Source: PLoS One. 2020 Dec 30;15(12):e0244561. doi: 10.1371/journal.pone.0244561 (PMC7773281; doi:10.1371/journal.pone.0244561)
Supplement: Attachment — Submitted filename: Personalities, Animal Book Review.pdf [file pone.0244561.s012.pdf]

Asterias amurensis, an invasive species introduced into Australia in the 1990s.

Again, however, those expecting the book to provide information on the diversity of asteroids across a broad range will be disappointed, as noticeably prominent groups are absent. For example, the Asterinidae, including *Patiria miniata,* was not included, yet this species has been a workhorse in ecological and developmental studies, and it would have been a good candidate for inclusion. No velatidans, such as the more commonly encountered *Pteraster*, which was studied for its novel developmental and larval biology (e.g., McEdward 1995), were included. Other than *Odontaster*, few other cold-water asteroids, either from high latitudes or the deep sea, were profiled.

Overview

Comprehensive texts that specialize in echinoderms, and especially on asteroids, are few. Available texts are either field guides (e.g., Lambert 2000) or they are out of print (e.g., Clark 1962). Given the widespread importance of asteroids, it seems likely that "Starfish: Biology and Ecology of the Asteroidea" will find its way onto the shelves of many libraries and organizations with invertebrate zoology, marine biology, or other pertinent, comprehensive interests. "Starfish" is a book of uneven coverage likely to find the most use by ecologists, physiologists, and those who work with the living "biology" of asteroids rather than those working in biodiversity or systematics. This book is a good effort and a fine resource for what it is, a current "state of the art" text for specialists with interests in specific topics, but it is not a comprehensive "go to" resource for those seeking general, introductory resources.

Christopher Mah
Research Collaborator, Department of Invertebrate Zoology, MRC-163, PO Box 37012, NMNH. Smithsonian Institution, Washington D.C. 20013
E-mail: mahch@si.edu

Advanced Access publication August 5, 2013
doi:10.1093/icb/ict090

References

Blake DB. 1987. Classification and phylogeny of post-Paleozoic sea stars (Asteroidea: Echinodermata). J Nat Hist 21:481–28.

Clark AM. 1962. Starfishes and related echinoderms. 3rd ed. Hong Kong: T.F.H. Publications.

Jangoux M. 1982. Food and feeding mechanisms: Asteroidea. In: Jangoux M, Lawrence AA, editors. Echinoderm nutrition Rotterdam: Balkema Publishers.

Lambert P. 2000. Sea stars of British Columbia, Southeast Alaska, and Puget Sound. Royal British Columbia Handbook. Vancouver, British Columbia: University of British Columbia Press.

McEdward L. 1995. Evolution of pelagic direct development in the starfish *Pteraster tesselatus* (Asteroidea: Velatida). Biol J Linn Soc 54:299–327.

Sloan NA. 1980. Aspects of the feeding biology of asteroids. Oceanogr Mar Biol Annu Rev 18:57–124.

Spencer WK, Wright CW. 1966. Asterozoans, Part U: Echinodermata. In: Moore RC, editor. Treatise on invertebrate paleontology 3(1): U4-U107. Lawrence: University of Kansas Press.

Zulliger DE, Lessios HA. 2010. Phylogenetic relationships in the genus *Astropecten* Gray (Paxillosida: Astropectinidae) on a global scale: molecular evidence for morphological convergence, species-complexes and possible cryptic speciation. Zootaxa 2504:1–19.

Downloaded from http://icb.oxfordjournals.org/ by guest on May 27, 2014

***Animal Personalities: Behavior, Physiology, and Evolution*. Claudio Carere and Dario Maestripieri, editors.**

The University of Chicago Press, Chicago, IL, 2013, 520 pp. ISBN 978-0-226-92205-8 (hardcover, $110.00) and 978-0-226-92197-6 (paperback, $45.00).

The study of behavior has an interesting history, born of conflict between continents and perspectives and generating separate lineages. Even today these lineages ignore one another, yet occasionally intellectually "raid" the other for ideas that, while popular, yield dubious hybrids. The well-known foray of psychologists into evolutionary biology for explanations of human behavior has yielded a curious product of doubtful legitimacy. Another offensive is at hand. Over the past decade there has emerged like a virus within behavioral ecology the cooption of the term "personality" as a new field of study in animal behavior.

"Personality," a term traditionally reserved for humans to denote individuality and/or the experiences that lead to the development of a person has, until now not been applied to nonhuman animals. In the increasing anthropomorphic spirit evident among younger behavioral biologists (Kennedy 1992), "animal personality" has become defined as the phenotypic characteristics peculiar to individual animals. A prime example of selective scholarship to bolster a spurious scientific agenda is evident when

© The Author 2013. Published by Oxford University Press on behalf of the Society for Integrative and Comparative Biology. All rights reserved. For permissions please email: journals.permissions@oup.com.

the editors state that individual "differences were rarely viewed as an extension of biological meaningful variation" (p. 2). Surely the contributors to this volume know that the idea of individual variation as the basis of evolution existed before Charles Darwin! If the intent of this volume is to promote credibility in order to secure funds to pursue research, then the editors and most of the authors should be honest and straightforward. Nothing attracts curiosity of the uneducated, and disdain by scholars, as such pandering.

Although most of the articles in this volume are by behavioral ecologists, Gosling and Mehta, both psychologists by training, briefly touch on the historical issue before summarily dismissing it. They argue that "individual differences in adult animal behavior are a function of both biological tendencies and experience," with which I agree. To follow this reasoning by stating that since such elements also apply to humans, therefore human terminology can apply to animals, is illogical. Without going into too much historical detail, as it would be invidious and take from the authors' contributions, it is sufficient to forewarn the purveyors of this "new" field to heed the lessons of some of the early psychologists. First and foremost, from C. Lloyd Morgan (1903), the preeminent British psychologist, there is Morgan's Canon "In no case is an animal activity to be interpreted in terms of higher psychological processes if it can be fairly interpreted in terms of processes which stand lower in the scale of psychological evolution and development."(p. 59). This restatement of the principle associated with William of Ockham (14th century) that assumptions introduced to explain a thing must not be multiplied beyond necessity continues to apply. Other early psychologists such as William James and James Baldwin also cautioned strongly against anthropomorphic interpretations. Perhaps a new and unfortunate trend is believed necessary since the type of experiments required for understanding the proximate mechanisms of behavioral development as illuminated by psychobiologists are simply not possible on species living in nature, but the leap of faith that analogy will tell us anything about the mechanisms is unlikely to bring us any closer to a better understanding of animal behavior.

Classical ethologists were concerned with the evolution and ecological significance of behaviors that characterized species, particularly focusing on how species differed in mating and aggression, and developing to concepts such as species-specific behaviors and fixed action patterns. At the same time, comparative psychologists were more interested in the development of such behaviors and their underlying causes. Niko Tinbergen attempted a rapprochement in his classic 1963 article and subsequently both sides softened. In this context, the work of George Barlow emphasizing species-typical behaviors and modal action patterns marked an important advance in the study of individuals versus species. The work of Eckhard Hess and Jack Hailman on how such behaviors might develop from rudimentary elements was another important step. The shift they marked was from an emphasis on mean differences *between* individuals to mean differences of components of behavior *within* individuals. None of this work is mentioned in this volume, presenting instead the idea that the study of individuals is a "new" field. Nor is the idea that clustering behaviors into suites, resulting in terms such as bold, shy, dominant, subordinate, inquisitive, etc., novel or particularly illuminating.

The volume is divided into four sections. The first section (Personalities across Animal Taxa) details studies of invertebrates, sticklebacks, birds, and primates. The first chapter by Mather and Logue dodges the central complaint by this reviewer by sidestepping with the caveat that they use personality as a term "as defined in the introduction." However, put simply, suggesting that insects have personalities diminishes the concept to the extreme. The authors provide a useful compendium of studies of individual differences found with various taxa. The second chapter by Bell, Foster, and Wund is an excellent summary of work on consistent individual differences among sticklebacks according to their respective ecologies, but their initial statements that individual differences present a conundrum to evolutionary biologists and psychobiologists reveals how little they appreciate work of the previous century. The chapter by van Oers and Naguib on avian species does contain a refreshing and thoughtful historical section, but ornithology has always had its legion of anthropomorphizing birders and twitchers. While not all birders/twitchers are ornithologists, I have never met an ornithologist who was not also a birder/twitcher. Weis and Adams present a chapter on primates that is remarkable more for what it leaves out in terms of behavioral ecology than what it says.

The second section (Genetics, Ecology and Evolution of Animal Personalities) is more of the same with a single exception. Sih retains his insistence that individual variation is best described as syndromes. Although suites of behavior is the traditional term, and syndromes is also borrowed from medicine, this term captures better than "personality" that fact that individuals tend to group in

Downloaded from http://icb.oxfordjournals.org/ by guest on May 27, 2014

behavioral types as a consequence of ecological, developmental, and social constraints. Sih, who has been prominent in bringing the issue of behavioral differences into behavioral ecology, thankfully spares the reader in his chapter, opting instead to emphasize the phenomenon of behavioral consequences both within and between species. He stumbles a few times, but immediately corrects himself by declaring his intent is behavioral types. The only other use of personality is a short paragraph on research on mate choice in humans.

The third section (Development of Personalities and their Underlying Mechanisms) has a notable outlier. Curly and Branaki prefer stable individual differences to characterize the phenomenon of interest and present their facts and predictions in a meaningful and thoughtful manner as it relates to the central problem, namely how genes, environment, and epigenetic mechanisms interact to sculpt the adult individual. Particularly interesting is how experiences during sensitive periods of development can alter behavioral and brain phenotypes. Recent research (Freund et al. 2013) shows how individual differences among inbred mice become more pronounced over time in enriched environments. Given that genotype and environment remain constant throughout life in this system, this suggests that individuality is a cumulative process without end.

The final section (Implications of Personality Research for Conservation Biology, Animal Welfare, and Human Health) catapults "animal personality" into broader questions of conservation, aquaculture, and translational applications of rodent research into human health issues. Again, there is a standout chapter (Huntingford, Mesquita, and Kadri) that basically advances the old observations of Marian and Keller Breland of the importance of species differences in predispositions to learn as important considerations for the aquaculture industry.

Let me end by returning to the larger issue and indicating that this new anthropomorphism is unnecessary and should be viewed with skepticism. "Evolutionary Psychology" has become entrenched in psychology, but thankfully not in biology. "Personality" may have the same trajectory, but hopefully biologists will be immune to such infection. This only leaves what can be said positive about the book? It is admirable that most of the authors cite the recent literature (most references are from 1995 to the present), but most of the chapters are without benefit of historical context. By ignoring this history the authors are building an edifice without a foundation.

David Crews
University of Texas at Austin, Austin, TX, USA
E-mail: crews@austin.utexas.edu

Advanced Access publication September 9, 2013
doi:10.1093/icb/ict095

References

Kennedy JS. 1992. The new anthropomorphism. Cambridge: Cambridge University Press.

Freund J, Brandmaier AM, Lewejohann L, Kirste I, Kritzler M, Krüger A, Sachser N, Lindenberger U, Kempermann G. 2013. Emergence of individuality in genetically identical mice. Science 340:756–9.

Morgan CL. 1903. An introduction to comparative psychology. 2nd ed. London: W. Scott. p. 59.

Tinbergen N. 1963. On aims and methods of ethology. Zeitschrift für Tierpsychologie 20:410–33.

Downloaded from http://icb.oxfordjournals.org/ by guest on May 27, 2014
