# Supplementary material for: Temperament and sexual behaviour in the Furrowed Wood Turtle Rhinoclemmys areolata
Source: PLoS One. 2020 Dec 30;15(12):e0244561. doi: 10.1371/journal.pone.0244561 (PMC7773281; doi:10.1371/journal.pone.0244561)
Supplement: Attachment — Submitted filename: DC edits PONE-D-20-14068_R1.pdf [file pone.0244561.s014.pdf]

PLOS ONE

Temperament and sexual behaviour in the Furrowed Wood Turtle Rhinoclemmys areolata

--Manuscript Draft--

**Manuscript Number:**	PONE-D-20-14068R1
**Article Type:**	Research Article
**Full Title:**	Temperament and sexual behaviour in the Furrowed Wood Turtle Rhinoclemmys areolata
**Short Title:**	Temperament and sex in a terrestrial turtle
**Corresponding Author:**	Yann Henaut, Ph.D.<br>El Colegio de la Frontera Sur<br>Chetumal, Quintana Roo MEXICO
**Keywords:**	Temperament; agonistic behavior; reproduction; terrestrial turtle
**Abstract:**	Temperament variability in animals have consequences for evolution and ecology. One of the primary effects of consistent behavioral differences points to reproduction. In chelonians some works have focused on the study of temperament using different methods for its measurement. In our research we first wanted to i) establish a methodology to determine boldness degree, in the turtle Rhinoclemmys areolata . Secondly, we wanted to ii) determine the role boldness plays during reproduction, with emphasis on courtship and copula, considering a) the interactions between male and female, and b) the competition between males. We used 16 sexually mature individuals of each sex. We first observed the males in four different situations and recorded 17 behavioral traits. We selected 12 traits that allowed us to determine the bolder and the shier individuals. We found that five behavioral traits were specific for bolder individuals and five others for the shier. In a second step, we observed a male in presence of a female and recorded courtship behaviors and breeding attempts. Bolder individuals did not display courtship behaviors and just attempt to copulate. Shier individuals displayed courtship behaviors and rare copula attempts. Finally, in the simulations that compared two males in the presence of a female we noticed that bolder individuals showed courtship behaviors while the more shy ones simply ignored the female. Our results first allowed us to determine which methodology is the best to determine temperament in turtles. Secondly, the temperament seems to be an important factor to modulate interaction between males and females. Bolder individuals have an advantage during competition and display courtship behaviours only if other males are present. Shier males displayed courtship behaviors and try to copulate only when no competitors were present. Those two different temperament-dependant strategies are discussed in terms of ecology, evolution and management.
**Order of Authors:**	Francesca Maura Cassola
	Yann Henaut, Ph.D.
	José Rogelio Cedeño-Vázquez
	Fausto Roberto Méndez-de la Cruz
	Benjamín Morales-Vela
**Response to Reviewers:**	A)Editor comments (changes in green in the document)<br>1)We revised Plos One style requirements.<br>2)To answer the editor comments about ethical statements (points 2, 3, 4, 5, 6), in the ethical declaration we added the following paragraph:<br>“All the animals come from private loans of the city of Chetumal (Quintana Roo – Mexico). Verbal consent was requested from the owners, and it was explained how the turtles would be used and for what objective. The individuals on loan were destined exclusively for specific research purposes, respecting all known needs for animal welfare, and were returned to their owners at the end of the data collection.<br>We were in possession of the permit N ° SGPA / DGVS / 002491/18 issued by Secretaria de Medio Ambiente y Recursos Naturales (SEMARNAT) for the collection of this species.”

Powered by Editorial Manager® and ProduXion Manager® from Aries Systems Corporation

3)Page 18 line 325: in Materials and Methods we corrected the mistake in the table caption, it was a typing error.
4)Pages 30-32 lines 581-620: We added the captions for supporting information files.
5)We revised all the references and updated all the new citations.

B)Responses to Reviewer (in yellow in the document):
Short Title
6)Page 1 Line 10: we deleted the short tittle after we revised Plos One style requirements (see Editor comments).

Summary
7)Page 2 Lines 29-31: According to the reviewer comment, we changed the phrase "Finally, we observed two males in presence of one female and noticed that bolder individuals then displayed courtship behaviors while the shier just ignore the female." with "Finally, in the simulations that compared two males in the presence of a female we noticed that bolder individuals showed courtship behaviors while the more shy ones simply ignored the female." The change was made because the result does not come from a single anecdote as the previous sentence suggested, but from multiple individuals and repetitions.
8)Page 2 Lines 34-35: following the previous correction this sentence should now coincide with results from multiple experiments and not just one, as it previously seemed.
9)Page 2 line 48: we deleted the Key Word after we revised Plos One style requirements (see Editor comments).

Introduction
10) Page 3 line 44-45: as suggested by the reviewer we suppressed the term personality considered as anthropomorphic and controversial. Also, as suggested, we mentioned now the term "reactivity" and suppressed "permanently".
11) Page 3 line 54: we changed "breed" with "breeding" as suggested by the reviewer.
12) Page 3 line 56: as suggested by the reviewer we added the citation "Gowaty PA, Drickamer LC, Schmid-Holmes S. Male house mice produce fewer offspring with lower viability and poorer performance when mated with females they do not prefer. Anim. Behav. 2003;65: 95-103".
13) Page 3 line 59: as suggested by the reviewer we added the citation "Ah-King, M., & Gowaty, P. A. (2016). A conceptual review of mate choice: stochastic demography, within-sex phenotypic plasticity, and individual flexibility. Ecology and evolution, 6(14), 4607-4642".
14) Page 4 line 69: as suggested by the reviewer, we added "giving primary importance to survival over reproductive productivity" to specify behaviors.
15) Page 4-5 lines 85-90: as suggested by the reviewer we suppressed the unnecessary sentence and add references (Beach FA and Leboeuf BJ 1967, Coital behaviour in dogs. I. Preferential mating in the bitch. Animal behaviour, 15(4) 546-548, Sánchez-Macouzet O, Rodríguez C, Drummond H. 2014 Better stay together: pair bond duration increases individual fitness independent of age-related variation. Proc. R. Soc. B 281: 20132843).
16) Page 4-5 lines 85-90: As suggested by the reviewer in the discussion, we added the following paragraph in the introduction "There are, for example, cases where behavioral similarity is the key to reproductive success, as in the case of zebra finches (Taeniopygia guttata) and cockatiels (Nymphicus hollandicus) [31-32] ; while, in other species, different or even opposite temperaments can reach high reproductive performances as in the giant panda (Ailuropoda melanoleuca) and in the black rhino (Diceros bocornis) [12, 25, 33]."
17) Page 5 line 91: we created a new sentence as suggested by the reviewer deleting "and the study shows that"
18) Page 5 line 98: as suggested by the reviewer we changed boldness for reactivity.
19) Page 5 line 105: as suggested by the reviewer we changed boldness for reactivity.
20) Pace 5 line 110: we changed "attempt to create" with "creating" as suggested by the reviewer.

Material and Methods
21) Page 6 line 125: we deleted the hyphenation in "North-western".
22) Page 7 line 146: we change the tense of "avoid" to "avoided".

Powered by Editorial Manager® and ProduXion Manager® from Aries Systems Corporation

	23) Page 7 line 147: we were talking about sperm storage that was studied in different turtles' species but there aren't any available work about this species. For a better understanding we changed the word "recollecting" with "storing".<br>24) Page 8 line 167: during the trial we used water only to spraying the individuals, no pool or drinking point were used, therefore there was no reason to refill something. The water we used to spray was always new and after every trial we changed the position of the observation area into a dry one.<br>25) Page 10 line 228: we changed the tense from "will not be" to "were".<br>26) Page 10 line 232: We used "sequentially" to explain that the experiments were done one after the other, but the term is maybe not adequate. We deleted this word to avoid confusion.<br>Results<br>27) Page 12 line 262: we changed the word "organisms" with "turtles" as suggested by the reviewer.<br>28) Page 12 line 265: we changed "didn't" with "did not" as suggested by the reviewer.<br>29) Page 12 line 273: as suggested by the reviewer we change "for the shier" by "characteristic of the shier turtles".<br>30) Page 12 line 276: As suggested by the reviewer we changed "for the boldest" by ". Variables for the boldest turtles were".<br><br>Discussion<br>31) Page 20 line 361: we changed the beginning of the sentence from "Also, our" to "Our" as suggested by the reviewer.<br>32) Page 21 line 386: we deleted "the".<br>33) Page 21 line 386: we added "animals".<br>34) Page 21 line 393-394: according to referee's commentary, we add in the sentence that it was observed for other species "In other species, male competition could be very costly".<br>35) Page 22 lines 407-408: in order to rewrite the sentence as suggested by the reviewer, we changed "respond" with "adapt" and we delete "pass under the clutches of" to add "survive to".<br>36) Page 22 line 418: according to the reviewer comment we suppressed" behavioral trait" and added "temperament" to be more specific in our affirmation.<br>37) Page 22: we deleted the paragraph "In the study of Spoon et al (2006), it can be seen that compatible pairs (male-female) of the Cockatiel Nymphicus hollandicus showed higher levels of coordination in parental care and raised more chicks; another example is given by Carlstead et al (1999a, b) who observed in Black Rhino Diceros bocornis that smaller enclosures determined the breeding of dominant males, while domination decreased in males housed in larger enclosures. In addition, dominance levels were related to reproductive success, with the conclusion that the number of births per year was higher in pairs of rhinos composed of a submissive male and a dominant or aggressive female. This information gives an idea of the optimal conditions for the breeding of captive rhinos, including large enclosures, which facilitate submissive behavior in males, which in turn affects the compatibility of couples; in the giant panda Ailuropoda melanoleuca, Martin-Wintle et al (2017) conducted behavioral studies to further improve reproductive performance and found that specific combinations of temperament traits showed better reproductive performance than others. Males who were more aggressive than their partners were more likely to mate and produce puppies than when the female had a higher level of aggression than the male or even couples with less fearful males, regardless of the fear of the female, obtained better reproductive results. The application of these results to captive breeding management strategies should result in higher reproductive rates and the production of more candidates for the giant panda reintroduction program." and added a resumed version in page 4 lines 95-100.<br>38) Page 22 lines 428: we deleted "focused on boldness degree of males", same with "therefore they could have a" and added "with perhaps".<br>39) Page 23 lines 435 and 437: we deleted "has allowed to understand" and added "demonstrates" and added "animals".<br>40) Page 23: as suggested by the reviewer and to avoid repetition of the same idea, we deleted the sentence "By another way in term of evolution, being shiest may have some advantages and, if not for reproduction, they may be for antipredatory behaviour, making shiest individuals, that are more prudent in term of exploration, and as we observed in the new environment, generally less predated than boldest individuals as discussed for other species."

Powered by Editorial Manager® and ProduXion Manager® from Aries Systems Corporation

	41) Page 24: as suggested by the reviewer we deleted the last paragraph.<br><br><br>Bibliography<br>42) Following the suggestions of the reviewer we made changes in the citations. We added the following works:<br>-Gowaty PA, Drickamer LC, Schmid-Holmes S. Male house mice produce fewer offspring with lower viability and poorer performance when mated with females they do not prefer. Anim. Behav. 2003;65: 95-103.<br>-Ah-King M, Gowaty PA. A conceptual review of mate choice: stochastic demography, within-sex phenotypic plasticity, and individual flexibility. Ecol. Evol. 2016;6: 4607-4642.<br>-Beach FA and Leboeuf BJ Coital behaviour in dogs. I. Preferential mating in the bitch. Anim Behav. 1967; 15: 546-548.<br>-Sánchez-Macouzet O, Rodríguez C, Drummond H. Better stay together: pair bond duration increases individual fitness independent of age-related variation. Proc. R. Soc. B. 2014; 281: 20132843.<br>And deleted:<br>-Cable RN. Oh, Behave: When wildlife behavior matters in Conservation. Mich. J. Sustain. 2013;1<br>-Cooper Jr WE. Variation in escape behavior among individuals of the striped plateau lizard Sceloporus virgatus may reflect differences in boldness. J. Herpetol. 2009;43:495-502.
**Additional Information:**	
**Question**	**Response**
**Financial Disclosure**<br><br>Enter a financial disclosure statement that describes the sources of funding for the work included in this submission. Review the submission guidelines for detailed requirements. View published research articles from *PLOS ONE* for specific examples.<br><br>This statement is required for submission and **will appear in the published article** if the submission is accepted. Please make sure it is accurate.	CFM and CVJR. CONACYT-OEA-AMEXCID scholarship program for the funds needed to cover expenses for data collection and specimen maintenance.

**Unfunded studies**<br>Enter: *The author(s) received no specific funding for this work.*<br><br>**Funded studies**<br>Enter a statement with the following details:<br>• Initials of the authors who received each award<br>• Grant numbers awarded to each author<br>• The full name of each funder<br>• URL of each funder website<br>• Did the sponsors or funders play any role in the study design, data collection and analysis, decision to publish, or preparation of the manuscript?<br>• **NO** - Include this sentence at the end of your statement: *The funders had no role in study design, data collection and analysis, decision to publish, or preparation of the manuscript.*<br>• **YES** - Specify the role(s) played.<br><br>* typeset	
**Competing Interests**<br><br>Use the instructions below to enter a competing interest statement for this submission. On behalf of all authors, disclose any competing interests that could be perceived to bias this work—acknowledging all financial support and any other relevant financial or non-financial competing interests.<br><br>This statement **will appear in the published article** if the submission is accepted. Please make sure it is accurate. View published research articles from *PLOS ONE* for specific examples.	The authors have declared that no competing interests exist.

**NO authors have competing interests**<br><br>Enter: *The authors have declared that no competing interests exist.*<br><br>**Authors with competing interests**<br><br>Enter competing interest details beginning with this statement:<br><br>*I have read the journal's policy and the authors of this manuscript have the following competing interests: [insert competing interests here]*<br><br>* typeset	
**Ethics Statement**<br><br>Enter an ethics statement for this submission. This statement is required if the study involved:<br><br>• Human participants<br>• Human specimens or tissue<br>• Vertebrate animals or cephalopods<br>• Vertebrate embryos or tissues<br>• Field research<br><br>Write "N/A" if the submission does not require an ethics statement.<br><br>General guidance is provided below. Consult the submission guidelines for detailed instructions. **Make sure that all information entered here is included in the Methods section of the manuscript.**	The investigation was carried out in compliance with the institutional ethical standards and norms in force. Experimental protocol was approved by Ethical Committee from the “El Colegio de la Frontera Sur”, Mexico. All the animals come from private loans of the city of Chetumal (Quintana Roo – Mexico). Verbal consent was requested from the owners, and it was explained how the turtles would be used and for what objective. The individuals on loan were destined exclusively for specific research purposes, respecting all known needs for animal welfare, and were returned to their owners at the end of the data collection. We were in possession of the permit N ° SGPA / DGVS / 002491/18 issued by Secretaria de Medio Ambiente y Recursos Naturales (SEMARNAT) for the collection of this species.

**Format for specific study types**<br><br>**Human Subject Research (involving human participants and/or tissue)**<br>• Give the name of the institutional review board or ethics committee that approved the study<br>• Include the approval number and/or a statement indicating approval of this research<br>• Indicate the form of consent obtained (written/oral) or the reason that consent was not obtained (e.g. the data were analyzed anonymously)<br><br>**Animal Research (involving vertebrate animals, embryos or tissues)**<br>• Provide the name of the Institutional Animal Care and Use Committee (IACUC) or other relevant ethics board that reviewed the study protocol, and indicate whether they approved this research or granted a formal waiver of ethical approval<br>• Include an approval number if one was obtained<br>• If the study involved *non-human primates*, add *additional details* about animal welfare and steps taken to ameliorate suffering<br>• If anesthesia, euthanasia, or any kind of animal sacrifice is part of the study, include briefly which substances and/or methods were applied<br><br>**Field Research**<br><br>Include the following details if this study involves the collection of plant, animal, or other materials from a natural setting:<br>• Field permit number<br>• Name of the institution or relevant body that granted permission	
**Data Availability**<br><br>Authors are required to make all data underlying the findings described fully available, without restriction, and from the time of publication. PLOS allows rare exceptions to address legal and ethical concerns. See the PLOS Data Policy and FAQ for detailed information.	Yes - all data are fully available without restriction

A Data Availability Statement describing where the data can be found is required at submission. Your answers to this question constitute the Data Availability Statement and **will be published in the article**, if accepted.<br><br>**Important:** Stating 'data available on request from the author' is not sufficient. If your data are only available upon request, select 'No' for the first question and explain your exceptional situation in the text box.<br><br>Do the authors confirm that all data underlying the findings described in their manuscript are fully available without restriction?	
**Describe where the data may be found in full sentences. If you are copying our sample text, replace any instances of XXX with the appropriate details.**<br><br>• If the data are **held or will be held in a public repository**, include URLs, accession numbers or DOIs. If this information will only be available after acceptance, indicate this by ticking the box below. For example: *All XXX files are available from the XXX database (accession number(s) XXX, XXX.*).<br>• If the data are all contained **within the manuscript and/or Supporting Information files**, enter the following: *All relevant data are within the manuscript and its Supporting Information files.*<br>• If neither of these applies but you are able to provide **details of access elsewhere**, with or without limitations, please do so. For example:<br><br>*Data cannot be shared publicly because of [XXX]. Data are available from the XXX Institutional Data Access / Ethics Committee (contact via XXX) for researchers who meet the criteria for access to confidential data.*<br><br>*The data underlying the results presented in the study are available from (include the name of the third party*	All relevant data are within the manuscript and its Supporting Information files.

and contact information or URL).<br>• This text is appropriate if the data are owned by a third party and authors do not have permission to share the data.<br><br>* typeset	
Additional data availability information:	

Powered by Editorial Manager® and ProduXion Manager® from Aries Systems Corporation

Manuscript Click here to access/download;Manuscript;Manuscript.docx

Temperament and sexual behaviour in the Furrowed Wood Turtle *Rhinoclemmys areolata*

Cassola FM [1], Henaut Y [1*], Cedeño-Vázquez JR [1], Méndez-de la Cruz FR[2], Morales-Vela B[1]

[1]El Colegio de la Frontera Sur, Unidad Chetumal, Chetumal, Quintana Roo, México

[2]Laboratorio de Herpetología, Departamento de Zoología, Instituto de Biología, Universidad Nacional Autónoma de México. México.

*Corresponding Author

e-mail: yhenaut@ecosur.mx

Abstract

The variation in temperament among animals has consequences for evolution and ecology. One of the primary effects of consistent behavioral differences points to reproduction. In chelonians some works have focused on the study of temperament using different methods for its measurement. In our research we first wanted to i) establish a methodology to determine boldness degree, in the turtle *Rhinoclemmys areolata*. Secondly, we wanted to ii) determine the role boldness plays during reproduction, with emphasis on courtship and copula, considering a) the interactions between male and female, and b) the competition between males. We used 16 sexually mature individuals of each sex. We first observed the males in four different situations and recorded 17 behavioral traits. We selected 12 traits that allowed us to determine the bolder and the shier individuals. We found that five behavioral traits were specific for bolder individuals and five others for the shier. In a second step, we observed a male in presence of a female and recorded courtship behaviors and breeding attempts. Bolder individuals did not display courtship behaviors and just attempt to copulate. Shier individuals displayed courtship behaviors and rare copula attempts. Finally, in the simulations that compared two males in the presence of a female we noticed that bolder individuals showed courtship behaviors while the more shy ones simply ignored the female. Our results first allowed us to determine which methodology is the best to determine temperament in turtles. Secondly, the temperament seems to be an important factor to modulate interaction between males and females. Bolder individuals have an advantage during competition and display courtship behaviours only if other males are present. Shier males displayed courtship behaviors and try to copulate only when no competitors were present. Those two different temperament-dependant strategies are discussed in terms of ecology, evolution and management.

Introduction

Inter-individual behavioral differences in an animal species are consistent or largely maintained over time and in diverse situations [1] and sometimes between one population and the other [2]. These differences are what some scientists call “temperament”, “behavioral syndrome” or “reactivity”[3-4] and they have been attributed to the combined influences of genetic, social and environmental factors that affect the behavior of an individual [5].

Animal behavioral differences can have important implications for fitness [6-7] and have been shown to influence ecological processes such as dispersion, invasion, response to climate change and risk of extinction [8-10]. There is a high probability that temperament has a great influence on the way in which animals respond to new environments (for example, new exhibits or retention areas), to familiar and unfamiliar conspecifics, to strangers of other species, and to changes in their environment, affecting social compatibility, stability and success of a group [11]. Animal behavior and temperament can influence breeding in capacity by directly connecting to a successful match (success of coupling) or the number and viability of offspring produced (reproductive success) by the specimens [12-14]. Since temperament affects the way in which individuals react to difficult situations, it can be predicted how a male can compete, select and be compatible with a sexual partner. Therefore, variations in individual behavior are a source of information in the field of sexual selection and reproductive success [6,15].

Individuals may systematically differ in one or more temperament traits, such as boldness, aggressiveness, reactivity, sociability, exploration or activity levels [1]. A feature that has been extensively studied in the field of temperament is boldness defined as the inclination of individuals to explore their environment and take risks in a new situation [16]. Boldness

and shyness are considered as a continuum [17-18] at one extreme we find shier specimens and on the opposite side the bolder ones.

This spectrum ~~seems to have~~ an adaptive explanation in terms of costs and benefits, related to the type of response to a risk. In general, bolder individuals tend to sustain high reproductive success but at a potential cost for their survival, while shy individuals engage in opposite behaviors, giving primary importance to survival over reproductive productivity. This implies that temperament would allow to regulate survival with other needs of the species [17].

There are few studies addressing the relationship between success of couples and behavioral compatibility. Knowing these individual differences provides essential information for captivity management and animal welfare [11,17]. For example, it allows us to understand why some specimens have reproductive problems in captivity and why they fail to satisfy their reproductive potential, even if they are physically and physiologically in health [19]. A few years ago, in fact, specimens suitable for mating were chosen based on their hypothetical genetic compatibility [20-21], their state of health [22] and / or stages of life history [23-24]. However, couples who represent a good genetic combination do not necessarily produce offspring, and behavioral incompatibility is often cited for this failure [25-28]. One way in which animal temperament can influence mating probability is through the effects associated with behavioral compatibility the way individuals interact with each other [12]. When animals are paired for reproduction, some temperaments may be compatible, while others may be in conflict [13,29-30]. There are, for example, cases where behavioral similarity is the key to reproductive success, as in the case of zebra finches (*Taeniopygia guttata*) and cockatiels (*Nymphicus hollandicus*) [31-32] ; while, in other species, different or even opposite temperaments can reach high reproductive

performances as in the giant panda (*Ailuropoda melanoleuca)* and in the black rhino (*Diceros bocornis)* [12,25,33].

Recently, boldness has been studied in aquatic turtles. The recognition between males and the reduction of competitive interactions are related to the boldness of males [34]. They showed that the response to chemical stimuli of known or unknown males of the Mediterranian Turtle *Mauremys leprosa*, depends on the level of boldness, in particular the shiest males are those who avoid encountering other males, of the same or different species, as it could help stabilize social systems and reduce the frequency and intensity of aggressive encounters between males. Different methods are used to evaluate temperament in turtles. Reactivity level is measured using the straightening response, which is the time it takes for an individual to straighten up after being turned upside down on his carapace [35]. A similar method has been used to study anti-predator responses in the European pond turtle *Emys orbicularis* [36]. Another method consists to insert specimens in a new environment: here, the latency to move from an initial position in an arena was used to evaluate exploration in the red-eared slider turtle *Trachemys scripta* [37] and in the Eastern hermann *Eurotestudo boettgeri* [38]. The presentation of a threatening stimuli was also used to measure the reactivity and the effect of new objects on investigative behaviors to measure exploration in Agassiz´s desert tortoise (*Gopherus agassizii*) [14]. The boldness in the Eastern box turtle (*Terrapene carolina*) was evaluated using the time to emerge from the shell and the time to move after a short period of confinement [39]. Therefore, there are different methods applicable to turtles but none of the aforementioned studies tried to apply more than one of them to a single species. For this reason, creating a unique methodology for the study of boldness in turtles is still lacking.

Unfortunately, there is a lack of work addressing specifically how temperament may be related to the reproductive behavior of turtles, particularly if it influences male competition

behavior, courtship and coupling. Thus, the aims of this study were to (i) determinate temperament of *Rhinoclemmys areolata* and provide a method to determine the temperament in these species that can also be used for others terrestrial turtle species (ii) understand if its temperament could be a factor that may modulate the interaction between male and female, and (iii) between males during the competition for a female.

Materials and methods

Ethical statement

The investigation was carried out in compliance with the institutional ethical standards and norms in force. All the animals come from private loans of the city of Chetumal (Quintana Roo – Mexico). Verbal consent was requested from the owners, and it was explained how the turtles would be used and for what objective. The individuals on loan were destined exclusively for specific research purposes, respecting all known needs for animal welfare, and were returned to their owners at the end of the data collection.

We were in possession of the permit N ° SGPA / DGVS / 002491/18 issued by Secretaria de Medio Ambiente y Recursos Naturales (SEMARNAT) for the collection of this species. Experimental protocol was approved by Ethical Committee from the “El Colegio de la Frontera Sur”, Mexico.

Object of study

For the experiment, we used 16 males and 16 females of the furrowed wood turtle *Rhinoclemmys areolata*, all sexually mature. This species occurs in Yucatán Peninsula, Cozumel Island, Northern Guatemala, Belize, Northwestern Honduras, where it inhabits savannah, thorny scrub, broad-leaved forests, swampy and gouache areas [40]. It is used

as pet, food and in the traditional medicine [41]. It is listed as Near-threatened by the IUCN Red List [42]. It is also known as “Chak pool” or “Mojina” [41]. It is characterized by 1 to 2 red, orange or yellow stripes on the top of the head, and the plastron is yellow with a dark spot of variable size (Fig 1). It has a low level of sexual dimorphism, males having a longer and thinner tail than females, and also having a slightly more concave plastron but this is a characteristic that is not always detectable (Fig 2). It is a terrestrial species, whose only connection with the aquatic environment is during courtship and copulation; however, these derive from two anecdotic observations events where the specimens were kept in pools without the presence of a large terrestrial environment [43-44]. Courtship and copulation could be induced by spraying them with water [40].

Fig 1. Details of *Rhinoclemmys areolata* morphology.

Fig 2. Detail of female (left) and male (right). It can be seen the difference between the tails (red arrow), females have a shorter and thicker tail, while males have a longer and thinner one.

Turtles’ management and setup

Specimens of *Rhinoclemmys areolata* were kept within a 16 x 5 m enclosure, located in the facilities of El Colegio de la Frontera del Sur (ECOSUR) Unidad Chetumal (18 ° 32'37.4''N, 88 ° 15 ' 48.0''W). The area was fenced and divided into two parts of approximately the same size (5 x 8 m) containing male and female specimens, respectively. This division avoided any reproductive activity in the absence of the observer. It also avoids female from storing semen from males and be less reactive to males [45].

The area inside the fence was as close as possible to the natural habitat of *R. areolata*, and it was possible by maintaining the presence of trees, foliage, natural soil and adding water sites (Fig 3).

Turtles were fed with fruit, vegetables and meat on alternate days. Inside the maintenance area, they were free to move, to find refuge and bathe in small pools. They were subject to natural temperatures and rainfall for as long as they were inside the area in order to keep the best environmental conditions for their welfare.

All simulations were performed in a mobile enclosed area (2 x 2 m) (Fig 4). Each simulation was recorded through two cameras (YiLiteCam and Motorola Camera). The first video camera was positioned above the observation area while the second was moved manually by the observer in case details of the events were needed.

Each simulation took place in open air, on sunny days, during hours of maximum activity of turtles (8.30am-11.30am and 05.30pm - 06.30pm), when the temperatures are not very high. Animals were separated after each observation session to avoid any additional intersexual interactions in absence of the observer. The observation area was moved in an adjacent area after every simulation to reduce waiting time between experiments and recovery time of environmental conditions, then a new group of turtles was placed to repeat simulation.

Fig 3. Captive living area of *Rhinoclemmys areolate* at the ECOSUR campus.

Fig 4. Observation area from the side and from the top views.

Experiments

Determination of temperament of *Rhinoclemmys areolata*

It was necessary to identify stable behavioral traits over time and in different situations. For this reason, 16 males were observed individually and in isolation in four different situations:

- Reaction to manipulation: a male is taken from the confinement and handled briefly for 1 minute.
- Reaction to risk of predation: a male is taken from the confinement, handled briefly and gently released in an overturned position, that is, on his carapace.
- Reaction to a new environment: a male is taken from the confinement and released in experimental area unknown to the specimen. (30min)
- Reaction to the introduction of a new object: a male is taken from the confinement, released in experimental area and, after 5 minutes, an unknown object is suddenly released (stuffed dog-like animal). (30min)

To organize males in order to describe their boldness-shyness level we: (i) extrapolated behavioral events of each simulation from video recordings, (ii) converted them into variables, (iii) defined occurrence (=1) and absence (=0) of each event obtaining binomial values; (iv) defined frequency and (v) time in seconds when possible, obtaining quantitative values; (vi) for the latter, it was measured the moment of abrupt change of slope within data range, this way it was possible to classify male as shy or bold (S1 Table).

Each event was considered as a variable and inserted into an Excel sheet. A boldness index was given to each male, dividing the sum of “occurrences” of variables that could describe a bold temperament by the total number of variables. This analysis allowed us to classify specimens into groups, according to their degree of boldness.

Afterwards, variables were analysed using a correlation matrix and the obtained values were inserted into a multidimensional scaling (MDS) that allowed us to elaborate a map showing relative positions between different events and relative distances.

Finally, variables were analysed through a Mann-Whitney test to determine which ones were significant in determining temperament.

Before starting interaction experiments, we tested the influence of turtles' length (that is considered linked with the age of each individual) on temperament. We did this test to ensure results are not the consequence of the size or age of the turtles. The two groups obtained were analysed in relation to straight carapace length (SCL) and straight plastron length (SPL) through Mann-Whitney test (S2 Table).

Temperament as a modulating factor during the interaction between male and female

16 groups of turtles consisting of one male and one female were created randomly. Each couple was released, sequentially, within the experimental area, each individual inside a metal box. They were soaked with water using an artificial sprayer to promote reproductive behavior and later freed to move around for 30 minutes.

For this experiment, the same specimens were not used more than once.

Each group remained sequentially in the experimental arena and was carefully observed.

As for the first simulations, we (i) extrapolated behavioral events from each simulation, (ii) converted them into variables, (iii) defined occurrence (=1) and absence (=0) of each event obtaining binomial values; (iv) defined frequency and (v) time in seconds when possible, obtaining quantitative values; (vi) for the latter, it was measured the moment of abrupt change of slope within data range (S3 and S4 Tables).

Each event was considered as a variable and inserted into an Excel sheet. Variables were analysed using a correlation matrix and the obtained values were inserted into a multidimensional scaling (MDS) that allowed us to elaborate a map showing relative positions between different events and relative distances.

Temperament as a modulating factor during the competitive interaction between males in the presence of a female

Eight experimental groups were randomly created, each consisting of two males and one female. For this experiment, the same specimens were not used more than once.

The specimens were placed equidistant within the experimental area, soaked with water using an artificial sprayer to promote reproductive behavior and freed to move around for 30 minutes.

Each group remained in the experimental area and was carefully observed.

As for the first simulations, we (i) extrapolated behavioral events from each simulation, (ii) converted them into variables, (iii) defined occurrence (=1) and absence (=0) of each event obtaining binomial values; (iv) defined frequency and (v) time in seconds when possible, obtaining quantitative values; (vi) for the latter, it was measured the moment of abrupt change of slope within data range (S5 and S6 Table).

Each event was considered as a variable and inserted into an Excel sheet. Variables were analysed using a correlation matrix and the obtained values were inserted into a multidimensional scaling (MDS) that allowed us to elaborate a map showing relative positions between different events and relative distances.

Results

Determination of temperament of *Rhinoclemmys areolata*

We observed 17 behavioral traits (S1 Table) but several of them were displayed by all the turtles and were not useful to distinguish temperaments; therefore, we selected 12 behavioral traits that were used as variables (Table 1). For each male, the presence or

absence of each of them was defined (Table 2). For this study, we used “bolder” and “shier” terms instead of “bold” and “shy” since boldness is a continuous spectrum and it is not possible to create a sharp and fixed fractures for group definition.

In order to create two groups for the following experiments, we considered as shier all turtles that had an index less than or equal to 0.5, while bolder were those that had an index greater than 0.5. This allowed us to divide specimens into 9 bolder and 7 shier males (Table 2).

Straight carapace length and straight plastron length did not show a significant relationship with temperament (Carapace: Mann-Whitney U test: U=20, P=0.25; Plastron: Mann-Whitney U test: U=19, P=0.21) (S7 Table).

Rearranging the variables according to presence, frequency and times (Table 1), and through the multidimensional scaling, we obtained a total of 10 variables: five correlated with the shiest individuals and other five associated with the boldest individuals (Fig 5).

These variables were characteristic of the shier turtles: (i) hiding in a new environment, (ii) not crossing a new environment, (iii) high quiet time in a new environment, (iv) high straightening time during predation, and (v) low walking time in a new environment. Variables for boldest turtles were: (i) not hiding in a new environment, (ii) cross a new environment, (iii) low quiet time in a new environment, (iv) low straightening time during predation, and (v) high walking time in a new environment (Fig 5). Four of those variables were also statistically significant for the discrimination of the two groups (Fig 6, S8 Table).

Table 1. Variables selected for simulations, subdivision used in correlation analysis and multidimensional scaling with relative abbreviation.

**Variable**	**Subdivision for analysis**	**Abbreviation**
Kicking during manipulation (KM)	Fast kicking	FK

	Slow kicking	SK
Neck stretched during manipulation (NeSM)	Yes neck stretched manipulation	YNeSM
	No neck stretched manipulation	NoNeSM
Hiding inside shell during manipulation (HSM)	Yes hide shell manipulation	YHSM
	No hide shell manipulation	NoHSM
Neck retracted during manipulation (NeRM)	Yes neck retracted manipulation	YNeRM
	No neck retracted manipulation	NoNeRM
Straightening time during predation (STP)	High straightening time predation	HSTPr
	Low straightening time predation	LSTPr
Hiding inside shell during predation (HSP)	Yes hide shell predation	YHSPr
	No hide shell predation	NoHSPr
Walking time in a new environment (WTNE)	High walking time new environment	HWTNE
	Low walking time new environment	LWTNE
Hiding in a new environment (HNE)	Yes hide new environment	YHNE
	No hide new environment	NoHNE
Quiet time in new environment (QTNE)	High quiet time new environment	HQTNE
	Low quiet time new environment	LQTNE
Crossing a new environment (CrNE)	Yes cross area	YCrNE
	No cross area	NoCrNE
Exploration peluche (ExP)	Yes exploring peluche	YExP
	No exploring peluche	NoExP
Exploring time peluche (ExTP)	High time exploring peluche	HExTP
	Low time exploring peluche	LExTP

Table 2. Presence (=1) or absence (=0) of variables selected for simulation and boldness index values. In grey background color the specimens considered bolder, in white those considered shier.

Turtle Name	Sum variables	Boldness Index	KM	NeSM	HSM	NeRM	STD	HSPr	WTNE	HNE	QTNE	CrNE	ExP	ExTP
**Alfredo**	9	0,75	1	1	1	1	1	0	0	1	0	1	1	1
**Benedicto**	12	1	1	1	1	1	1	1	1	1	1	1	1	1
**Carlos**	8	0,67	1	1	1	1	1	0	0	1	1	1	0	0
**Hector**	8	0,67	1	1	1	0	1	0	1	1	1	0	1	0
**Nestor**	8	0,67	1	1	1	1	0	0	1	1	1	1	0	0
**Erik**	7	0,58	0	0	0	0	1	1	1	1	1	1	1	0
**Garry**	7	0,58	0	1	0	0	1	0	1	1	1	0	1	1
**John**	10	0,83	0	1	1	1	1	1	1	1	1	1	1	0
**Oliver**	8	0,67	1	0	1	0	1	0	1	1	1	1	1	0
**Francesco**	5	0,41	1	1	1	0	0	0	0	1	1	0	0	0
**Kevin**	3	0,25	0	0	1	0	0	0	0	0	0	0	1	1
**Marc**	5	0,41	1	1	1	1	0	0	0	1	0	0	0	0
**Patricio**	3	0,25	0	0	1	0	0	0	0	0	0	0	1	1
**Denis**	6	0,5	1	1	1	0	1	0	0	1	0	1	0	0
**Ian**	6	0,5	1	1	1	1	1	1	0	0	0	0	0	0
**Lorenzo**	5	0,41	1	0	1	0	1	1	0	0	0	0	1	0

Temperament as a modulating factor during the interaction between male and female

As results for the relation between temperaments and male and female interaction, we observed behaviors linked with courtship and others with mating.

Regarding courtship, we determined 16 behaviors (S3 Table), but we used 11 of them for the analysis, excluding behaviors showed by most of the specimens and the ones that did not show differences between the groups.

We defined if they occurred or not and how often to obtain their frequency (low or high) (Table 3). We observed that the following traits are linked with the shier individuals (Fig 7): (i) pushing the female, (ii) blocking the female, (iii) frontal approach with stretched legs and retracted neck (iv) pushing the ground with front leg, (v) approaching when excited. On the other hand, some bolder turtles did not show courtship behavior while in others they were not very frequent. Bolder individuals are linked with: (i) do not block the female, (ii) do not push it, (iii) do not approach excitedly, (iv) do not walk backwards, (v) low frequency of frontal approach with stretched legs and extended neck (Fig 7).

Four behaviors (Table 4, S4 Table) were observed for mating. None of those behaviors is displayed by shiest individuals, while the boldest are more linked with intent of mounts, and mount at high frequency (Fig 8, S1 Video).

Table 3. Courtship behaviors obtained from simulations male vs female, subdivision used in correlation analysis and must-dimensional scale with relative abbreviation.

**Variable**	**Subdivision**
Frontal approach with stretched legs and stretched neck	High Frequency (HFASLSN)<br>Low Frequency (LFASLSN)<br>No Approach (NoFASLSN)
Frontal approach with stretched legs and retracted neck	High Frequency (HFASLRN)<br>Low Frequency (LFASLRN)<br>No Approach (NoFASLRN)
Cross snouts	High Frequency (HCrS)<br>Low Frequency (LCrS)<br>No Cross (NoCrS)
Push ground with front leg	High Frequency (HPGL)<br>Low Frequency (LPGL)<br>No Push (NoPGL)
Block the female	Yes Blocking (YBlock)<br>No Blocking (NoBlock)
Pushing the female	Yes Pushing (YPush)<br>No Pushing (NoPush)
Walk backwards	High Frequency (HWB)<br>Low Frequency (LWB)<br>No Walk (NoWB)
Excited male is approaching	Yes excited male (YEMA)<br>No excited male (NoEMA)
Quiet male explores surroundings	Yes Explore (YQMExp)<br>No Explore (NoQMExp)
Cloaca sniffing	High Frequency (HCS)<br>Low Frequency (LCS)
Remote tracking	Yes Remote (YRT)<br>No Remote (NoRT)

Table 4. Mating behaviors obtained from simulations male vs female, subdivision used in correlation analysis and must-dimensional scale with relative abbreviation.

Variable	Subdivision
Neck stimulation	High Frequency (HNS)
	Low Frequency LNS)
	No Stimulation (NoNS)
Mount attempt	High Frequency (HMA)
	Low Frequency (LMA)
	No Mount attempt (NoMA)
Coitus	Yes Coitus (YCo)
	No Coitus (NoCo)
Mount	High Frequency (HM)
	Low Frequency (LM)
	No Mount (NoM)

Temperament as a modulating factor during the competitive interaction between males in the presence of a female

We obtained 12 behaviors related to courtship (Table 5, S5 Table). We considered temperament of the two males together with a female and observed that courting variables are concentrated around the boldest regardless the temperament of their opponent (Fig 9). On the contrary, the shiest did not display particular behaviors except for blocking the female during the tracking (Fig 9) (S2 Video).

Table 5. Courtship behaviors obtained from simulations male vs male vs female used in correlation analysis and must-dimensional scale with relative abbreviation.

Variable	Abbreviation
Approaching to cloaca	AC
Stretching neck towards female	SN
Cloaca sniffing	CS
Lateral sniffing	LS
Tracking	T
Quiet alongside female	Q
Bite	Bi
Blocking female	Bl
Lean leg to female	LL
Walk backwards	WB
Push female	PF
Push ground with leg	PGL

Fig 5. Distribution of the variables (behaviors) of the specimens. The red circle shows variables linked with the boldest specimens, the blue circle identifies behaviors linked to the shiest ones.

Fig 6. Mean values and standard errors of the four significant binomial variables. High time walking new environment, Bolder: mean±SE = 0,78±0,1, Shier: mean±SE= 0±0, Mann-Whitney U test: U=7, p=0.00*3; Not hiding new environment, Bolder: mean±SE = 1±0, Shier: mean±SE= 0,43±0,2, Mann-Whitney U test: U=13,5, p=0.013*; Low time quiet new

environment, Bolder: mean±SE = 0,89±0,1, Shier: mean±SE= 0,14±0,14, Mann-Whitney U test: U=37, p=0.004*; Cross new environment, Bolder: mean±SE = 0,78±0,14, Shier: mean±SE= 0,14±0,1, Mann-Whitney U test: U=37, p=0.017*.

Fig 7. Distribution of courtship variables (behaviors) of the male specimens. The red curve includes variables linked with boldest specimens, the blue curve congregates behaviors linked with the shiest individuals.

Fig 8. Distribution of mating variables (behaviors) of the male specimens. The red curve encompasses variables linked with boldest individuals, the blue curve includes behaviors linked with the shiest ones.

Fig. 9. Distribution of courtship variables (behaviors) of the specimens. The red circle delimits variables highly linked with boldest males in competition with each other, the yellow circle encompasses those highly linked with a bold male in competition with a shy one, the green ellipse includes behaviors linked with the boldest not considering who is its competitor.

Discussion

A variety of methods were used but separately to determine temperament in aquatic and terrestrial turtles [4,35-39]. This is the first paper that tests all of them. In this study, we tested reaction to: manipulation, predation risk, a new environment and the introduction of a novel object with the purpose of looking for consistency, to determine the most accurate behaviors and in consequence the best method to define the level of boldness in turtles. Behaviors observed in a new environment seem to be the best to determine the boldness in turtles: hiding and limited walk for the shiest, being quiet (shiest) or not (boldest), walking

a lot and crossing the new environment for the boldest. These results coincide with two of previous works [37-38].

The test with the risk of predation also shows that the time to straighten is linked with the boldness as observed in previous studies [35-36].

Our work focused only on males, and the determination of temperament in females or juveniles may be different even if the exploration of a new environment may be equivalent whatever the sex and age of turtles that generally share the same strategy of life whatever their conditions. Therefore, to assure that behaviors linked with the reaction to a new environment can be a better method, we suggest further studies to test it also in females and juveniles.

An interesting element that emerges in this study is the modulation of males´ behavior.

When a bold male is alone with a female, he often skips courtship behaviors to devote himself to stimulating the female for mounting and mating; when the same male is found in the presence of another conspecific male, a potential sexual competitor, the first engages in courtship behaviors towards the female. A similar result has been observed in the common water strider *Aquarius remiges* (Gerridae, Insecta), where the most active and aggressive males spend most of their time looking for a female as well as having the highest copula rates [46], and in the common lizard *Zoocora vivipara* (Lacertidae, Reptilia), where females prefer the more active males when they find themselves alone and without the presence of predators in their surroundings, whereas when shier specimens find themselves alone in the presence of a female, they spend time and energy in courtship behaviour [47].

In our simulations, mating attempts of shier males were very rare; when the shier is in presence of a competitor and a female, it ignores the female. In summary, boldest individuals court the female only in presence of a potential competitor and directly mate with

her without courtship if no competitor is present. Shier males courtship the female only if no competitor is present and is not very effective at mating. This kind of behavior towards a competitor was observed in *Mauremys leprosa* where responses to chemicals cues depended on boldness; in particular, shy turtles avoided chemical cues from familiar and unfamiliar bolder males [35]. However, it is possible for shier animals that more time is necessary to display a mating behavior, anyway our work shows apparent differences in the sexual behavior according to males' temperaments. Therefore, we can argue that the degree of boldness can influence males' reproductive behavior, in decision making and their reaction according to the situation. In terms of costs and benefits, boldness can be considered a factor that allows specimens to evaluate how to act during courtship and mating considering the presence of conspecifics of both sexes. On the other hand, it avoids competition and the possibility of losing energy when a bolder male is competing. In other species, male competition could be very costly because of fights, injuries, territory care and predatory danger during courtship and refraining those costs could also be an alternative strategy to avoid damages and predators, as shown in the Iberian wall lizard *Podarcis hispanica,* which uses chemical cues during competitive matches to recognize competitors and reduce intensity and costs of the fights [48]. The success of different strategies dependant of the temperament is under environmental constraint such as the presence of predators as observed for other animals [1]. For example, the broadhead skink *Eumeces* (*Plestiodon*) *laticeps* shows a sensitivity in predation risk during reproductive opportunities: when females were present, isolated and mate-guarding males initiate courtship, but isolated males permitted closer approach by a predator than did mate-guarding males. Moreover, there is a high level of latency by isolated males when a predator is close to the female so they were able to balance risk of predation with courtship behavior [49].

Researchers have already discussed that behavior plays a key role in animal survival and evolution, behavioral traits can help species to adapt to rapid changes in the environment [50], and survive to natural selection [51] as much as morphological and physiological traits. Differences in sexual behavior related to boldness or other behavioral traits, can be an honest signal of mate quality [52]. Therefore, identifying those behavioral traits that can affect population viability is important to provide helpful information to integrate conservation plans [53].

The information obtained in this study represents an important tool for the conservation of reptiles and in particular for land turtles. Worldwide, turtles represent one of the vertebrate groups with the highest risk of extinction [54]. In recent years, many researchers have ensured the importance of not only increasing the behavioral information of the species, but also implementing these data in their conservation and monitoring strategies [11,55].

To our best knowledge, this is the first work that connects temperament to reproductive behaviors in land turtles. What we have achieved can be used as a first step for compatibility plans for turtles, in order to combine temperament traits that might compose a successful breeding couple [11].

Temperament studies in couple compatibility have significant implications in *ex situ* conservation programs, since specific traits (such as aggressiveness and fear) have been related to reproductive success in some species raised in zoos [12]. In addition, certain temperaments may be more likely to thrive in captive environments, artificial selection for or against specific temperament traits may accelerate domestication processes, making animals raised for conservation less suitable for release in wildlife [26].

Our research showed that bolder males need less time to mount and mate, with perhaps higher fitness than shier ones.

Another application of our findings is the possibility to keep the whole range of boldness. Considering behavioral variation, it is important to preserve boldness since it allows conservationists to reintroduce animals with all the behavioural traits, giving them proper tools to survive in novel environments [51]. Considering this, in order to not to lose the behavioral pool during captive reproduction, we must be aware of the needs of different specimens. Therefore, our study has allowed to understand demonstrates that shy turtles likely need more time for mating and need to be alone with the female, otherwise they lose interest. For the boldest animals, the necessary times are lower and they are potentially able to reproduce even with the presence of conspecifics.

Acknowledgments

We would like to thank El Colegio de la Frontera del Sur and the CONACYT-OEA-AMEXCID scholarship program for the funds needed to cover expenses for data collection and specimen maintenance; the citizens of Chetumal who gave and loaned specimens of *R. areolata* for this research; José Alberto García Angulo for his help in the design and construction of the turtle maintenance and observation areas; Dr. Gabriele Gheza, Martina Anna Belli and Antonin Mareš for their help in grammatical and linguistic revision.

References

[1] Réale D, Reader S, Sol D, McDougall PT, Dingemanse NJ. Integrating animal temperament within ecology and evolution. Biol Rev. 2007;82: 291-318.

[2] Pervin L, John OP. Handbook of personality: theory and research. 2nd ed. New York: Guilford; 1999.

[3] MacKay JRD, Haskell MJ. Consistent individual behavioral variation: the difference between temperament, personality and behavioral syndromes. Animals. 2015;5: 455-478.

[4] Germano JM, Nafus MG, Perry JA, Hall DB, Swaisgood RR. Predicting translocation outcomes with personality for desert tortoises. Behav Ecol. 2017;28: 1075-1084.

[5] Dingemanse NJ, Araya-Ajoy, YG. Interacting personalities: behavioural ecology meets quantitative genetics. Trends Ecol Evol. 2015;31: 88-97.

[6] Dingemanse NJ, Réale D. 2005. Natural selection and animal personality. Behavior. 2005;142: 1165-1190.

[7] Smith BR, Blumstein DT. Fitness consequences of personality: a meta-analysis. Behav Ecol. 2008;19: 448-455.

[8] Chapman JW, Klaassen RH, Drake VA, Fossette S, Hays GC, Metcalfe JD, Reynolds AM, Reynolds DR, Alerstam T. Animal orientation strategies for movement in flows. Curr Biol. 2011;21: R861-R870.

[9] Duckworth RA, Badyaev AV. Coupling of dispersal and aggression facilitates the rapid range expansion of a passerine bird. Proc Natl Acad Sci. U.S.A. 2007;104: 15017-15022.

[10] Dyer JRG, Croft DP, Morrell LJ, Krause J. Shoal composition determines foraging success in the guppy. Behav Ecol. 2009;20: 165-171.

[11] Tetley CL, O'Hara SJ. Ratings of animal personality as a tool for improving the breeding, management and welfare of zoo mammals. Anim Welf. 2012;21: 463-476.

[12] Martin-Wintle MS, Shepherdson D, Zhang G, Huang Y, Luo B, Swaisgood RR. Do opposites attract? Effects of personality matching in breeding pairs of captive giant pandas on reproductive success. Biol Conserv. 2017;207: 27-37.

[13] Kelleher SR, Silla AJ, Byrne PG. Animal personality and behavioral syndromes in amphibians: a review of the evidence, experimental approaches, and implications for conservation. Behav. Ecol Sociobiol. 2018;72: 79.

[14] Gowaty PA, Drickamer LC, Schmid-Holmes S. Male house mice produce fewer offspring with lower viability and poorer performance when mated with females they do not prefer. Anim Behav. 2003;65: 95-103.

[15] Ah-King M, Gowaty PA. A conceptual review of mate choice: stochastic demography, within-sex phenotypic plasticity, and individual flexibility. Ecol. Evol. 2016;6: 4607-4642.

[16] Wilson ADM, Godin J-GJ. Boldness and behavioral syndromes in the bluegill sunfish, *Lepomis macrochirus*. Behav Ecol. 2009;20: 231-237.

[17] Cole EF, Quinn JL. Shy birds play it safe: personality in captivity predicts risk responsiveness during reproduction in the wild. Biol. Lett. 2014;10: 20140178.

[18] White SJ, Pascall DJ, Wilson AJ. Towards a comparative approach to the structure of animal personality variation. Behav Ecol. 2020;31: 340-351.

[19] Powell D, Lin H, Carlstead K, Kleiman D, Zhang H, Zhang G, et al. Relationships between temperament, husbandry, management, and socio-sexual behavior in captive male and female giant pandas (*Ailuropoda melanoleuca*). Acta Zool Sin. 2008;54: 169-175.

[20] Schwartz MK. Guidelines on the use of molecular genetics in reintroduction programs. In: The EU LIFE-Nature Projects to guidelines for the reintroduction of threatened species. March 21-22; Caramanico Terme, Italy; 2005. pp 51-58.

[21] Miller W, Wright SJ, Zhang Y, Schuster SC, Hayes VM. Optimization methods for selecting founder individuals for captive breeding or reintroduction of endangered species. In Biocomputing 2010. pp 43-53.

[22] Saidenberg ABS, Zuniga E, Melville PA, Salaberry S, Benites NR. Health-screening protocols for vinaceous amazons (*Amazona vinacea*) in a reintroduction project. J Zoo Wildl Med. 2015;46: 704-712.

[23] Kleiman DG. Foreword: animal behaviour studies and zoo propagation programs. Zoo Biol. 1994;13: 411-412.

[24] Bremner-Harrison S, Prodohl PA, Elwood RW. Behavioural trait assessment as a release criterion: boldness predicts early death in a reintroduction programme of captive-bred swift fox (*Vulpes velox*). Anim Conserv Forum. 2004; 313-320.

[25] Carlstead K, Mellen J, Kleiman DG. Black rhinoceros (*Diceros bicornis*) in US zoos: I. Individual behavior profiles and their relationship to breeding success. Zoo Biol. 1999a;18: 17-34.

[26] McDougall PT, Réale D, Sol D, Reader SM. Wildlife conservation and animal temperament: causes and consequences of evolutionary change for captive, reintroduced, and wild populations. Anim Conserv. 2006;9: 39-48.

[27] Freeman EW, Guagnano G, Olson D, Keele M, Brown JL. Social factors influence ovarian acyclicity in captive African elephants (*Loxodonta africana*). Zoo Biol. 2009;28: 1-15.

[28] Lees CM, Wilcken J. Sustaining the ark: the challenges faced by zoos in maintaining viable populations. Int Zoo Yearb. 2009;43: 6-18.

[29] Beach FA and Leboeuf BJ Coital behaviour in dogs. I. Preferential mating in the bitch. Anim Behav. 1967;15: 546-548.

[30] Sánchez-Macouzet O, Rodríguez C, Drummond H. Better stay together: pair bond duration increases individual fitness independent of age-related variation. Proc R Soc B. 2014;281: 20132843.

[31] Schuett W, Dall SR, Royle NJ. Pairs of zebra finches with similar ‘personalities’ make better parents. Anim Behav. 2011;81: 609-618.

[32] Rangassamy M, Dalmas M, Féron C, Gouat P, Rödel HG. Similarity of personalities speeds up reproduction in pairs of a monogamous rodent.Anim Behav. 2015;103: 7-15.

[33] Carlstead K, Fraser J, Bennett C, Kleiman DG. Black rhinoceros (*Diceros bicornis*) in US zoos: II. Behavior, breeding success, and mortality in relation to housing facilities. Zoo Biol. 1999b;18: 35-52.

[34] Ibáñez A, Polo-Cavia N, López P, Martín J. Honest sexual signaling in turtles: experimental evidence of a trade-off between immune response and coloration in red-eared sliders *Trachemys scripta elegans*. Naturwissenschaften. 2014;101: 803-811

[35] Ibáñez A, Marzal A, López P, Martín J. Boldness and body size of male Spanish terrapins affect their responses to chemical cues of familiar and unfamiliar males. Behav. Ecol Sociobiol. 2013;67: 541-548.

[36] Ibáñez A, Martín J, Gazzola A, Pellitteri-Rosa D. Freshwater turtles reveal personality traits in their antipredatory behaviour. Behav Process. 2018;157: 142-147.

[37] Carter AW, Paitz RT, McGhee KE, Bowden RM. Turtle hatchlings show behavioral types that are robust to developmental manipulations. Physiol Behav. 2016;155: 46-55.

[38] Mafli A, Wakamatsu K, Roulin A. Melanin-based coloration predicts aggressiveness and boldness in captive Eastern hermann's tortoises. Anim Behav. 2011;81: 859-863.

[39] Kashon EAF, Carlson BE. Consistently bolder turtles maintain higher body temperatures in the field but may experience greater predation risk. Behav. Ecol Sociobiol. 2018;72: 9.

[40] Vogt RC, Platt, SG, Rainwater TR. *Rhinoclemmys areolata* (Duméril and Bibron 1851), Furrowed wood turtle, Black-bellied turtle, Mojina. Chelonian Res. Monogr. 2009;5: 22-27.

[41] Cupul Cicero V, Aguilar Cordero WDJ., Chablé Santos J, Sélem Salas CI. Conocimiento etnozoológico de la herpetofauna de la comunidad maya de Santa Elena, Yucatán, México. Estud Cult Maya. 2019;54: 285-314.

[42] van Dijk PP, Lee J, Calderón Mandujano R, Flores-Villela O, Lopez-Luna MA. *Rhinoclemmys areolata*. *The IUCN Red List of Threatened Species. 2007.*

[43] Pérez-Higareda G, Smith HM. Courtship behavior in *Rhinoclemmys Areolata* from western Tabasco, Mexico (Testudines: *Emydidae*). The Gt. Basin Nat. 1988;48: 263-266.

[44] Artner H. Field observations and captive breeding of the furrowed wood turtle *Rhinoclemmys areolata* (Duméril and Bibron, 1851). Emys. 2009;16: 4-19.

[45] Pearse DE, Avise JC. Turtle mating systems: behavior, sperm storage, and genetic paternity. J Hered. 2001;92: 206-211.

[46] Sih, A, Bell, A, Johnson CJ, Ziemba RE. Behavioral syndromes: an integrative overview. Q Rev Biol. 2004;79: 241-277.

[47] Teyssier A, Elvire Bestion E, Richard M, Cotea J. Partners' personality types and mate preferences: predation risk matters. Behav Ecol. 2014;25: 723-733.

[48] López P, Martín J. Chemical rival recognition decreases aggression levels in male Iberian wall lizards, *Podarcis hispanica*. Behav Ecol. Sociobiol. 2002;51: 461-465.

[49] Cooper Jr. WE. Tradeoffs between courtship, fighting, and antipredatory behavior by a lizard, *Eumeces laticeps*. Behav Ecol Sociobiol. 1999;47: 54-59.

[50] Angeloni L, Crooks KR, Blumstein DT. Breed MD, Moore J, editors. Conservation and behavior: Introduction. 2010. pp. 377–381. Encyclopedia of animal behavior, Volume 1. Oxford: Academic Press.

[51] Caro T, Sherman PW. Endangered species and a threatened discipline: behavioural ecology. Trends Ecol Evol. 2011;26: 111–118.

[52] Merrick MJ, Koprowski JL. Should we consider individual behavior differences in applied wildlife conservation studies? Biol Conserv. 2017;209: 34-44.

[53] Ahtiainen JJ, Alatalo RV, Mappes J, Vertainen L. Decreased sexual signalling reveals reduced viability in small populations of the drumming wolf spider *Hygrolycosa rubrofasciata*. Proc R Soc London B. 2004;271: 1839-1845.

[54] Rhodin AG, Stanford CB, Van Dijk PP, Eisemberg C, Luiselli L, Mittermeier RA, et al. Global conservation status of turtles and tortoises (Order *Testudines*).Chelonian Conserv Biol. 2018;17: 135-161.
[55] Tadesse SA. Animal behavior in conservation biology. Intl J Avian Wildl Biol. 2018;3: 25-26.

Supporting information

Table S1. List of responses found in behavioral simulation (occurrence=1, absence=0), with relative abbreviation and subdivision applied for statistical analysis. In the first column, there are names of each specimen, in grey background color the bolder and in white the shier.

S2 Table. Straight carapace and straight plastron length measurements of each male turtle. In grey background color the specimens considered bolder, in white those considered shier.

S3 Table. List of courtship behaviors during male and female simulation (occurrence=1, absence=0), with relative abbreviation and subdivision applied for statistical analysis. In the first column, there are names of each specimen, in grey background color the bolder and in white the shier.

S4 Table. List of mating behaviors during male and female simulation (occurrence=1, absence=0), with relative abbreviation and subdivision applied for statistical analysis.

In the first column, there are names of each specimen, in grey background color the bolder and in white the shier.

S5 Table. List of courtship behavior during male vs male competition in presence of female (occurrence=1, absence=0), with relative abbreviation and subdivision applied for statistical analysis. In the first column, there are names of each specimen, in red the bolder and in blue the shier. Columns 2,3,4 and 5 indicate pairs by temperament (BB=boldVSbold, BS=boldVSshy, SS=shyVSshy, SB=shyVSbold).

S6 Table. List of mating behavior during male vs male competition in presence of female (occurrence=1, absence=0), with relative abbreviation and subdivision applied for statistical analysis. In the first column, there are names of each specimen, in grey background color the bolder and in white the shier. Columns 2,3,4 and 5 indicate pairs by temperament (BB=boldVSbold, BS=boldVSshy, SS=shyVSshy, SB=shyVSbold).

S7 Table: Statistical results of Mann-Whitney test on length measurements.

S8 Table: Statistical results of Mann-Whitney test on variables selected for boldness index, significant variables are indicated by *.

S1 Video. Shy courtship and Bold mount.

S2 Video. Shy and bold male in presence of a female. Shy is walking around the arena while the bold courtships the female.

Figure1

Click here to access/download;Figure;Fig 1. Details of Rhinoclemmys areolata

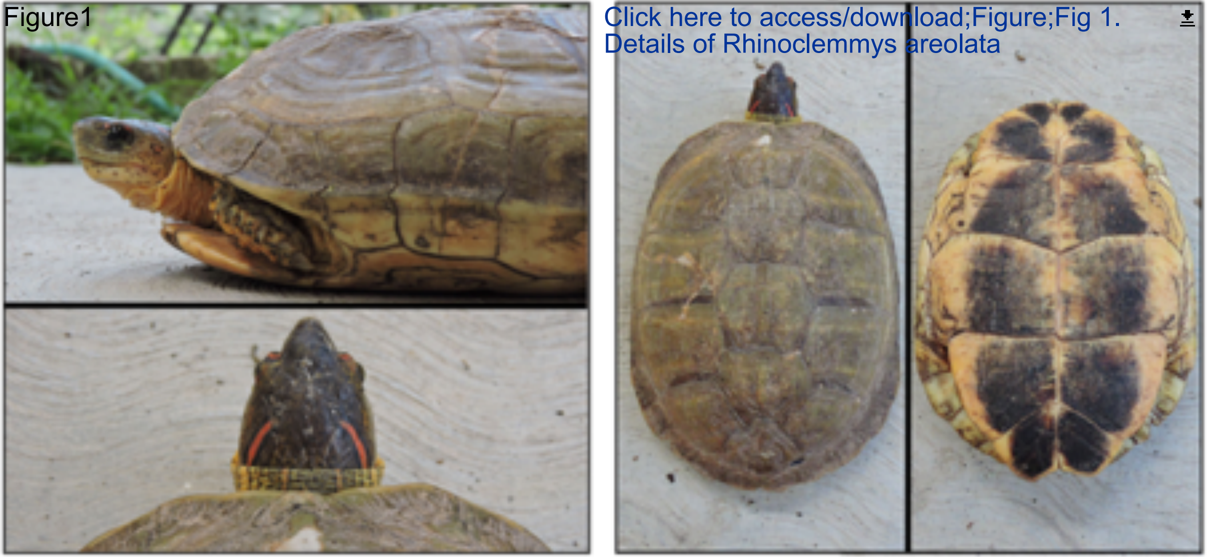

Figure2

Click here to access/download;Figure;Fig 2.
Detail of female (left) and male (right).pdf

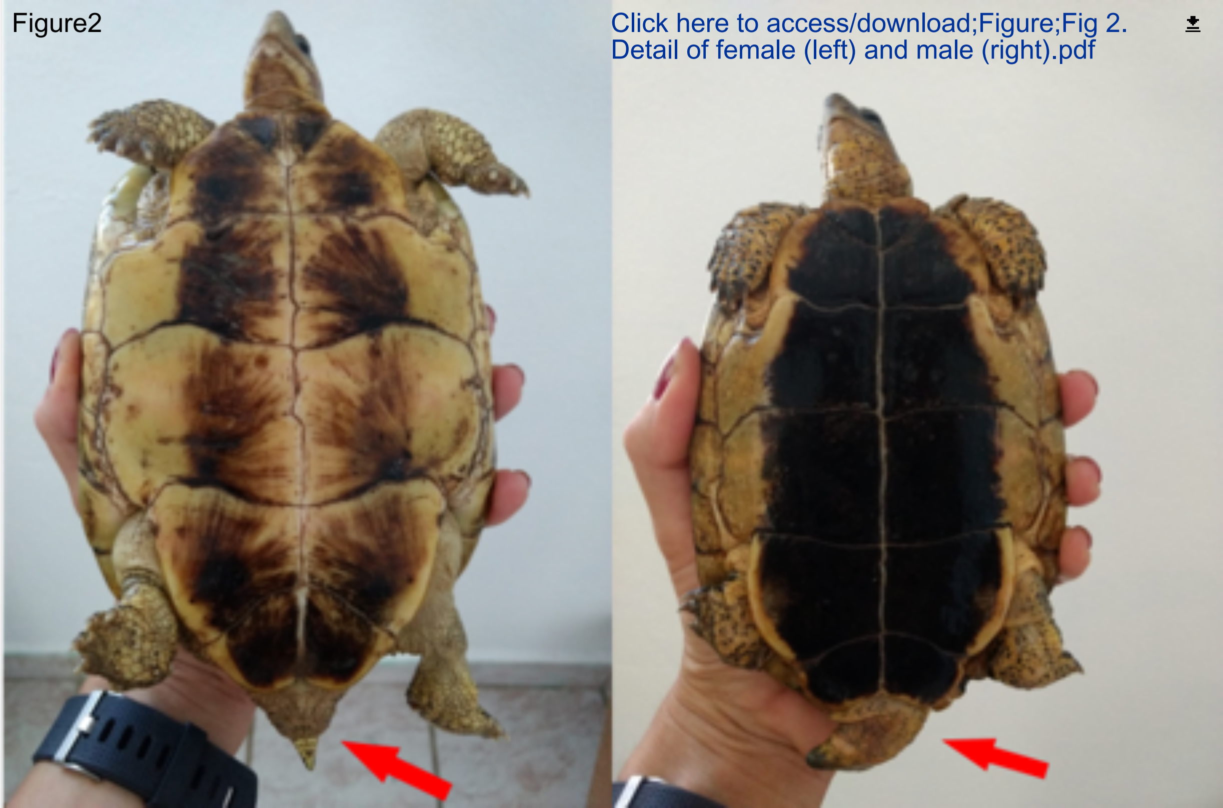

Figure3

Click here to access/download;Figure;Fig 3.pdf

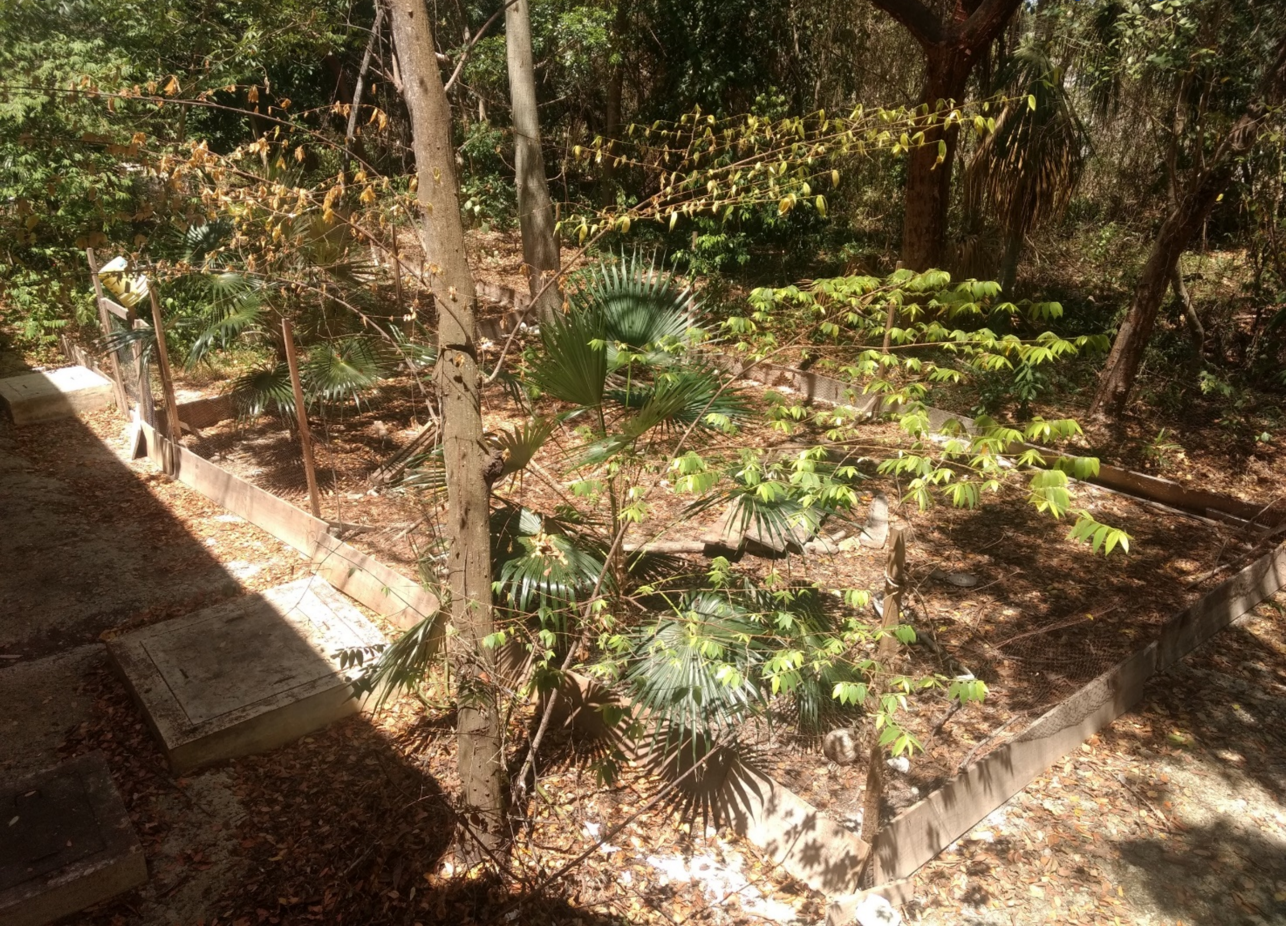

Figure4

Click here to access/download;Figure;Fig 4.pdf

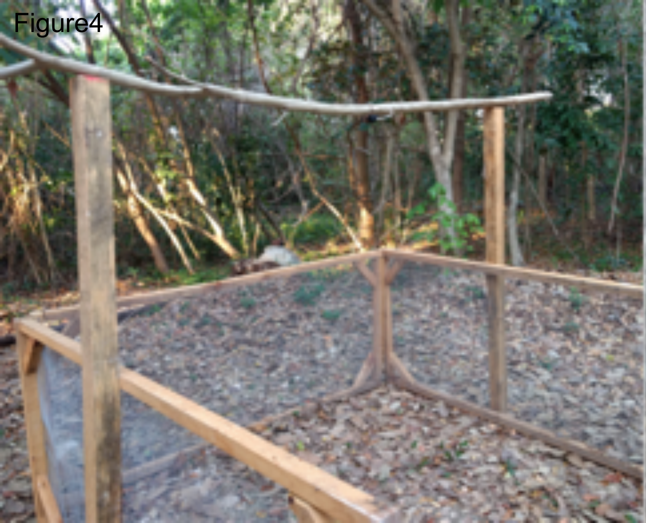

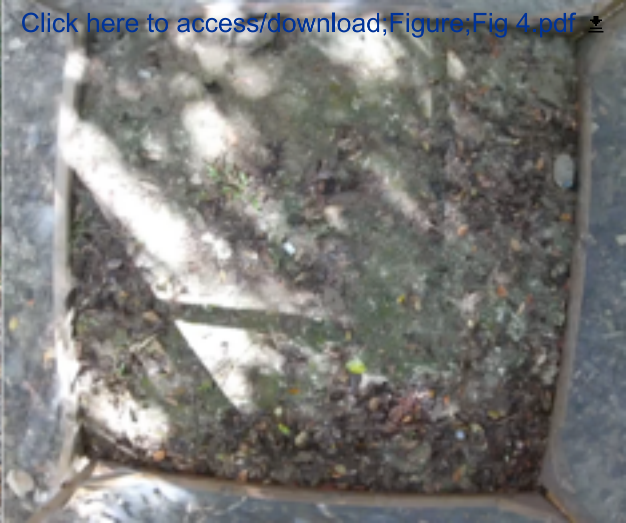

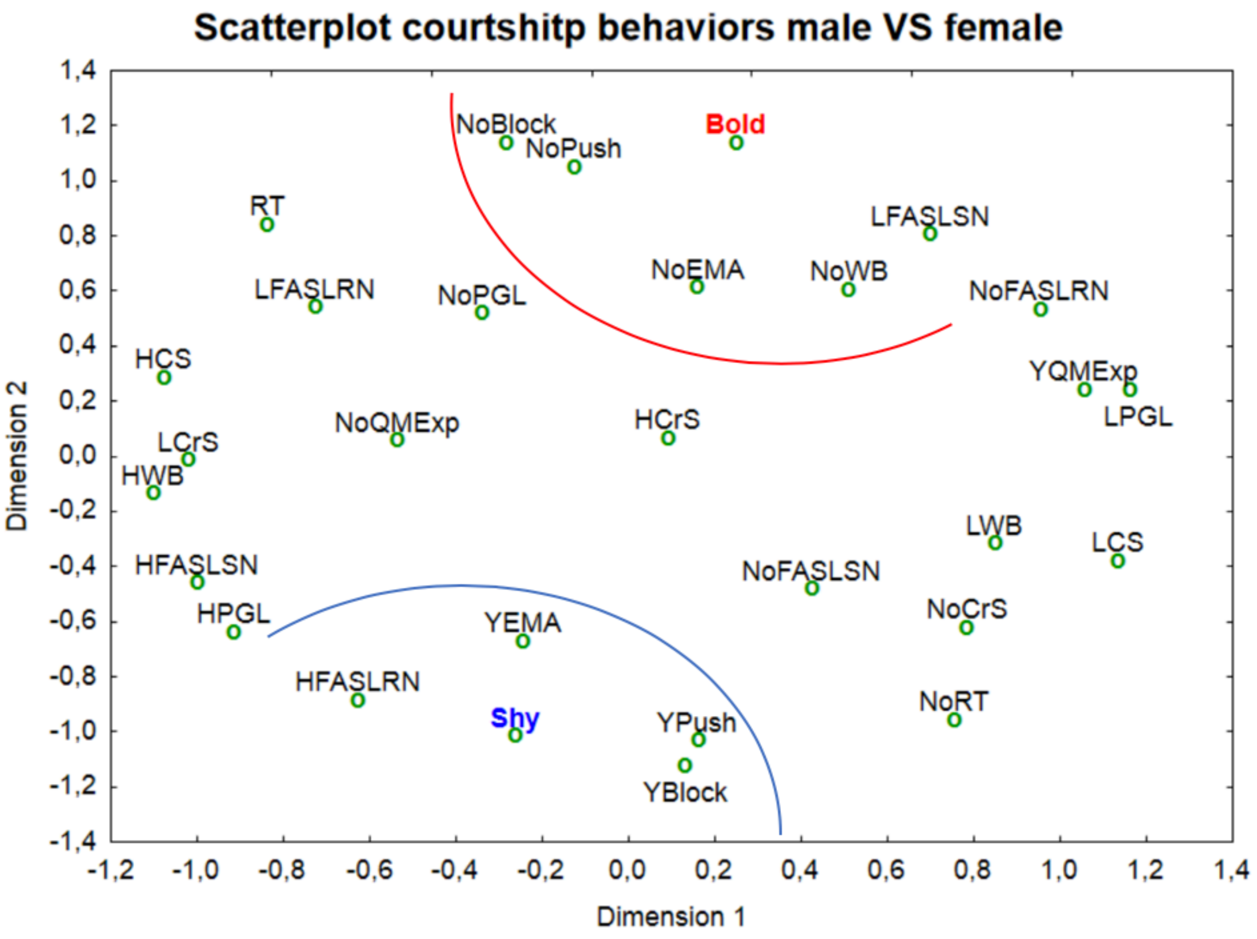
Scatterplot courtshitp behaviors male VS female
Dimension 1
Dimension 2
1,4
1,2
1,0
0,8
0,6
0,4
0,2
0,0
-0,2
-0,4
-0,6
-0,8
-1,0
-1,2
-1,4
-1,2
-1,0
-0,8
-0,6
-0,4
-0,2
0,0
0,2
0,4
0,6
0,8
1,0
1,2
1,4
NoBlock
NoPush
Bold
RT
LFASLSN
NoEMA
NoWB
LFASLRN
NoPGL
NoFASLRN
HCS
YQMExp
LPGL
NoQMExp
HCrS
LCrS
HWB
LWB
LCS
HFASLSN
NoFASLSN
HPGL
NoCrS
YEMA
HFASLRN
NoRT
Shy
YPush
YBlock

Figure6

Click here to access/download;Figure;Fig 6.pdf

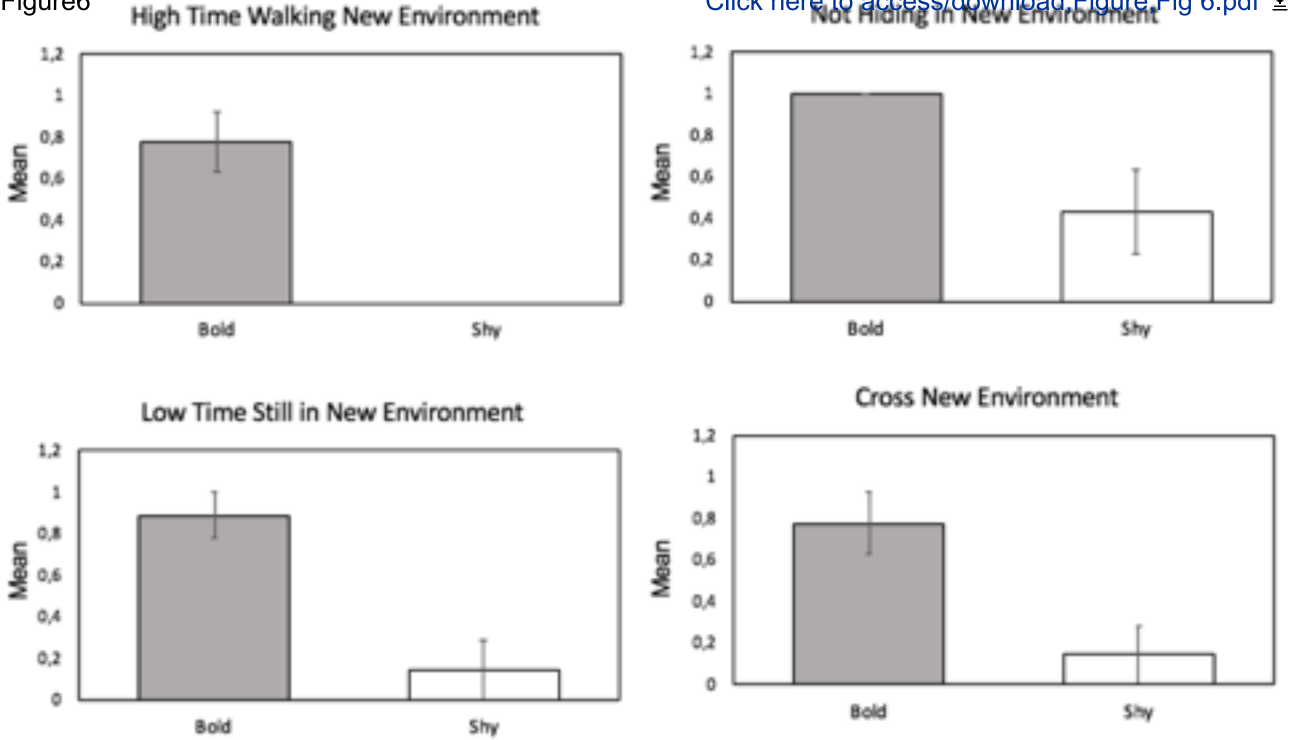

Figure7

Click here to access/download;Figure;Fig 7.pdf

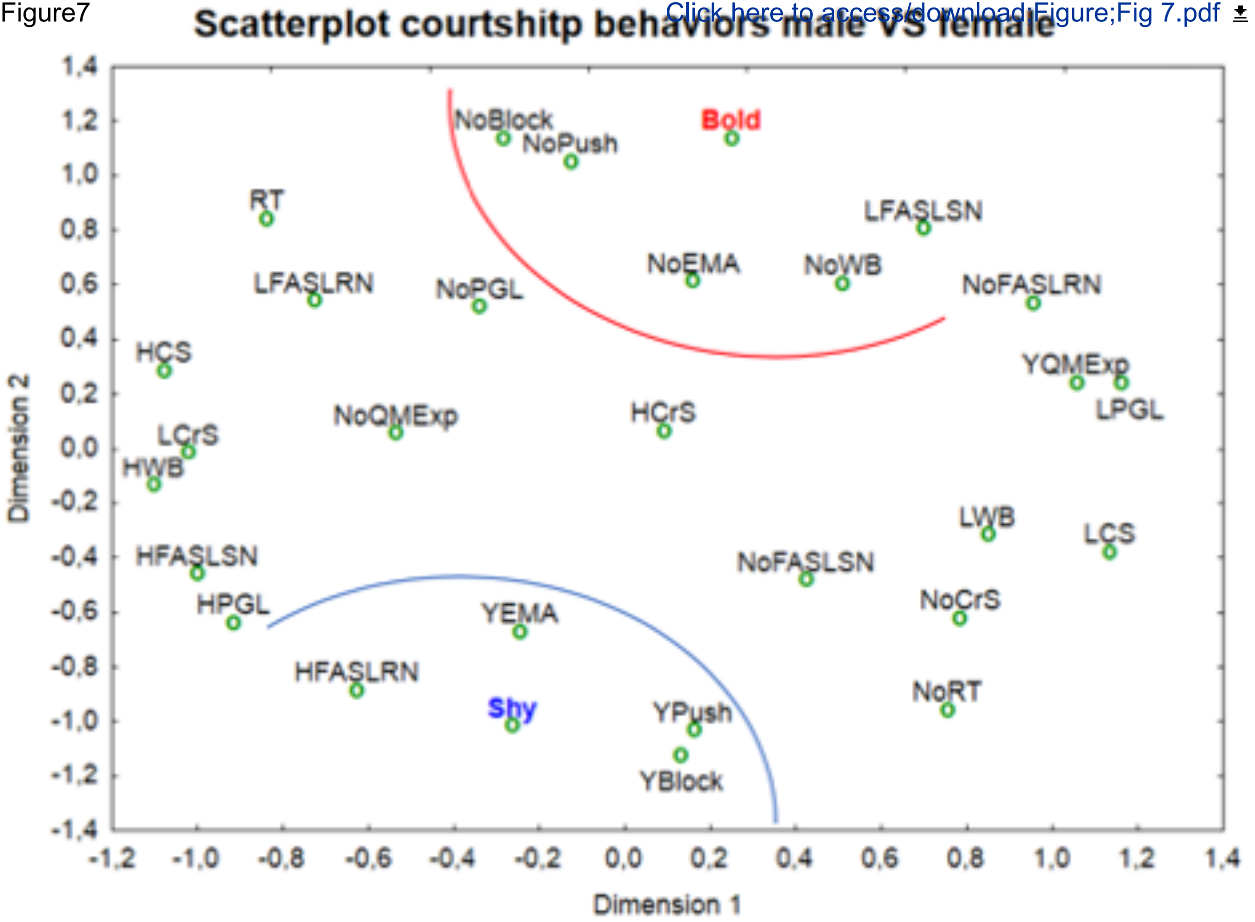

Figure8

Click here to access/download;Figure;Fig 8.pdf

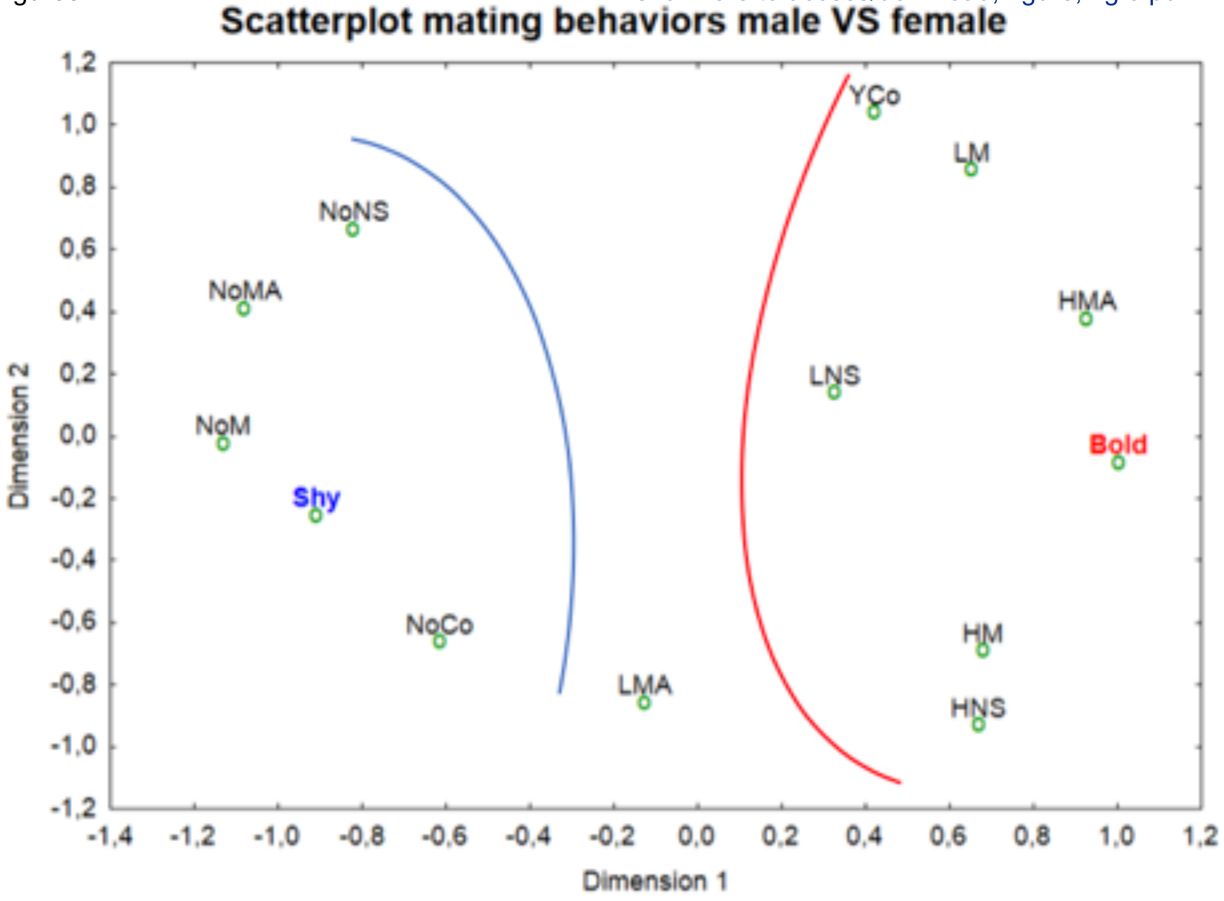

Figure9

Click here to access/download;Figure;Fig. 9.pdf

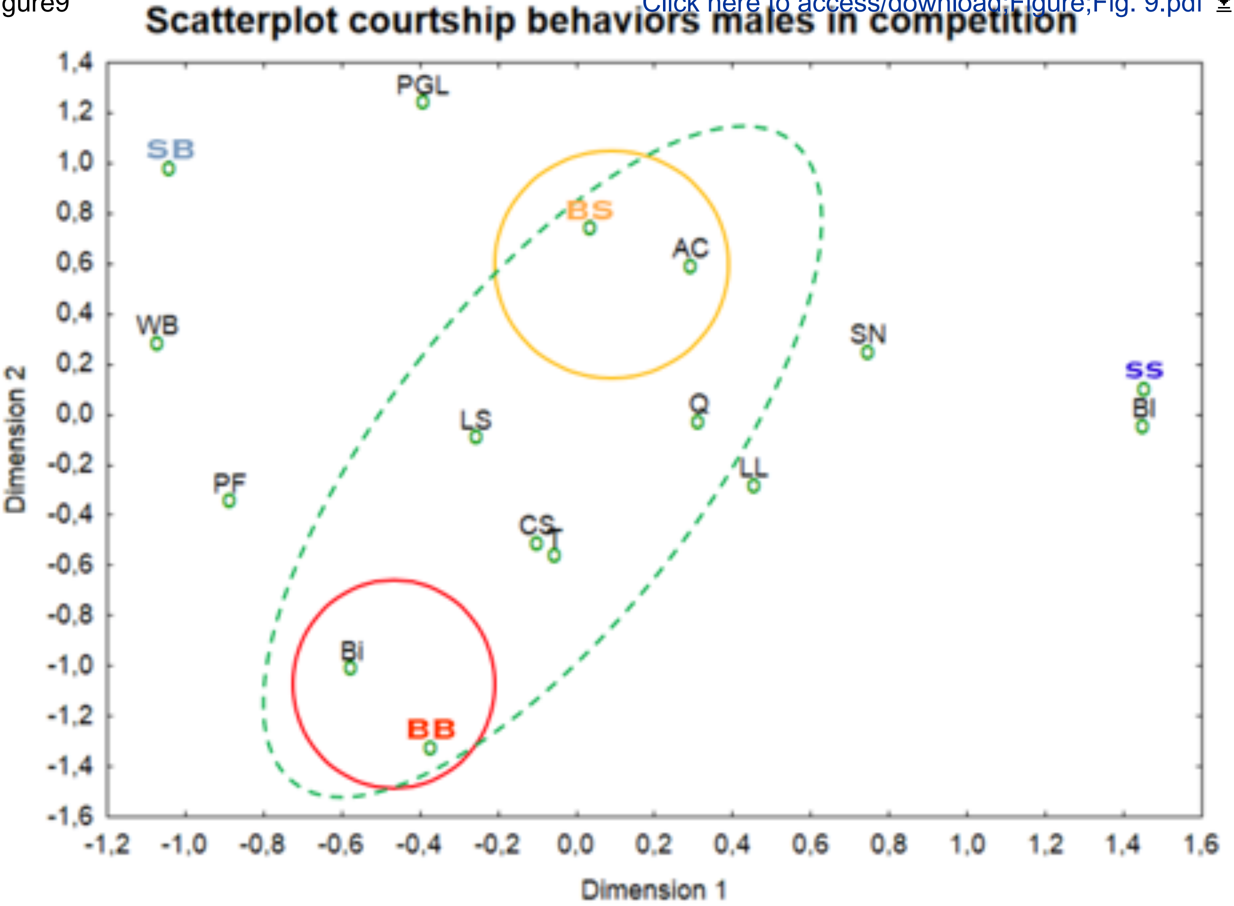

Click here to access/download
Supporting Information
S1_Video.mp4

Click here to access/download
Supporting Information
S2_Video.m4v

Click here to access/download
Supporting Information
S1_Table.docx

Click here to access/download
Supporting Information
S2_Table.docx

Click here to access/download
Supporting Information
S3_Table.docx

Click here to access/download
Supporting Information
S4_Table.docx

Click here to access/download
Supporting Information
S5_Table.docx

Click here to access/download
Supporting Information
S6_Table.docx

Click here to access/download
Supporting Information
S7_Table.docx

Click here to access/download
Supporting Information
S8_Table.docx

Temperament and sexual behaviour in the Furrowed Wood Turtle *Rhinoclemmys areolata*

Cassola FM [1], Henaut Y [1*], Cedeño-Vázquez JR [1], Méndez-de la Cruz FR[2], Morales-Vela B[1]

[1]El Colegio de la Frontera Sur, Unidad Chetumal, Chetumal, Quintana Roo, México

[2]Laboratorio de Herpetología, Departamento de Zoología, Instituto de Biología, Universidad Nacional Autónoma de México. México.

*Corresponding Author

e-mail: yhenaut@ecosur.mx

Abstract

Temperament variability in animals have consequences for evolution and ecology. One of the primary effects of consistent behavioral differences points to reproduction. In chelonians some works have focused on the study of temperament using different methods for its measurement. In our research we first wanted to i) establish a methodology to determine boldness degree, in the turtle *Rhinoclemmys areolata*. Secondly, we wanted to ii) determine the role boldness plays during reproduction, with emphasis on courtship and copula, considering a) the interactions between male and female, and b) the competition between males. We used 16 sexually mature individuals of each sex. We first observed the males in four different situations and recorded 17 behavioral traits. We selected 12 traits that allowed us to determine the bolder and the shier individuals. We found that five behavioral traits were specific for bolder individuals and five others for the shier. In a second step, we observed a male in presence of a female and recorded courtship behaviors and breeding attempts. Bolder individuals did not display courtship behaviors and just attempt to copulate. Shier individuals displayed courtship behaviors and rare copula attempts. Finally, in the simulations that compared two males in the presence of a female we noticed that bolder individuals showed courtship behaviors while the more shy ones simply ignored the female. Our results first allowed us to determine which methodology is the best to determine temperament in turtles. Secondly, the temperament seems to be an important factor to modulate interaction between males and females. Bolder individuals have an advantage during competition and display courtship behaviours only if other males are present. Shier males displayed courtship behaviors and try to copulate only when no competitors were present. Those two different temperament-dependant strategies are discussed in terms of ecology, evolution and management.

Introduction

Inter-individual behavioral differences in an animal species are consistent or largely maintained over time and in diverse situations [1] and sometimes between one population and the other [2]. These differences are what some scientists call “temperament”, “behavioral syndrome” or “reactivity”[3-4] and they have been attributed to the combined influences of genetic, social and environmental factors that affect the behavior of an individual [5].

Animal behavioral differences can have important implications for fitness [6-7] and have been shown to influence ecological processes such as dispersion, invasion, response to climate change and risk of extinction [8-10]. There is a high probability that temperament has a great influence on the way in which animals respond to new environments (for example, new exhibits or retention areas), to familiar and unfamiliar conspecifics, to strangers of other species, and to changes in their environment, affecting social compatibility, stability and success of a group [11]. Animal behavior and temperament can influence breeding in capacity by directly connecting to a successful match (success of coupling) or the number and viability of offspring produced (reproductive success) by the specimens [12-14]. Since temperament affects the way in which individuals react to difficult situations, it can be predicted how a male can compete, select and be compatible with a sexual partner. Therefore, variations in individual behavior are a source of information in the field of sexual selection and reproductive success [6,15].

Individuals may systematically differ in one or more temperament traits, such as boldness, aggressiveness, reactivity, sociability, exploration or activity levels [1]. A feature that has been extensively studied in the field of temperament is boldness defined as the inclination of individuals to explore their environment and take risks in a new situation [16]. Boldness

and shyness are considered as a continuum [17-18] at one extreme we find shier specimens and on the opposite side the bolder ones.

This spectrum seems to have an adaptive explanation in terms of costs and benefits, related to the type of response to a risk. In general, bolder individuals tend to sustain high reproductive success but at a potential cost for their survival, while shy individuals engage in opposite behaviors, giving primary importance to survival over reproductive productivity. This implies that temperament would allow to regulate survival with other needs of the species [17].

There are few studies addressing the relationship between success of couples and behavioral compatibility. Knowing these individual differences provides essential information for captivity management and animal welfare [11,17]. For example, it allows us to understand why some specimens have reproductive problems in captivity and why they fail to satisfy their reproductive potential, even if they are physically and physiologically in health [19]. A few years ago, in fact, specimens suitable for mating were chosen based on their hypothetical genetic compatibility [20-21], their state of health [22] and / or stages of life history [23-24]. However, couples who represent a good genetic combination do not necessarily produce offspring, and behavioral incompatibility is often cited for this failure [25-28]. One way in which animal temperament can influence mating probability is through the effects associated with behavioral compatibility the way individuals interact with each other [12]. When animals are paired for reproduction, some temperaments may be compatible, while others may be in conflict [13,29-30]. There are, for example, cases where behavioral similarity is the key to reproductive success, as in the case of zebra finches (*Taeniopygia guttata*) and cockatiels (*Nymphicus hollandicus*) [31-32] ; while, in other species, different or even opposite temperaments can reach high reproductive

performances as in the giant panda (*Ailuropoda melanoleuca)* and in the black rhino (*Diceros bocornis)* [12,25,33].

Recently, boldness has been studied in aquatic turtles. The recognition between males and the reduction of competitive interactions are related to the boldness of males [34]. They showed that the response to chemical stimuli of known or unknown males of the Mediterranian Turtle *Mauremys leprosa*, depends on the level of boldness, in particular the shiest males are those who avoid encountering other males, of the same or different species, as it could help stabilize social systems and reduce the frequency and intensity of aggressive encounters between males. Different methods are used to evaluate temperament in turtles. Reactivity level is measured using the straightening response, which is the time it takes for an individual to straighten up after being turned upside down on his carapace [35]. A similar method has been used to study anti-predator responses in the European pond turtle *Emys orbicularis* [36]. Another method consists to insert specimens in a new environment: here, the latency to move from an initial position in an arena was used to evaluate exploration in the red-eared slider turtle *Trachemys scripta* [37] and in the Eastern hermann *Eurotestudo boettgeri* [38]. The presentation of a threatening stimuli was also used to measure the reactivity and the effect of new objects on investigative behaviors to measure exploration in Agassiz´s desert tortoise (*Gopherus agassizii*) [14]. The boldness in the Eastern box turtle (*Terrapene carolina*) was evaluated using the time to emerge from the shell and the time to move after a short period of confinement [39]. Therefore, there are different methods applicable to turtles but none of the aforementioned studies tried to apply more than one of them to a single species. For this reason, creating a unique methodology for the study of boldness in turtles is still lacking.

Unfortunately, there is a lack of work addressing specifically how temperament may be related to the reproductive behavior of turtles, particularly if it influences male competition

behavior, courtship and coupling. Thus, the aims of this study were to (i) determinate temperament of *Rhinoclemmys areolata* and provide a method to determine the temperament in these species that can also be used for others terrestrial turtle species (ii) understand if its temperament could be a factor that may modulate the interaction between male and female, and (iii) between males during the competition for a female.

Materials and methods

Ethical statement

The investigation was carried out in compliance with the institutional ethical standards and norms in force. All the animals come from private loans of the city of Chetumal (Quintana Roo – Mexico). Verbal consent was requested from the owners, and it was explained how the turtles would be used and for what objective. The individuals on loan were destined exclusively for specific research purposes, respecting all known needs for animal welfare, and were returned to their owners at the end of the data collection.

We were in possession of the permit N ° SGPA / DGVS / 002491/18 issued by Secretaria de Medio Ambiente y Recursos Naturales (SEMARNAT) for the collection of this species. Experimental protocol was approved by Ethical Committee from the “El Colegio de la Frontera Sur”, Mexico.

Object of study

For the experiment, we used 16 males and 16 females of the furrowed wood turtle *Rhinoclemmys areolata*, all sexually mature. This species occurs in Yucatán Peninsula, Cozumel Island, Northern Guatemala, Belize, Northwestern Honduras, where it inhabits savannah, thorny scrub, broad-leaved forests, swampy and gouache areas [40]. It is used

as pet, food and in the traditional medicine [41]. It is listed as Near-threatened by the IUCN Red List [42]. It is also known as “Chak pool” or “Mojina” [41]. It is characterized by 1 to 2 red, orange or yellow stripes on the top of the head, and the plastron is yellow with a dark spot of variable size (Fig 1). It has a low level of sexual dimorphism, males having a longer and thinner tail than females, and also having a slightly more concave plastron but this is a characteristic that is not always detectable (Fig 2). It is a terrestrial species, whose only connection with the aquatic environment is during courtship and copulation; however, these derive from two anecdotic observations events where the specimens were kept in pools without the presence of a large terrestrial environment [43-44]. Courtship and copulation could be induced by spraying them with water [40].

Fig 1. Details of *Rhinoclemmys areolata* morphology.

Fig 2. Detail of female (left) and male (right). It can be seen the difference between the tails (red arrow), females have a shorter and thicker tail, while males have a longer and thinner one.

Turtles’ management and setup

Specimens of *Rhinoclemmys areolata* were kept within a 16 x 5 m enclosure, located in the facilities of El Colegio de la Frontera del Sur (ECOSUR) Unidad Chetumal (18 ° 32'37.4''N, 88 ° 15 ' 48.0''W). The area was fenced and divided into two parts of approximately the same size (5 x 8 m) containing male and female specimens, respectively. This division avoided any reproductive activity in the absence of the observer. It also avoids female from storing semen from males and be less reactive to males [45].

The area inside the fence was as close as possible to the natural habitat of *R. areolata*, and it was possible by maintaining the presence of trees, foliage, natural soil and adding water sites (Fig 3).

Turtles were fed with fruit, vegetables and meat on alternate days. Inside the maintenance area, they were free to move, to find refuge and bathe in small pools. They were subject to natural temperatures and rainfall for as long as they were inside the area in order to keep the best environmental conditions for their welfare.

All simulations were performed in a mobile enclosed area (2 x 2 m) (Fig 4). Each simulation was recorded through two cameras (YiLiteCam and Motorola Camera). The first video camera was positioned above the observation area while the second was moved manually by the observer in case details of the events were needed.

Each simulation took place in open air, on sunny days, during hours of maximum activity of turtles (8.30am-11.30am and 05.30pm - 06.30pm), when the temperatures are not very high. Animals were separated after each observation session to avoid any additional intersexual interactions in absence of the observer. The observation area was moved in an adjacent area after every simulation to reduce waiting time between experiments and recovery time of environmental conditions, then a new group of turtles was placed to repeat simulation.

Fig 3. Captive living area of *Rhinoclemmys areolate* at the ECOSUR campus.

Fig 4. Observation area from the side and from the top views.

Experiments

Determination of temperament of *Rhinoclemmys areolata*

It was necessary to identify stable behavioral traits over time and in different situations. For this reason, 16 males were observed individually and in isolation in four different situations:

- Reaction to manipulation: a male is taken from the confinement and handled briefly for 1 minute.
- Reaction to risk of predation: a male is taken from the confinement, handled briefly and gently released in an overturned position, that is, on his carapace.
- Reaction to a new environment: a male is taken from the confinement and released in experimental area unknown to the specimen. (30min)
- Reaction to the introduction of a new object: a male is taken from the confinement, released in experimental area and, after 5 minutes, an unknown object is suddenly released (stuffed dog-like animal). (30min)

To organize males in order to describe their boldness-shyness level we: (i) extrapolated behavioral events of each simulation from video recordings, (ii) converted them into variables, (iii) defined occurrence (=1) and absence (=0) of each event obtaining binomial values; (iv) defined frequency and (v) time in seconds when possible, obtaining quantitative values; (vi) for the latter, it was measured the moment of abrupt change of slope within data range, this way it was possible to classify male as shy or bold (S1 Table).

Each event was considered as a variable and inserted into an Excel sheet. A boldness index was given to each male, dividing the sum of “occurrences” of variables that could describe a bold temperament by the total number of variables. This analysis allowed us to classify specimens into groups, according to their degree of boldness.

Afterwards, variables were analysed using a correlation matrix and the obtained values were inserted into a multidimensional scaling (MDS) that allowed us to elaborate a map showing relative positions between different events and relative distances.

Finally, variables were analysed through a Mann-Whitney test to determine which ones were significant in determining temperament.

Before starting interaction experiments, we tested the influence of turtles' length (that is considered linked with the age of each individual) on temperament. We did this test to ensure results are not the consequence of the size or age of the turtles. The two groups obtained were analysed in relation to straight carapace length (SCL) and straight plastron length (SPL) through Mann-Whitney test (S2 Table).

Temperament as a modulating factor during the interaction between male and female

16 groups of turtles consisting of one male and one female were created randomly. Each couple was released, sequentially, within the experimental area, each individual inside a metal box. They were soaked with water using an artificial sprayer to promote reproductive behavior and later freed to move around for 30 minutes.

For this experiment, the same specimens were not used more than once.

Each group remained sequentially in the experimental arena and was carefully observed.

As for the first simulations, we (i) extrapolated behavioral events from each simulation, (ii) converted them into variables, (iii) defined occurrence (=1) and absence (=0) of each event obtaining binomial values; (iv) defined frequency and (v) time in seconds when possible, obtaining quantitative values; (vi) for the latter, it was measured the moment of abrupt change of slope within data range (S3 and S4 Tables).

Each event was considered as a variable and inserted into an Excel sheet. Variables were analysed using a correlation matrix and the obtained values were inserted into a multidimensional scaling (MDS) that allowed us to elaborate a map showing relative positions between different events and relative distances.

Temperament as a modulating factor during the competitive interaction between males in the presence of a female

Eight experimental groups were randomly created, each consisting of two males and one female. For this experiment, the same specimens were not used more than once.

The specimens were placed equidistant within the experimental area, soaked with water using an artificial sprayer to promote reproductive behavior and freed to move around for 30 minutes.

Each group remained in the experimental area and was carefully observed.

As for the first simulations, we (i) extrapolated behavioral events from each simulation, (ii) converted them into variables, (iii) defined occurrence (=1) and absence (=0) of each event obtaining binomial values; (iv) defined frequency and (v) time in seconds when possible, obtaining quantitative values; (vi) for the latter, it was measured the moment of abrupt change of slope within data range (S5 and S6 Table).

Each event was considered as a variable and inserted into an Excel sheet. Variables were analysed using a correlation matrix and the obtained values were inserted into a multidimensional scaling (MDS) that allowed us to elaborate a map showing relative positions between different events and relative distances.

Results

Determination of temperament of *Rhinoclemmys areolata*

We observed 17 behavioral traits (S1 Table) but several of them were displayed by all the turtles and were not useful to distinguish temperaments; therefore, we selected 12 behavioral traits that were used as variables (Table 1). For each male, the presence or

absence of each of them was defined (Table 2). For this study, we used “bolder” and “shier” terms instead of “bold” and “shy” since boldness is a continuous spectrum and it is not possible to create a sharp and fixed fractures for group definition.

In order to create two groups for the following experiments, we considered as shier all turtles that had an index less than or equal to 0.5, while bolder were those that had an index greater than 0.5. This allowed us to divide specimens into 9 bolder and 7 shier males (Table 2).

Straight carapace length and straight plastron length did not show a significant relationship with temperament (Carapace: Mann-Whitney U test: U=20, P=0.25; Plastron: Mann-Whitney U test: U=19, P=0.21) (S7 Table).

Rearranging the variables according to presence, frequency and times (Table 1), and through the multidimensional scaling, we obtained a total of 10 variables: five correlated with the shiest individuals and other five associated with the boldest individuals (Fig 5).

These variables were characteristic of the shier turtles: (i) hiding in a new environment, (ii) not crossing a new environment, (iii) high quiet time in a new environment, (iv) high straightening time during predation, and (v) low walking time in a new environment. Variables for boldest turtles were: (i) not hiding in a new environment, (ii) cross a new environment, (iii) low quiet time in a new environment, (iv) low straightening time during predation, and (v) high walking time in a new environment (Fig 5). Four of those variables were also statistically significant for the discrimination of the two groups (Fig 6, S8 Table).

Table 1. Variables selected for simulations, subdivision used in correlation analysis and multidimensional scaling with relative abbreviation.

Variable	Subdivision for analysis	Abbreviation
Kicking during manipulation (KM)	Fast kicking	FK

	Slow kicking	SK
Neck stretched during manipulation (NeSM)	Yes neck stretched manipulation	YNeSM
	No neck stretched manipulation	NoNeSM
Hiding inside shell during manipulation (HSM)	Yes hide shell manipulation	YHSM
	No hide shell manipulation	NoHSM
Neck retracted during manipulation (NeRM)	Yes neck retracted manipulation	YNeRM
	No neck retracted manipulation	NoNeRM
Straightening time during predation (STP)	High straightening time predation	HSTPr
	Low straightening time predation	LSTPr
Hiding inside shell during predation (HSP)	Yes hide shell predation	YHSPr
	No hide shell predation	NoHSPr
Walking time in a new environment (WTNE)	High walking time new environment	HWTNE
	Low walking time new environment	LWTNE
Hiding in a new environment (HNE)	Yes hide new environment	YHNE
	No hide new environment	NoHNE
Quiet time in new environment (QTNE)	High quiet time new environment	HQTNE
	Low quiet time new environment	LQTNE
Crossing a new environment (CrNE)	Yes cross area	YCrNE
	No cross area	NoCrNE
Exploration peluche (ExP)	Yes exploring peluche	YExP
	No exploring peluche	NoExP
Exploring time peluche (ExTP)	High time exploring peluche	HExTP
	Low time exploring peluche	LExTP

Table 2. Presence (=1) or absence (=0) of variables selected for simulation and boldness index values. In grey background color the specimens considered bolder, in white those considered shier.

Turtle Name	Sum variables	Boldness Index	KM	NeSM	HSM	NeRM	STD	HSPr	WTNE	HNE	QTNE	CrNE	ExP	ExTP
**Alfredo**	9	0,75	1	1	1	1	1	0	0	1	0	1	1	1
**Benedicto**	12	1	1	1	1	1	1	1	1	1	1	1	1	1
**Carlos**	8	0,67	1	1	1	1	1	0	0	1	1	1	0	0
**Hector**	8	0,67	1	1	1	0	1	0	1	1	1	0	1	0
**Nestor**	8	0,67	1	1	1	1	0	0	1	1	1	1	0	0
**Erik**	7	0,58	0	0	0	0	1	1	1	1	1	1	1	0
**Garry**	7	0,58	0	1	0	0	1	0	1	1	1	0	1	1
**John**	10	0,83	0	1	1	1	1	1	1	1	1	1	1	0
**Oliver**	8	0,67	1	0	1	0	1	0	1	1	1	1	1	0
**Francesco**	5	0,41	1	1	1	0	0	0	0	1	1	0	0	0
**Kevin**	3	0,25	0	0	1	0	0	0	0	0	0	0	1	1
**Marc**	5	0,41	1	1	1	1	0	0	0	1	0	0	0	0
**Patricio**	3	0,25	0	0	1	0	0	0	0	0	0	0	1	1
**Denis**	6	0,5	1	1	1	0	1	0	0	1	0	1	0	0
**Ian**	6	0,5	1	1	1	1	1	1	0	0	0	0	0	0
**Lorenzo**	5	0,41	1	0	1	0	1	1	0	0	0	0	1	0

Temperament as a modulating factor during the interaction between male and female

As results for the relation between temperaments and male and female interaction, we observed behaviors linked with courtship and others with mating.

Regarding courtship, we determined 16 behaviors (S3 Table), but we used 11 of them for the analysis, excluding behaviors showed by most of the specimens and the ones that did not show differences between the groups.

We defined if they occurred or not and how often to obtain their frequency (low or high) (Table 3). We observed that the following traits are linked with the shier individuals (Fig 7): (i) pushing the female, (ii) blocking the female, (iii) frontal approach with stretched legs and retracted neck (iv) pushing the ground with front leg, (v) approaching when excited. On the other hand, some bolder turtles did not show courtship behavior while in others they were not very frequent. Bolder individuals are linked with: (i) do not block the female, (ii) do not push it, (iii) do not approach excitedly, (iv) do not walk backwards, (v) low frequency of frontal approach with stretched legs and extended neck (Fig 7).

Four behaviors (Table 4, S4 Table) were observed for mating. None of those behaviors is displayed by shiest individuals, while the boldest are more linked with intent of mounts, and mount at high frequency (Fig 8, S1 Video).

Table 3. Courtship behaviors obtained from simulations male vs female, subdivision used in correlation analysis and must-dimensional scale with relative abbreviation.

Variable	Subdivision
Frontal approach with stretched legs and stretched neck	High Frequency (HFASLSN)<br>Low Frequency (LFASLSN)<br>No Approach (NoFASLSN)
Frontal approach with stretched legs and retracted neck	High Frequency (HFASLRN)<br>Low Frequency (LFASLRN)<br>No Approach (NoFASLRN)
Cross snouts	High Frequency (HCrS)<br>Low Frequency (LCrS)<br>No Cross (NoCrS)
Push ground with front leg	High Frequency (HPGL)<br>Low Frequency (LPGL)<br>No Push (NoPGL)
Block the female	Yes Blocking (YBlock)<br>No Blocking (NoBlock)
Pushing the female	Yes Pushing (YPush)<br>No Pushing (NoPush)
Walk backwards	High Frequency (HWB)<br>Low Frequency (LWB)<br>No Walk (NoWB)
Excited male is approaching	Yes excited male (YEMA)<br>No excited male (NoEMA)
Quiet male explores surroundings	Yes Explore (YQMExp)<br>No Explore (NoQMExp)
Cloaca sniffing	High Frequency (HCS)<br>Low Frequency (LCS)
Remote tracking	Yes Remote (YRT)<br>No Remote (NoRT)

Table 4. Mating behaviors obtained from simulations male vs female, subdivision used in correlation analysis and must-dimensional scale with relative abbreviation.

Variable	Subdivision
Neck stimulation	High Frequency (HNS)
	Low Frequency LNS)
	No Stimulation (NoNS)
Mount attempt	High Frequency (HMA)
	Low Frequency (LMA)
	No Mount attempt (NoMA)
Coitus	Yes Coitus (YCo)
	No Coitus (NoCo)
Mount	High Frequency (HM)
	Low Frequency (LM)
	No Mount (NoM)

Temperament as a modulating factor during the competitive interaction between males in the presence of a female

We obtained 12 behaviors related to courtship (Table 5, S5 Table). We considered temperament of the two males together with a female and observed that courting variables are concentrated around the boldest regardless the temperament of their opponent (Fig 9). On the contrary, the shiest did not display particular behaviors except for blocking the female during the tracking (Fig 9) (S2 Video).

Table 5. Courtship behaviors obtained from simulations male vs male vs female used in correlation analysis and must-dimensional scale with relative abbreviation.

Variable	Abbreviation
Approaching to cloaca	AC
Stretching neck towards female	SN
Cloaca sniffing	CS
Lateral sniffing	LS
Tracking	T
Quiet alongside female	Q
Bite	Bi
Blocking female	Bl
Lean leg to female	LL
Walk backwards	WB
Push female	PF
Push ground with leg	PGL

Fig 5. Distribution of the variables (behaviors) of the specimens. The red circle shows variables linked with the boldest specimens, the blue circle identifies behaviors linked to the shiest ones.

Fig 6. Mean values and standard errors of the four significant binomial variables. High time walking new environment, Bolder: mean±SE = 0,78±0,1, Shier: mean±SE= 0±0, Mann-Whitney U test: U=7, p=0.00*3; Not hiding new environment, Bolder: mean±SE = 1±0, Shier: mean±SE= 0,43±0,2, Mann-Whitney U test: U=13,5, p=0.013*; Low time quiet new

environment, Bolder: mean±SE = 0,89±0,1, Shier: mean±SE= 0,14±0,14, Mann-Whitney U test: U=37, p=0.004*; Cross new environment, Bolder: mean±SE = 0,78±0,14, Shier: mean±SE= 0,14±0,1, Mann-Whitney U test: U=37, p=0.017*.

Fig 7. Distribution of courtship variables (behaviors) of the male specimens. The red curve includes variables linked with boldest specimens, the blue curve congregates behaviors linked with the shiest individuals.

Fig 8. Distribution of mating variables (behaviors) of the male specimens. The red curve encompasses variables linked with boldest individuals, the blue curve includes behaviors linked with the shiest ones.

Fig. 9. Distribution of courtship variables (behaviors) of the specimens. The red circle delimits variables highly linked with boldest males in competition with each other, the yellow circle encompasses those highly linked with a bold male in competition with a shy one, the green ellipse includes behaviors linked with the boldest not considering who is its competitor.

Discussion

A variety of methods were used but separately to determine temperament in aquatic and terrestrial turtles [4,35-39]. This is the first paper that tests all of them. In this study, we tested reaction to: manipulation, predation risk, a new environment and the introduction of a novel object with the purpose of looking for consistency, to determine the most accurate behaviors and in consequence the best method to define the level of boldness in turtles. Behaviors observed in a new environment seem to be the best to determine the boldness in turtles: hiding and limited walk for the shiest, being quiet (shiest) or not (boldest), walking

a lot and crossing the new environment for the boldest. These results coincide with two of previous works [37-38].

The test with the risk of predation also shows that the time to straighten is linked with the boldness as observed in previous studies [35-36].

Our work focused only on males, and the determination of temperament in females or juveniles may be different even if the exploration of a new environment may be equivalent whatever the sex and age of turtles that generally share the same strategy of life whatever their conditions. Therefore, to assure that behaviors linked with the reaction to a new environment can be a better method, we suggest further studies to test it also in females and juveniles.

An interesting element that emerges in this study is the modulation of males´ behavior. When a bold male is alone with a female, he often skips courtship behaviors to devote himself to stimulating the female for mounting and mating; when the same male is found in the presence of another conspecific male, a potential sexual competitor, the first engages in courtship behaviors towards the female. A similar result has been observed in the common water strider *Aquarius remiges* (Gerridae, Insecta), where the most active and aggressive males spend most of their time looking for a female as well as having the highest copula rates [46], and in the common lizard *Zoocora vivipara* (Lacertidae, Reptilia), where females prefer the more active males when they find themselves alone and without the presence of predators in their surroundings, whereas when shier specimens find themselves alone in the presence of a female, they spend time and energy in courtship behaviour [47]. In our simulations, mating attempts of shier males were very rare; when the shier is in presence of a competitor and a female, it ignores the female. In summary, boldest individuals court the female only in presence of a potential competitor and directly mate with

her without courtship if no competitor is present. Shier males courtship the female only if no competitor is present and is not very effective at mating. This kind of behavior towards a competitor was observed in *Mauremys leprosa* where responses to chemicals cues depended on boldness; in particular, shy turtles avoided chemical cues from familiar and unfamiliar bolder males [35]. However, it is possible for shier animals that more time is necessary to display a mating behavior, anyway our work shows apparent differences in the sexual behavior according to males' temperaments. Therefore, we can argue that the degree of boldness can influence males' reproductive behavior, in decision making and their reaction according to the situation. In terms of costs and benefits, boldness can be considered a factor that allows specimens to evaluate how to act during courtship and mating considering the presence of conspecifics of both sexes. On the other hand, it avoids competition and the possibility of losing energy when a bolder male is competing. In other species, male competition could be very costly because of fights, injuries, territory care and predatory danger during courtship and refraining those costs could also be an alternative strategy to avoid damages and predators, as shown in the Iberian wall lizard *Podarcis hispanica,* which uses chemical cues during competitive matches to recognize competitors and reduce intensity and costs of the fights [48]. The success of different strategies dependant of the temperament is under environmental constraint such as the presence of predators as observed for other animals [1]. For example, the broadhead skink *Eumeces* (*Plestiodon*) *laticeps* shows a sensitivity in predation risk during reproductive opportunities: when females were present, isolated and mate-guarding males initiate courtship, but isolated males permitted closer approach by a predator than did mate-guarding males. Moreover, there is a high level of latency by isolated males when a predator is close to the female so they were able to balance risk of predation with courtship behavior [49].

Researchers have already discussed that behavior plays a key role in animal survival and evolution, behavioral traits can help species to adapt to rapid changes in the environment [50], and survive to natural selection [51] as much as morphological and physiological traits. Differences in sexual behavior related to boldness or other behavioral traits, can be an honest signal of mate quality [52]. Therefore, identifying those behavioral traits that can affect population viability is important to provide helpful information to integrate conservation plans [53].

The information obtained in this study represents an important tool for the conservation of reptiles and in particular for land turtles. Worldwide, turtles represent one of the vertebrate groups with the highest risk of extinction [54]. In recent years, many researchers have ensured the importance of not only increasing the behavioral information of the species, but also implementing these data in their conservation and monitoring strategies [11,55].

To our best knowledge, this is the first work that connects temperament to reproductive behaviors in land turtles. What we have achieved can be used as a first step for compatibility plans for turtles, in order to combine temperament traits that might compose a successful breeding couple [11].

Temperament studies in couple compatibility have significant implications in *ex situ* conservation programs, since specific traits (such as aggressiveness and fear) have been related to reproductive success in some species raised in zoos [12]. In addition, certain temperaments may be more likely to thrive in captive environments, artificial selection for or against specific temperament traits may accelerate domestication processes, making animals raised for conservation less suitable for release in wildlife [26].

Our research showed that bolder males need less time to mount and mate, with perhaps higher fitness than shier ones.

Another application of our findings is the possibility to keep the whole range of boldness. Considering behavioral variation, it is important to preserve boldness since it allows conservationists to reintroduce animals with all the behavioural traits, giving them proper tools to survive in novel environments [51]. Considering this, in order to not to lose the behavioral pool during captive reproduction, we must be aware of the needs of different specimens. Therefore, our study has allowed to understand demonstrates that shy turtles likely need more time for mating and need to be alone with the female, otherwise they lose interest. For the boldest animals, the necessary times are lower and they are potentially able to reproduce even with the presence of conspecifics.

Acknowledgments

We would like to thank El Colegio de la Frontera del Sur and the CONACYT-OEA-AMEXCID scholarship program for the funds needed to cover expenses for data collection and specimen maintenance; the citizens of Chetumal who gave and loaned specimens of *R. areolata* for this research; José Alberto García Angulo for his help in the design and construction of the turtle maintenance and observation areas; Dr. Gabriele Gheza, Martina Anna Belli and Antonin Mareš for their help in grammatical and linguistic revision.

References

[1] Réale D, Reader S, Sol D, McDougall PT, Dingemanse NJ. Integrating animal temperament within ecology and evolution. Biol Rev. 2007;82: 291-318.

[2] Pervin L, John OP. Handbook of personality: theory and research. 2nd ed. New York: Guilford; 1999.

[3] MacKay JRD, Haskell MJ. Consistent individual behavioral variation: the difference between temperament, personality and behavioral syndromes. Animals. 2015;5: 455-478.

[4] Germano JM, Nafus MG, Perry JA, Hall DB, Swaisgood RR. Predicting translocation outcomes with personality for desert tortoises. Behav Ecol. 2017;28: 1075-1084.

[5] Dingemanse NJ, Araya-Ajoy, YG. Interacting personalities: behavioural ecology meets quantitative genetics. Trends Ecol Evol. 2015;31: 88-97.

[6] Dingemanse NJ, Réale D. 2005. Natural selection and animal personality. Behavior. 2005;142: 1165-1190.

[7] Smith BR, Blumstein DT. Fitness consequences of personality: a meta-analysis. Behav Ecol. 2008;19: 448-455.

[8] Chapman JW, Klaassen RH, Drake VA, Fossette S, Hays GC, Metcalfe JD, Reynolds AM, Reynolds DR, Alerstam T. Animal orientation strategies for movement in flows. Curr Biol. 2011;21: R861-R870.

[9] Duckworth RA, Badyaev AV. Coupling of dispersal and aggression facilitates the rapid range expansion of a passerine bird. Proc Natl Acad Sci. U.S.A. 2007;104: 15017-15022.

[10] Dyer JRG, Croft DP, Morrell LJ, Krause J. Shoal composition determines foraging success in the guppy. Behav Ecol. 2009;20: 165-171.

[11] Tetley CL, O'Hara SJ. Ratings of animal personality as a tool for improving the breeding, management and welfare of zoo mammals. Anim Welf. 2012;21: 463-476.

[12] Martin-Wintle MS, Shepherdson D, Zhang G, Huang Y, Luo B, Swaisgood RR. Do opposites attract? Effects of personality matching in breeding pairs of captive giant pandas on reproductive success. Biol Conserv. 2017;207: 27-37.

[13] Kelleher SR, Silla AJ, Byrne PG. Animal personality and behavioral syndromes in amphibians: a review of the evidence, experimental approaches, and implications for conservation. Behav. Ecol Sociobiol. 2018;72: 79.

[14] Gowaty PA, Drickamer LC, Schmid-Holmes S. Male house mice produce fewer offspring with lower viability and poorer performance when mated with females they do not prefer. Anim Behav. 2003;65: 95-103.

[15] Ah-King M, Gowaty PA. A conceptual review of mate choice: stochastic demography, within-sex phenotypic plasticity, and individual flexibility. Ecol. Evol. 2016;6: 4607-4642.

[16] Wilson ADM, Godin J-GJ. Boldness and behavioral syndromes in the bluegill sunfish, *Lepomis macrochirus*. Behav Ecol. 2009;20: 231-237.

[17] Cole EF, Quinn JL. Shy birds play it safe: personality in captivity predicts risk responsiveness during reproduction in the wild. Biol. Lett. 2014;10: 20140178.

[18] White SJ, Pascall DJ, Wilson AJ. Towards a comparative approach to the structure of animal personality variation. Behav Ecol. 2020;31: 340-351.

[19] Powell D, Lin H, Carlstead K, Kleiman D, Zhang H, Zhang G, et al. Relationships between temperament, husbandry, management, and socio-sexual behavior in captive male and female giant pandas (*Ailuropoda melanoleuca*). Acta Zool Sin. 2008;54: 169-175.

[20] Schwartz MK. Guidelines on the use of molecular genetics in reintroduction programs. In: The EU LIFE-Nature Projects to guidelines for the reintroduction of threatened species. March 21-22; Caramanico Terme, Italy; 2005. pp 51-58.

[21] Miller W, Wright SJ, Zhang Y, Schuster SC, Hayes VM. Optimization methods for selecting founder individuals for captive breeding or reintroduction of endangered species. In Biocomputing 2010. pp 43-53.

[22] Saidenberg ABS, Zuniga E, Melville PA, Salaberry S, Benites NR. Health-screening protocols for vinaceous amazons (*Amazona vinacea*) in a reintroduction project. J Zoo Wildl Med. 2015;46: 704-712.

[23] Kleiman DG. Foreword: animal behaviour studies and zoo propagation programs. Zoo Biol. 1994;13: 411-412.

[24] Bremner-Harrison S, Prodohl PA, Elwood RW. Behavioural trait assessment as a release criterion: boldness predicts early death in a reintroduction programme of captive-bred swift fox (*Vulpes velox*). Anim Conserv Forum. 2004; 313-320.

[25] Carlstead K, Mellen J, Kleiman DG. Black rhinoceros (*Diceros bicornis*) in US zoos: I. Individual behavior profiles and their relationship to breeding success. Zoo Biol. 1999a;18: 17-34.

[26] McDougall PT, Réale D, Sol D, Reader SM. Wildlife conservation and animal temperament: causes and consequences of evolutionary change for captive, reintroduced, and wild populations. Anim Conserv. 2006;9: 39-48.

[27] Freeman EW, Guagnano G, Olson D, Keele M, Brown JL. Social factors influence ovarian acyclicity in captive African elephants (*Loxodonta africana*). Zoo Biol. 2009;28: 1-15.

[28] Lees CM, Wilcken J. Sustaining the ark: the challenges faced by zoos in maintaining viable populations. Int Zoo Yearb. 2009;43: 6-18.

[29] Beach FA and Leboeuf BJ Coital behaviour in dogs. I. Preferential mating in the bitch. Anim Behav. 1967;15: 546-548.

[30] Sánchez-Macouzet O, Rodríguez C, Drummond H. Better stay together: pair bond duration increases individual fitness independent of age-related variation. Proc R Soc B. 2014;281: 20132843.

[31] Schuett W, Dall SR, Royle NJ. Pairs of zebra finches with similar ‘personalities’ make better parents. Anim Behav. 2011;81: 609-618.

[32] Rangassamy M, Dalmas M, Féron C, Gouat P, Rödel HG. Similarity of personalities speeds up reproduction in pairs of a monogamous rodent.Anim Behav. 2015;103: 7-15.

[33] Carlstead K, Fraser J, Bennett C, Kleiman DG. Black rhinoceros (*Diceros bicornis*) in US zoos: II. Behavior, breeding success, and mortality in relation to housing facilities. Zoo Biol. 1999b;18: 35-52.

[34] Ibáñez A, Polo-Cavia N, López P, Martín J. Honest sexual signaling in turtles: experimental evidence of a trade-off between immune response and coloration in red-eared sliders *Trachemys scripta elegans*. Naturwissenschaften. 2014;101: 803-811

[35] Ibáñez A, Marzal A, López P, Martín J. Boldness and body size of male Spanish terrapins affect their responses to chemical cues of familiar and unfamiliar males. Behav. Ecol Sociobiol. 2013;67: 541-548.

[36] Ibáñez A, Martín J, Gazzola A, Pellitteri-Rosa D. Freshwater turtles reveal personality traits in their antipredatory behaviour. Behav Process. 2018;157: 142-147.

[37] Carter AW, Paitz RT, McGhee KE, Bowden RM. Turtle hatchlings show behavioral types that are robust to developmental manipulations. Physiol Behav. 2016;155: 46-55.

[38] Mafli A, Wakamatsu K, Roulin A. Melanin-based coloration predicts aggressiveness and boldness in captive Eastern hermann's tortoises. Anim Behav. 2011;81: 859-863.

[39] Kashon EAF, Carlson BE. Consistently bolder turtles maintain higher body temperatures in the field but may experience greater predation risk. Behav. Ecol Sociobiol. 2018;72: 9.

[40] Vogt RC, Platt, SG, Rainwater TR. *Rhinoclemmys areolata* (Duméril and Bibron 1851), Furrowed wood turtle, Black-bellied turtle, Mojina. Chelonian Res. Monogr. 2009;5: 22-27.

[41] Cupul Cicero V, Aguilar Cordero WDJ., Chablé Santos J, Sélem Salas CI. Conocimiento etnozoológico de la herpetofauna de la comunidad maya de Santa Elena, Yucatán, México. Estud Cult Maya. 2019;54: 285-314.

[42] van Dijk PP, Lee J, Calderón Mandujano R, Flores-Villela O, Lopez-Luna MA. *Rhinoclemmys areolata*. *The IUCN Red List of Threatened Species. 2007.*

[43] Pérez-Higareda G, Smith HM. Courtship behavior in *Rhinoclemmys Areolata* from western Tabasco, Mexico (Testudines: *Emydidae*). The Gt. Basin Nat. 1988;48: 263-266.

[44] Artner H. Field observations and captive breeding of the furrowed wood turtle *Rhinoclemmys areolata* (Duméril and Bibron, 1851). Emys. 2009;16: 4-19.

[45] Pearse DE, Avise JC. Turtle mating systems: behavior, sperm storage, and genetic paternity. J Hered. 2001;92: 206-211.

[46] Sih, A, Bell, A, Johnson CJ, Ziemba RE. Behavioral syndromes: an integrative overview. Q Rev Biol. 2004;79: 241-277.

[47] Teyssier A, Elvire Bestion E, Richard M, Cotea J. Partners' personality types and mate preferences: predation risk matters. Behav Ecol. 2014;25: 723-733.

[48] López P, Martín J. Chemical rival recognition decreases aggression levels in male Iberian wall lizards, *Podarcis hispanica*. Behav Ecol. Sociobiol. 2002;51: 461-465.

[49] Cooper Jr. WE. Tradeoffs between courtship, fighting, and antipredatory behavior by a lizard, *Eumeces laticeps*. Behav Ecol Sociobiol. 1999;47: 54-59.

[50] Angeloni L, Crooks KR, Blumstein DT. Breed MD, Moore J, editors. Conservation and behavior: Introduction. 2010. pp. 377–381. Encyclopedia of animal behavior, Volume 1. Oxford: Academic Press.

[51] Caro T, Sherman PW. Endangered species and a threatened discipline: behavioural ecology. Trends Ecol Evol. 2011;26: 111–118.

[52] Merrick MJ, Koprowski JL. Should we consider individual behavior differences in applied wildlife conservation studies? Biol Conserv. 2017;209: 34-44.

[53] Ahtiainen JJ, Alatalo RV, Mappes J, Vertainen L. Decreased sexual signalling reveals reduced viability in small populations of the drumming wolf spider *Hygrolycosa rubrofasciata*. Proc R Soc London B. 2004;271: 1839-1845.

[54] Rhodin AG, Stanford CB, Van Dijk PP, Eisemberg C, Luiselli L, Mittermeier RA, et al. Global conservation status of turtles and tortoises (Order *Testudines*).Chelonian Conserv Biol. 2018;17: 135-161.

[55] Tadesse SA. Animal behavior in conservation biology. Intl J Avian Wildl Biol. 2018;3: 25-26.

Supporting information

Table S1. List of responses found in behavioral simulation (occurrence=1, absence=0), with relative abbreviation and subdivision applied for statistical analysis. In the first column, there are names of each specimen, in grey background color the bolder and in white the shier.

S2 Table. Straight carapace and straight plastron length measurements of each male turtle. In grey background color the specimens considered bolder, in white those considered shier.

S3 Table. List of courtship behaviors during male and female simulation (occurrence=1, absence=0), with relative abbreviation and subdivision applied for statistical analysis. In the first column, there are names of each specimen, in grey background color the bolder and in white the shier.

S4 Table. List of mating behaviors during male and female simulation (occurrence=1, absence=0), with relative abbreviation and subdivision applied for statistical analysis.

In the first column, there are names of each specimen, in grey background color the bolder and in white the shier.

S5 Table. List of courtship behavior during male vs male competition in presence of female (occurrence=1, absence=0), with relative abbreviation and subdivision applied for statistical analysis. In the first column, there are names of each specimen, in red the bolder and in blue the shier. Columns 2,3,4 and 5 indicate pairs by temperament (BB=boldVSbold, BS=boldVSshy, SS=shyVSshy, SB=shyVSbold).

S6 Table. List of mating behavior during male vs male competition in presence of female (occurrence=1, absence=0), with relative abbreviation and subdivision applied for statistical analysis. In the first column, there are names of each specimen, in grey background color the bolder and in white the shier. Columns 2,3,4 and 5 indicate pairs by temperament (BB=boldVSbold, BS=boldVSshy, SS=shyVSshy, SB=shyVSbold).

S7 Table: Statistical results of Mann-Whitney test on length measurements.

S8 Table: Statistical results of Mann-Whitney test on variables selected for boldness index, significant variables are indicated by *.

S1 Video. Shy courtship and Bold mount.

S2 Video. Shy and bold male in presence of a female. Shy is walking around the arena while the bold courtships the female.

A) **Editor comments (changes in green in the document)**

1) We revised Plos One style requirements.
2) To answer the editor comments about ethical statements (points 2, 3, 4, 5, 6), in the ethical declaration we added the following paragraph:

“All the animals come from private loans of the city of Chetumal (Quintana Roo – Mexico). Verbal consent was requested from the owners, and it was explained how the turtles would be used and for what objective. The individuals on loan were destined exclusively for specific research purposes, respecting all known needs for animal welfare, and were returned to their owners at the end of the data collection.

We were in possession of the permit N ° SGPA / DGVS / 002491/18 issued by Secretaria de Medio Ambiente y Recursos Naturales (SEMARNAT) for the collection of this species.”

3) Page 18 line 325: in Materials and Methods we corrected the mistake in the table caption, it was a typing error.
4) Pages 30-32 lines 581-620: We added the captions for supporting information files.
5) We revised all the references and updated all the new citations.

B) **Responses to Reviewer (in yellow in the document):**

Short Title

6) Page 1 Line 10: we deleted the short tittle after we revised Plos One style requirements (see Editor comments).

Summary

7) Page 2 Lines 29-31: According to the reviewer comment, we changed the phrase "Finally, we observed two males in presence of one female and noticed that bolder individuals then displayed courtship behaviors while the shier just ignore the female." with "Finally, in the simulations that compared two males in the presence of a female we noticed that bolder individuals showed courtship behaviors while the more shy ones simply ignored the female." The change was made because the result does not come from a single anecdote as the previous sentence suggested, but from multiple individuals and repetitions.
8) Page 2 Lines 34-35: following the previous correction this sentence should now coincide with results from multiple experiments and not just one, as it previously seemed.
9) Page 2 line 48: we deleted the Key Word after we revised Plos One style requirements (see Editor comments).

Introduction

10) Page 3 line 44-45: as suggested by the reviewer we suppressed the term personality considered as anthropomorphic and controversial. Also, as suggested, we mentioned now the term "reactivity" and suppressed "permanently".
11) Page 3 line 54: we changed "breed" with "breeding" as suggested by the reviewer.
12) Page 3 line 56: as suggested by the reviewer we added the citation "Gowaty PA, Drickamer LC, Schmid-Holmes S. Male house mice produce fewer offspring with lower viability and poorer performance when mated with females they do not prefer. Anim. Behav. 2003;65: 95-103".

13) Page 3 line 59: as suggested by the reviewer we added the citation "Ah-King, M., & Gowaty, P. A. (2016). A conceptual review of mate choice: stochastic demography, within-sex phenotypic plasticity, and individual flexibility. *Ecology and evolution*, *6*(14), 4607-4642".

14) Page 4 line 69: as suggested by the reviewer, we added "giving primary importance to survival over reproductive productivity" to specify behaviors.

15) Page 4-5 lines 85-90: as suggested by the reviewer we suppressed the unnecessary sentence and add references (Beach FA and Leboeuf BJ 1967, Coital behaviour in dogs. I. Preferential mating in the bitch. Animal behaviour, 15(4) 546-548, Sánchez-Macouzet O, Rodríguez C, Drummond H. 2014 Better stay together: pair bond duration increases individual fitness independent of age-related variation. Proc. R. Soc. B 281: 20132843).

16) Page 4-5 lines 85-90: As suggested by the reviewer in the discussion, we added the following paragraph in the introduction "There are, for example, cases where behavioral similarity is the key to reproductive success, as in the case of zebra finches (*Taeniopygia guttata*) and cockatiels (*Nymphicus hollandicus*) [31-32] ; while, in other species, different or even opposite temperaments can reach high reproductive performances as in the giant panda (*Ailuropoda melanoleuca)* and in the black rhino (*Diceros bocornis)* [12, 25, 33]."

17) Page 5 line 91: we created a new sentence as suggested by the reviewer deleting "and the study shows that"

18) Page 5 line 98: as suggested by the reviewer we changed boldness for reactivity.

19) Page 5 line 105: as suggested by the reviewer we changed boldness for reactivity.

20) Pace 5 line 110: we changed "attempt to create" with "creating" as suggested by the reviewer.

Material and Methods

21) Page 6 line 125: we deleted the hyphenation in “North-western”.

22) Page 7 line 146: we change the tense of “avoid” to “avoided”.

23) Page 7 line 147: we were talking about sperm storage that was studied in different turtles’ species but there aren’t any available work about this species. For a better understanding we changed the word “recollecting” with “storing”.

24) Page 8 line 167: during the trial we used water only to spraying the individuals, no pool or drinking point were used, therefore there was no reason to refill something. The water we used to spray was always new and after every trial we changed the position of the observation area into a dry one.

25) Page 10 line 228: we changed the tense from “will not be” to “were”.

26) Page 10 line 232: We used “sequentially” to explain that the experiments were done one after the other, but the term is maybe not adequate. We deleted this word to avoid confusion.

Results

27) Page 12 line 262: we changed the word “organisms” with "turtles” as suggested by the reviewer.

28) Page 12 line 265: we changed “didn’t” with “did not” as suggested by the reviewer.

29) Page 12 line 273: as suggested by the reviewer we change “for the shier” by “characteristic of the shier turtles”.

30) Page 12 line 276: As suggested by the reviewer we changed “for the boldest” by “. Variables for the boldest turtles were”.

Discussion

31) Page 20 line 361: we changed the beginning of the sentence from “Also, our” to “Our” as suggested by the reviewer.

32) Page 21 line 386: we deleted “the”.

33) Page 21 line 386: we added “animals”.

34) Page 21 line 393-394: according to referee’s commentary, we add in the sentence that it was observed for other species “In other species, male competition could be very costly”.

35) Page 22 lines 407-408: in order to rewrite the sentence as suggested by the reviewer, we changed “respond” with “adapt” and we delete “pass under the clutches of” to add “survive to”.

36) Page 22 line 418: according to the reviewer comment we suppressed” behavioral trait” and added “temperament” to be more specific in our affirmation.

37) Page 22: we deleted the paragraph “In the study of Spoon *et al* (2006), it can be seen that compatible pairs (male-female) of the Cockatiel *Nymphicus hollandicus* showed higher levels of coordination in parental care and raised more chicks; another example is given by Carlstead *et al* (1999a, b) who observed in Black Rhino *Diceros bocornis* that smaller enclosures determined the breeding of dominant males, while domination decreased in males housed in larger enclosures. In addition, dominance levels were related to reproductive success, with the conclusion that the number of births per year was higher in pairs of rhinos composed of a submissive male and a dominant or aggressive female. This information gives an idea of the optimal conditions for the breeding of captive rhinos, including large enclosures, which facilitate submissive behavior in males, which in turn affects the compatibility of couples; in the giant panda

Ailuropoda melanoleuca, Martin-Wintle *et al* (2017) conducted behavioral studies to further improve reproductive performance and found that specific combinations of temperament traits showed better reproductive performance than others. Males who were more aggressive than their partners were more likely to mate and produce puppies than when the female had a higher level of aggression than the male or even couples with less fearful males, regardless of the fear of the female, obtained better reproductive results. The application of these results to captive breeding management strategies should result in higher reproductive rates and the production of more candidates for the giant panda reintroduction program." and added a resumed version in page 4 lines 95-100.

38) Page 22 lines 428: we deleted "focused on boldness degree of males", same with "therefore they could have a" and added "with perhaps".

39) Page 23 lines 435 and 437: we deleted "has allowed to understand" and added "demonstrates" and added "animals".

40) Page 23: as suggested by the reviewer and to avoid repetition of the same idea, we deleted the sentence "By another way in term of evolution, being shiest may have some advantages and, if not for reproduction, they may be for antipredatory behaviour, making shiest individuals, that are more prudent in term of exploration, and as we observed in the new environment, generally less predated than boldest individuals as discussed for other species."

41) Page 24: as suggested by the reviewer we deleted the last paragraph.

Bibliography

42) Following the suggestions of the reviewer we made changes in the citations. We added the following works:

- Gowaty PA, Drickamer LC, Schmid-Holmes S. Male house mice produce fewer offspring with lower viability and poorer performance when mated with females they do not prefer. Anim. Behav. 2003;65: 95-103.
- Ah-King M, Gowaty PA. A conceptual review of mate choice: stochastic demography, within-sex phenotypic plasticity, and individual flexibility. Ecol. Evol. 2016;6: 4607-4642.
- Beach FA and Leboeuf BJ Coital behaviour in dogs. I. Preferential mating in the bitch. Anim Behav. 1967; 15: 546-548.
- Sánchez-Macouzet O, Rodríguez C, Drummond H. Better stay together: pair bond duration increases individual fitness independent of age-related variation. Proc. R. Soc. B. 2014; *281*: 20132843.

And deleted:

- Cable RN. Oh, Behave: When wildlife behavior matters in Conservation. Mich. J. Sustain. 2013;1
- Cooper Jr WE. Variation in escape behavior among individuals of the striped plateau lizard *Sceloporus virgatus* may reflect differences in boldness. J. Herpetol. 2009;43:495-502.
