# Supplementary material for: Temperament and sexual behaviour in the Furrowed Wood Turtle Rhinoclemmys areolata
Source: PLoS One. 2020 Dec 30;15(12):e0244561. doi: 10.1371/journal.pone.0244561 (PMC7773281; doi:10.1371/journal.pone.0244561)
Supplement: Attachment — Submitted filename: Response to Reviewers.docx [file pone.0244561.s013.docx]

A) **Editor comments (changes in green in the document)**
1) We revised Plos One style requirements.
2) To answer the editor comments about ethical statements (points 2, 3, 4, 5, 6), in the ethical declaration we added the following paragraph:

**“**All the animals come from private loans of the city of Chetumal (Quintana Roo – Mexico). Verbal consent was requested from the owners, and it was explained how the turtles would be used and for what objective. The individuals on loan were destined exclusively for specific research purposes, respecting all known needs for animal welfare, and were returned to their owners at the end of the data collection.

We were in possession of the permit N ° SGPA / DGVS / 002491/18 issued by Secretaria de Medio Ambiente y Recursos Naturales (SEMARNAT) for the collection of this species.”

3) Page 18 line 325: in Materials and Methods we corrected the mistake in the table caption, it was a typing error.
4) Pages 30-32 lines 581-620: We added the captions for supporting information files.
5) We revised all the references and updated all the new citations.
B) **Responses to Reviewer (in yellow in the document):**

**Short Title**

6) Page 1 Line 10: we deleted the short tittle after we revised Plos One style requirements (see Editor comments).

**Summary**

7) Page 2 Lines 29-31: According to the reviewer comment, we changed the phrase “Finally, we observed two males in presence of one female and noticed that bolder individuals then displayed courtship behaviors while the shier just ignore the female.” with “Finally, in the simulations that compared two males in the presence of a female we noticed that bolder individuals showed courtship behaviors while the more shy ones simply ignored the female.” The change was made because the result does not come from a single anecdote as the previous sentence suggested, but from multiple individuals and repetitions.
8) Page 2 Lines 34-35: following the previous correction this sentence should now coincide with results from multiple experiments and not just one, as it previously seemed.
9) Page 2 line 48: we deleted the Key Word after we revised Plos One style requirements (see Editor comments).

**Introduction**

10) Page 3 line 44-45: as suggested by the reviewer we suppressed the term personality considered as anthropomorphic and controversial. Also, as suggested, we mentioned now the term “reactivity” and suppressed “permanently”.
11) Page 3 line 54: we changed “breed” with “breeding” as suggested by the reviewer.
12) Page 3 line 56: as suggested by the reviewer we added the citation “Gowaty PA, Drickamer LC, Schmid-Holmes S. Male house mice produce fewer offspring with lower viability and poorer performance when mated with females they do not prefer. Anim. Behav. 2003;65: 95-103”.
13) Page 3 line 59: as suggested by the reviewer we added the citation “Ah‐King, M., & Gowaty, P. A. (2016). A conceptual review of mate choice: stochastic demography, within‐sex phenotypic plasticity, and individual flexibility. *Ecology and evolution*, *6*(14), 4607-4642”.
14) Page 4 line 69: as suggested by the reviewer, we added “giving primary importance to survival over reproductive productivity” to specify behaviors.
15) Page 4-5 lines 85-90: as suggested by the reviewer we suppressed the unnecessary sentence and add references (Beach FA and Leboeuf BJ 1967, Coital behaviour in dogs. I. Preferential mating in the bitch. Animal behaviour, 15(4) 546-548, Sánchez-Macouzet O, Rodríguez C, Drummond H. 2014 Better stay together: pair bond duration increases individual fitness independent of age-related variation. Proc. R. Soc. B 281: 20132843).
16) Page 4-5 lines 85-90: As suggested by the reviewer in the discussion, we added the following paragraph in the introduction “There are, for example, cases where behavioral similarity is the key to reproductive success, as in the case of zebra finches (*Taeniopygia guttata*) and cockatiels (*Nymphicus hollandicus*) [31-32] ; while, in other species, different or even opposite temperaments can reach high reproductive performances as in the giant panda (*Ailuropoda melanoleuca)* and in the black rhino (*Diceros bocornis)* [12, 25, 33]*.”*
17) Page 5 line 91: we created a new sentence as suggested by the reviewer deleting “and the study shows that”
18) Page 5 line 98: as suggested by the reviewer we changed boldness for reactivity.
19) Page 5 line 105: as suggested by the reviewer we changed boldness for reactivity.
20) Pace 5 line 110: we changed “attempt to create” with “creating” as suggested by the reviewer.

**Material and Methods**

21) Page 6 line 125: we deleted the hyphenation in “North-western”.
22) Page 7 line 146: we change the tense of “avoid” to “avoided”.
23) Page 7 line 147: we were talking about sperm storage that was studied in different turtles’ species but there aren’t any available work about this species. For a better understanding we changed the word “recollecting” with “storing”.
24) Page 8 line 167: during the trial we used water only to spraying the individuals, no pool or drinking point were used, therefore there was no reason to refill something. The water we used to spray was always new and after every trial we changed the position of the observation area into a dry one.
25) Page 10 line 228: we changed the tense from “will not be” to “were”.
26) Page 10 line 232: We used “sequentially” to explain that the experiments were done one after the other, but the term is maybe not adequate. We deleted this word to avoid confusion.

**Results**

27) Page 12 line 262: we changed the word “organisms” with "turtles” as suggested by the reviewer.
28) Page 12 line 265: we changed “didn’t” with “did not” as suggested by the reviewer.
29) Page 12 line 273: as suggested by the reviewer we change “for the shier” by “characteristic of the shier turtles”.
30) Page 12 line 276: As suggested by the reviewer we changed “for the boldest” by “. Variables for the boldest turtles were”.

**Discussion**

31) Page 20 line 361: we changed the beginning of the sentence from “Also, our” to “Our” as suggested by the reviewer.
32) Page 21 line 386: we deleted “the”.
33) Page 21 line 386: we added “animals”.
34) Page 21 line 393-394: according to referee’s commentary, we add in the sentence that it was observed for other species “In other species, male competition could be very costly”.
35) Page 22 lines 407-408: in order to rewrite the sentence as suggested by the reviewer, we changed “respond” with “adapt” and we delete “pass under the clutches of” to add “survive to”.
36) Page 22 line 418: according to the reviewer comment we suppressed” behavioral trait” and added “temperament” to be more specific in our affirmation.
37) Page 22: we deleted the paragraph “In the study of Spoon *et al* (2006), it can be seen that compatible pairs (male-female) of the Cockatiel *Nymphicus hollandicus* showed higher levels of coordination in parental care and raised more chicks; another example is given by Carlstead *et al* (1999a, b) who observed in Black Rhino *Diceros bocornis* that smaller enclosures determined the breeding of dominant males, while domination decreased in males housed in larger enclosures. In addition, dominance levels were related to reproductive success, with the conclusion that the number of births per year was higher in pairs of rhinos composed of a submissive male and a dominant or aggressive female. This information gives an idea of the optimal conditions for the breeding of captive rhinos, including large enclosures, which facilitate submissive behavior in males, which in turn affects the compatibility of couples; in the giant panda *Ailuropoda melanoleuca*, Martin-Wintle *et al* (2017) conducted behavioral studies to further improve reproductive performance and found that specific combinations of temperament traits showed better reproductive performance than others. Males who were more aggressive than their partners were more likely to mate and produce puppies than when the female had a higher level of aggression than the male or even couples with less fearful males, regardless of the fear of the female, obtained better reproductive results. The application of these results to captive breeding management strategies should result in higher reproductive rates and the production of more candidates for the giant panda reintroduction program.” and added a resumed version in page 4 lines 95-100.
38) Page 22 lines 428: we deleted “focused on boldness degree of males”, same with “therefore they could have a” and added “with perhaps”.
39) Page 23 lines 435 and 437: we deleted “has allowed to understand” and added “demonstrates” and added “animals”.
40) Page 23: as suggested by the reviewer and to avoid repetition of the same idea, we deleted the sentence “By another way in term of evolution, being shiest may have some advantages and, if not for reproduction, they may be for antipredatory behaviour, making shiest individuals, that are more prudent in term of exploration, and as we observed in the new environment, generally less predated than boldest individuals as discussed for other species.”
41) Page 24: as suggested by the reviewer we deleted the last paragraph.

**Bibliography**

42) Following the suggestions of the reviewer we made changes in the citations. We added the following works:

- Gowaty PA, Drickamer LC, Schmid-Holmes S. Male house mice produce fewer offspring with lower viability and poorer performance when mated with females they do not prefer. Anim. Behav. 2003;65: 95-103.
- Ah‐King M, Gowaty PA. A conceptual review of mate choice: stochastic demography, within‐sex phenotypic plasticity, and individual flexibility. Ecol. Evol. 2016;6: 4607-4642.
- Beach FA and Leboeuf BJ Coital behaviour in dogs. I. Preferential mating in the bitch. Anim Behav. 1967; 15: 546-548.
- Sánchez-Macouzet O, Rodríguez C, Drummond H. Better stay together: pair bond duration increases individual fitness independent of age-related variation. Proc. R. Soc. B. 2014; *281*: 20132843.

And deleted:

- Cable RN. Oh, Behave: When wildlife behavior matters in Conservation. Mich. J. Sustain. 2013;1
- Cooper Jr WE. Variation in escape behavior among individuals of the striped plateau lizard *Sceloporus virgatus* may reflect differences in boldness. J. Herpetol. 2009;43:495-502.
